# A Bichromophoric Triazatruxene Tetrad as a Highly Tunable Multicolor Emitter and Its Application in OLEDs

**DOI:** 10.1002/chem.202501992

**Published:** 2025-08-30

**Authors:** Lars Vogelsang, Muhammad Irfan Haider, Kai Vogelsang, Tobias Seewald, Azhar Fakharuddin, Niklas Bauch, Gabriel Maier, Katharina L. Deuter, Tobias Birk, Mikhail Fonin, Lukas Schmidt‐Mende, Rainer F. Winter

**Affiliations:** ^1^ Fachbereich Chemie Universität Konstanz 78457 Konstanz Germany; ^2^ Fachbereich Physik Universität Konstanz 78457 Konstanz Germany

**Keywords:** emission, OLED, phosphorescence, spectroelectrochemistry, triazatruxene

## Abstract

We report on the new star‐shaped triazatruxene (TAT) tetrad **1**, where three peripheral TAT moieties connect to a central TAT through propargylic spacers. The insulating nature of the linkers results in separate, partially overlapping blue and yellow‐orange emissions from the peripheral and central TATs. This renders **1** a multicolor emitter, whose emission color can be tuned by the choice of the excitation wavelength and the solvent. In frozen solution, dual fluorescence emission from the two different types of TATs is complemented by dual phosphorescence with radiative lifetimes in the range of seconds. Organic light‐emitting devices (OLEDs) constructed with **1** as the emissive layer achieved a peak irradiance of 5.87 µW/nm/m^2^ at 580 nm at an operation voltage of 6.7 V. Remarkably, the emission color of the electroluminescence can be varied from yellow over different hues of orange to red or even to near infrared (NIR) emission, depending on the applied voltage. Tetrad **1** is also redox‐active, indicating that **1** may simultaneously serve as a hole conductor and emitter. Its oxidized forms are panchromatic absorbers from the near UV to the NIR due to intra‐ and inter‐TAT charge‐transfer (CT) absorptions.

## Graphical abstract

We report on a TAT tetrad, whose propargylic linkers electronically decouple the central TAT unit from the peripheral ones. This results in dual fluorescence and, at 77 K, dual phosphorescence emission. The emission color varies with excitation wavelength from blue to yellowish white or orange.

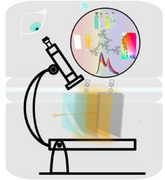

## 1 Introduction

For the past 20 years, large‐area flat‐panel display technology for TV and computer screens has relied mostly on liquid crystal displays (LCDs).^[^ ^1^, ^2^, ^3^, ^4^ ^]^ The vastly increasing number of displays and the demand for flexible screens fuel a rapidly increasing surge for advanced technologies,^[^ ^5^, ^6^ ^]^ in particular for organic light‐emitting devices (OLEDs).^[^ ^7^, ^8^, ^9^ ^]^ OLEDs typically have a multilayer design consisting of the electrodes, separate electron‐ and hole‐injection, transport and charge‐blocking layers, as well as the emissive layer where the electrons and holes recombine. They thereby generate an electronically excited state of the emitter that ultimately translates the charge input into the visible light output. While their higher fabrication costs presently limit advanced OLED technology to small‐screen devices such as high‐end smartphones, OLED‐powered screens have several advantages over common LCD displays. They are independent on background illumination and produce high‐contrast images, irrespective of the viewing angle.^[^ ^10^ ^]^ Furthermore, the individual lighting of each pixel allows for brilliant black coloration and reduces energy consumption.^[^ ^11^, ^12^ ^]^ This however, comes with issues such as burn‐in or sensitivity toward air and moisture.^[^ ^13^, ^14^, ^15^ ^]^

**Figure 1:**
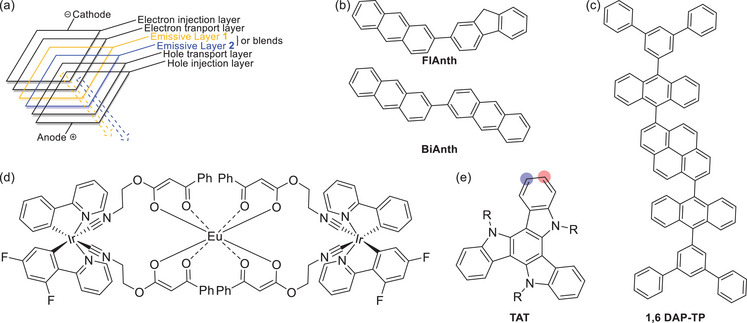
a) Schematic construction of a blended WOLED. b) Covalently linked bianthracene (BiAnth) and fluorene‐anthracene (**FlAnth**) as multicolor emitters. c) **1,6‐** **DAP‐TP** as an example of a polycyclic aromatic hydrocarbon capable of producing mixed emissions from individual molecules and excimers and generating a white‐light impression. d) A heterotrimetallic Ir_2_Eu‐complex with electronically isolated emissive units to generate overall white‐light emission. e) Generic structure of a triazatruxene. The 2‐position is marked in red, and the 3‐position in blue color.

A further level of sophistication is to use OLEDs with dyes that emit light of different wavelengths in response to the applied bias, thereby offering multicolor and, most importantly, white‐light emission. The latter may be achieved by combining two different fluorophores or a fluorophore and a phosphor, which emit light of complementary colors, where the emission of the second emitter is triggered by the light emitted from the other fluorophore.^[^ ^16^ ^]^ This design principle however faces challenges associated with the trapping of photons inside the device and with size scaling due to highly intricate fabrication processes.^[^ ^17^, ^18^, ^19^, ^20^, ^21^ ^]^ A related approach to fabricate hybrid white light‐emitting OLEDs (WOLEDs) is to employ judiciously chosen blends of ternary mixtures of red, green, and blue (RGB) emitting dyes or dye‐modified polymers^[^ ^22^, ^23^ ^]^ as separate emissive layers (EMLs, see Figure 1a) in a stacked geometry.^[^ ^24^ ^]^ A certain simplification can be achieved by blending structurally related dyes such as covalently linked polycyclic arenes like **FlAnth** and **BiAnth** (see Figure 1b) into a single emissive layer.^[^ ^25^ ^]^ Still, the production of WOLEDs remains challenging and requires sophisticated device fabrication procedures to guarantee homogenous distributions of the different dyes and well‐balanced light emission.

Single‐molecule white‐light emitters promise significant simplification of the production process but shift the efforts from optimizing the blending of different emitters to smart molecular engineering in order to design molecules whose emissions cover a large part of the visible spectrum. One possible realization is the use of compounds that are prone to aggregation. White‐light emission then relies on the delicate balancing of monomer and excimer emissions from the aggregates^[^ ^26^ ^]^ whose formation is driven by attractive intermolecular forces such as π–stacking interactions, hydrogen bonding, and electrostatic or van der Waals interactions.^[^ ^27^, ^28^, ^29^, ^30^ ^]^ One class of molecules that qualify for this purpose are polycyclic aromatic hydrocarbons (PAHs) such as **1,6‐DAP‐TP**, whose molecular structure is shown in Figure 1c.^[^ ^27^ ^]^ An alternative design for single‐molecule white‐light emitters is dually emissive donor‐acceptor‐type dyads comprising two electronically decoupled luminophores with complementary emission colors. This type of white‐light emitter is particularly attractive since it obviates the requirements of multiple emissive layers or of maintaining well‐balanced concentrations and mixing of different emitters in WOLED production. Known examples for such kinds of emitters are mixed Eu(III)‐Ir(III)‐complexes with bi‐ or terpyridyl and elaborate β‐ketoenolate ligands, like the specific example shown in Figure 1d.^[^ ^31^, ^32^, ^33^ ^]^ Other design principles of white‐light emitters build on combining direct fluorescence emission with thermally activated delayed fluorescence (TADF) or on harnessing dual fluorescence and phosphorescence emissions of complementary color from different, noninteracting ligands in transition metal complexes.^[^ ^34^, ^35^, ^36^, ^37^, ^38^, ^39^, ^40^, ^41^ ^]^

Triarylamines (TAAs) are an important class of OLED emitters, as they allow for emission color tuning over the entire visible (vis) range and for producing RGB‐type OLEDs from blends of red‐, green‐, and blue‐emitting TAAs.^[^ ^42^, ^43^, ^44^, ^45^, ^46^ ^]^ In addition, they may also serve as electron donors in the hole‐transport layer (HTL). Polycyclic aryl‐substituted amines (PAAs) constitute an important subclass of TAAs, which are frequently applied as (dopant‐free), cost‐effective hole‐conducting materials in OLEDs or lithium‐ion batteries, or as one‐component hole conductors and emitters in (organic) solar cells.^[^ ^47^, ^48^, ^49^, ^50^, ^51^, ^52^, ^53^, ^54^, ^55^, ^56^ ^]^ Triazatruxenes (TATs; see Figure 1e for a schematic drawing) are prominent representatives of PAAs. Their planar, π‐extended, *C*_3_ symmetric skeleton can be envisioned as consisting of three indoles that are fused through a common phenyl ring and offers superior π‐conjugation when compared to open, propeller‐shaped TAAs.^[^ ^57^ ^]^ TATs can be easily deposited on substrates by vapor deposition or solution film processing techniques, which is highly beneficial for the later processing of operational devices.^[^ ^56^, ^57^, ^58^, ^59^ ^]^ If appropriately substituted, they are capable of forming electrically conductive columnar phases or mesophases. They thus constitute organic semiconductors, whose conductivity can be tuned by the addition of an appropriate oxidant or by applying an appropriate bias voltage.^[^ ^60^, ^61^, ^62^ ^]^ TATs are also intensively studied for their photo‐ (PL) and electroluminescent (EL) properties, whose absorption and emission profiles can be adjusted by the choice of substituents at either the amine N atoms or the 2‐ and 3‐positions of their molecular scaffold.^[^ ^40^, ^63^, ^64^, ^65^ ^]^

Considering the above, we mused that dyads or tetrads, where differently substituted TATs are interconnected by insulating linkers, might exhibit multicolor emission from a single molecule, or even white‐light emission. We here report on the successful realization of this concept and present the TAT tetrad **1,** whose emission color can be tuned from green to yellowish white, depending on the solvent and the excitation wavelength. Moreover, under cryogenic conditions, **1** exhibits separate phosphorescence emissions from its two different chromophores, with a second‐long afterglow. Tetrad **1** was also utilized for the construction of OLEDs with stable yellow and near infrared (NIR) emission. The properties of **1** are rationalized by comparison with model compounds **2** and **3** that resemble the inner and peripheral TAT units of the tetramer and by quantum chemical calculations. Oxidized **1** produces panchromatically absorbing mixed‐valent radical cations with continuous extinction from the near UV to the near infrared(NIR).

## 2 Results and Discussion

### 2.1 Synthesis

2‐Ethynyl‐*N*,*N*',*N*''‐triethyl‐TAT (**2‐A_1_‐^Et^TAT**), which was already utilized as a building block for the construction of asymmetric **^Et^TAT** scaffolds,^[^ ^66^, ^67^ ^]^ serves here as the common precursor for TAT tetrad **1** and dyad **2**. The acetylide resulting from treatment of **2‐A_1_‐^Et^TAT** with *^n^* BuLi was reacted with 2,2′,2′’‐triformyl‐**^Et^TAT** (**2‐CHO_3_‐^Et^TAT**)^[^ ^64^ ^]^ in a 3.4:1 molar ratio to give compound **1** as a red microcrystalline solid. Tetrad **1** is well soluble in CH_2_Cl_2_, tetrahydrofuran, DMSO, chlorobenzene, dimethylformamide, and toluene, but less so in acetonitrile, diethyl ether and *^n^* alkanes. NMR and IR spectra of **1** lack the characteristic ≡C‐H and C = O stretching modes of the starting compounds at 3304 cm^−1^ (ν (≡C‐H)) and 1688 cm^−1^ (ν(C = O)) and the split vibrational band of the formyl C‐H unit resulting from the Fermi resonance of the O = C‐H stretching and the first overtone of the in‐plane C‐H bending modes. The latter bands are located at 2830 cm^−1^ and 2720 cm^−1^ in **2‐CHO_3_‐^Et^TAT**. This confirms alkyne addition to all three formyl functionalities of **2‐CHO_3_‐^Et^TAT**. IR spectra recorded on a solution of tetrad **1** feature a single ethynyl C≡C stretching vibration for an internal trialkyne at 2183 cm^−1^, which is shifted to 2187 cm^−1^ in a KBr‐pellet or to 2190 cm^−1^ in the FT‐ATR‐IR (see Figures S29–S36 of the Supporting Information). This result is well‐matched by quantum chemical calculations, which predict three nearly isoenergetic C≡C stretching modes (see Figures S86 and S87 of the Supporting Information).

**Scheme 1:**
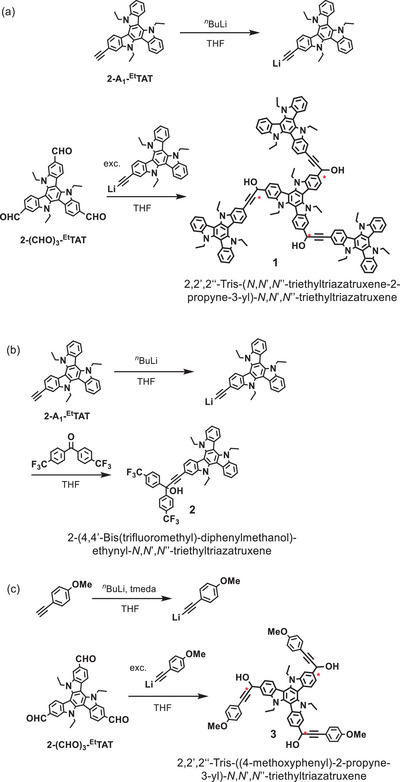
Synthesis of (a) tetrad 1 and (b), (c) mono‐TAT model compounds **2** and **3**; the red asterisks mark the three stereocenters of compounds **1** and **3**.

We also prepared compounds **2** and **3** of Scheme 1b, c) as benchmark models for the peripheral and the central TAT constituents of **1**. Carbinol **2** was obtained by reacting in situ lithiated **2‐A_1_‐^Et^TAT** with an excess (1.7 equiv.) of 4,4′‐bis(trifluoromethyl)benzophenone, whose carbonyl C atom was found to be sufficiently electrophilic to allow for the attack of the ethynyl nucleophile. As the electron‐rich TAT attenuates the electrophilicity of the aldehyde carbonyl C atoms of **2‐CHO_3_‐^Et^TAT**, we employed *p*‐anisyl acetylide as a sufficiently strong nucleophile to generate model compound **3**.

Detailed synthesis procedures as well as the ^1^H‐, ^13^C{^1^H}‐, and DOSY‐NMR and mass spectra of **1**, ^1^H‐ and ^13^C{^1^H}‐NMR and mass spectra of **2**, and ^1^H‐ and DOSY‐NMR spectra for **3** are provided in the Supporting Information (see Figures S4 to S16). Owing to the presence of three chiral centers at the propargylic C atoms (marked by red asterisks in Scheme 1a), compounds **1** and **3** were obtained as mixtures of four enantiomers, *R*,*R*,*R*, *S*,*S*,*S*, *R*,*R*,*S*, and *R*,*S*,*S*. Moreover, both compounds have a high tendency to dimerize. This severely aggravates the unambiguous characterization of **1** and **3** by NMR spectroscopy, as all resonances are broad, unresolved multiplets (Figures S4 and S12 of the Supporting Information). Diffusion‐ordered (DOSY) NMR spectra of **1** provided an estimated molecular weight MW of 3744 g⋅mol^−1^ for the main species, which is strongly suggestive of dimers in CDCl_3_ solution (see Figure S14 of the Supporting Information; the calculated mass of a dimer is 3748.72 g⋅mol^−1^). The molecular weight of the minor fraction of 1890 g⋅mol^−1^, as estimated from the diffusion coefficient, falls close to the theoretical value of a monomer of 1874.36 g⋅mol^−1^. DFT calculations performed on the exemplary *R*,*S,S* enantiomer indicated that monomers of **1** adopt a stool‐like structure in solution with the inner TAT as the seat and the peripherally appended TATs as three legs (see Figure S85 of the Supporting Information). Geometry optimization also yielded dimers that are by 105.4 kJ/mol more stable than isolated monomers with the inner TATs in a nearly coplanar, laterally slightly offset arrangement. Dimerization is driven by three O‐H⋅⋅⋅O hydrogen bonds between the hydroxyl functionalities as shown in Figure S85 of the Supporting Information, as well as by dispersion interactions. DOSY measurements on compound **3** provided evidence for even higher oligomers formed by (an average of) nine molecules (see Figure S16 of the Supporting Information). IR spectra of compound **3** also lack a ≡C‐H stretching vibration, but feature the C≡C stretching mode of an internal alkyne at 2187 cm^−1^ as a KBr pellet. While hard to obtain, mass spectra reveal peaks with matching isotopic distributions at 936.94 amu that we tentatively assign as **1^2+^** and {**1** ‐ H}^2+^ for **1**, and at 772.27 amu, corresponding with **2^+^** and {**2** + H}^+^ for **2**.

Ultimate proof of the identity of **1** as a TAT tetramer comes from topographic images obtained from scanning tunneling microscopy on molecules of **1** on Ag(111) (see Figure S76 of the Supporting Information). Individual molecules have a lateral length of about 4.50 nm and an apparent height of about 1.10 nm, indicating that they flatten out on the Ag(111) substrate. Corresponding with strong intermolecular interactions, molecules of **1** form dense layers. Such self‐assembly is advantageous for film formation. Our deposition experiments also revealed that molecules of **1** are susceptible to cleavage at the alkynyl linkages under the conditions of electrospray deposition (see the Supporting Information for experimental details), which also explains our difficulties in obtaining mass peaks of intact tetramers.

### 2.2 Optical, Electrochemical, and Photophysical Properties

**Figure 2:**
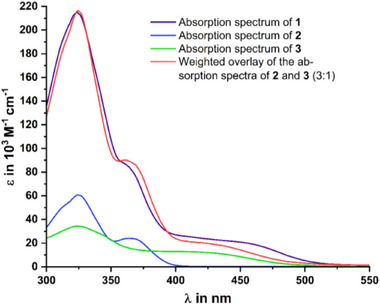
UV/vis spectra of compounds **1**–**3** as well as the hypothetical spectrum of **1** calculated as the weighted sum of the spectra of model compounds **2** and **3**.

TATs offer allowed π→π* transitions and absorb strongly in the near UV. The relevant frontier molecular orbitals (MOs) HOMO to HOMO‐2 and LUMO to LUMO + 2 (HOMO = energetically highest occupied MO, LUMO = energetically lowest unoccupied MO) of symmetrically trisubstituted TATs are nearly degenerate and extend over all three indolyl sidearms and the central benzene ring.^[^ ^68^, ^69^, ^70^ ^]^ Splittings between these MOs increase in mono‐ or disubstituted TATs due to symmetry lowering. The electronic absorption spectrum of carbinol‐functionalized TAT **2** features a prominent band at 323 nm and a resolved, weaker band at 365 nm (see Figure 2). According to our TD‐DFT calculations at the PBE1PBE level of theory, the electronic transition underlying the more red‐shifted absorption band involves charge‐transfer (CT) from the electron‐rich TAT to the electron‐deficient trifluoromethyl‐substituted phenyl rings at the carbinol C atom. CT excitations of the same kind also contribute to the absorption at higher energy but are admixed with π→π* excitations that are confined within the TAT chromophore, as illustrated by the electron density difference maps (EDDMs) in Figure S89 in the Supporting Information. The main absorption peak of compound **3**, which represents the trisubstituted core TAT unit of tetrad **1**, appears at 324 nm, nearly at the same wavelength as in **2**. Our TD‐DFT calculations attribute this band to several close‐lying π→π* excitations between MOs that are confined within the TAT chromophore, but with additional contributions from TAT→ethynylanisole CT. Accounting for the red color of **3**, an additional, weak band is located at ca. 425 nm, with an onset at 520 nm.

The electronic absorption spectrum of **1** resembles the weighted sum of those of its constituents rather closely (see Figure 2), and most computed transitions are localized on one of the two different kinds of TAT units (see Figure S93 of the Supporting Information). We, however, note an increased width and tailing of the low‐energy part of the spectrum, which results in a plateau‐like absorption extending to 520 nm, that is, to lower energies as compared to models **2** and **3**. Lambert‐Beer plots of UV/vis spectra recorded on solutions of these compounds in CH_2_Cl_2_ at different concentrations provided nearly linear, uniform absorbance increases with concentration for all bands (see Figures S26 and S27 of the Supporting Information). The red‐shift of the low‐energy features from **3** to **1** suggests that they derive from CT between the propargylic substituents and the TAT. We however note that our TD‐DFT calculations place corresponding excitations at higher energies than in the experimental spectrum. Differential conductance (d*I/*d*U*) measurements performed on each of the four units of **1** on the Ag(111) surface show substantial differences between the peripheral and central units (see Figure S77 of the Supporting Information). Peripheral ^Et^TATs show a pronounced spectral feature at about 2 eV, which can be associated with the LUMO. The central ^Et^TAT unit is associated with an additional feature at about 1 eV, thus pointing at a substantial energy shift of the LUMO toward *E*_F_. This is in line with the trends in electronic absorption spectra.

**Figure 3:**
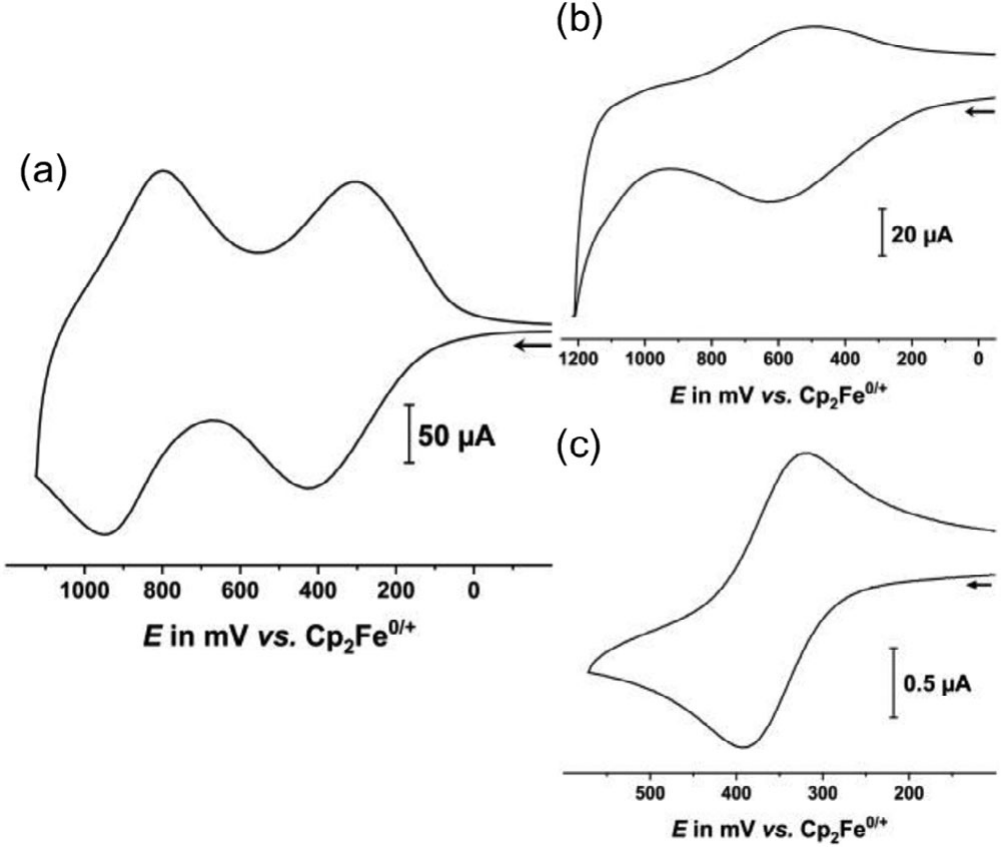
Cyclic voltammograms of a) **1** at *v* = 0.1 V/s, b) **3** at *v* = 2.0 V/s, and c) **2** at *v* = 0.1 V/s in CH_2_Cl_2_/NBu_4_^+^ [BArF_24_]^−^ (0.04 m) and at r. t.

Widespread applications of PAAs as hole‐conducting materials in OLED devices motivated us to study the redox properties of TATs **1**–**3** by means of cyclic and square wave voltammetry. Considering its very weak ion‐pairing capabilities, we employed the CH_2_Cl_2_/NBu_4_^+^ [BAr^F24^]^−^ (0.04 m) electrolyte^[^ ^71^, ^72^, ^73^ ^]^ in order to render also their higher charged forms soluble and to possibly maximize redox splittings between consecutive redox steps. Figure 3 shows representative cyclic voltammograms recorded at r. t. Pertinent data of the present compounds and of related TATs are collected in Table 1. The half‐wave potential of the first oxidation of **2** at 288 mV differs insignificantly from that of **^Et^TAT** and is 43 mV lower than that of **2‐A_1_‐^Et^TAT**.^[^ ^66^ ^]^ The expected second oxidation of **2** falls however outside the anodic limit of the used electrolyte, while it is close to 900 mV for **^Et^TAT** and **2‐A_1_‐^Et^TAT**, respectively. We attribute this anodic shift to the electron‐withdrawing propargylic substituent of **2**. Compound **3** merely shows an unusually broad, chemically only partially reversible voltammetric wave at a half‐wave potential of 558 mV (at *v* = 2000 mV/s). The broadness of this feature is likely related to the presence of aggregates of **3** in solution (see above) (Table 2).

**Table 1:** CV Data of tetrad **1**, model complexes **2** and **3**, and related TATs from the literature in CH_2_Cl_2_/NBu^4+^ [BArF_24_]^−^ (0.04 m) at r. t. Potentials are referenced to the Cp_2_Fe/Cp_2_Fe^+^ standard.

	*E*_1/2_ [TAT^0/+^][^[a]^, ^[b]^]	*E*_1/2_ [TAT^+/2+^][^[a]^, ^[b]^]	*ΔE*_1/2_
**^Et^ TAT** ^[^ ^66^ ^]^	298	914	616
**1^a^**	366^[^ ^[a]^, ^[b]^ ^]^	892 ^[^ ^[a]^, ^[b]^ ^]^	526
**2**	365	‐	‐
**3^a^**	558^a)^	‐	‐
**2‐A_1_‐^Et^TAT^[^** ^66^ **^]^**	331	897	566
**2‐A_3_‐^Et^TAT^[^** ^66^ **^]^**	385	‐	‐
**2‐CHO_3_‐^Et^TAT**	621	1131	510

^[a]^ Mean value.

^[b]^ Every redox process involves all TAT units of the respective compound.

Tetrad **1** exhibits two multi‐electron redox processes at average half‐wave potentials of 366 and 892 mV. Judging from the ratios of the reverse (cathodic) and the forward (anodic) peak currents, both waves appear to be chemically reversible, or nearly so, whereas the sharper return peak of the composite second wave is suggestive of solubility issues and adsorptive behavior of the final oxidation product. We tentatively assign the two waves to the stepwise oxidations of all four TAT constituents to first give **1^4+^** and then **1^8+^**. Voltammetric measurements with decamethylferrocene (Cp*_2_Fe) as the internal redox standard confirmed the 4‐electron character of the first oxidation process (see Figure S22 and the discussion in the Supporting Information).

**Figure 4:**
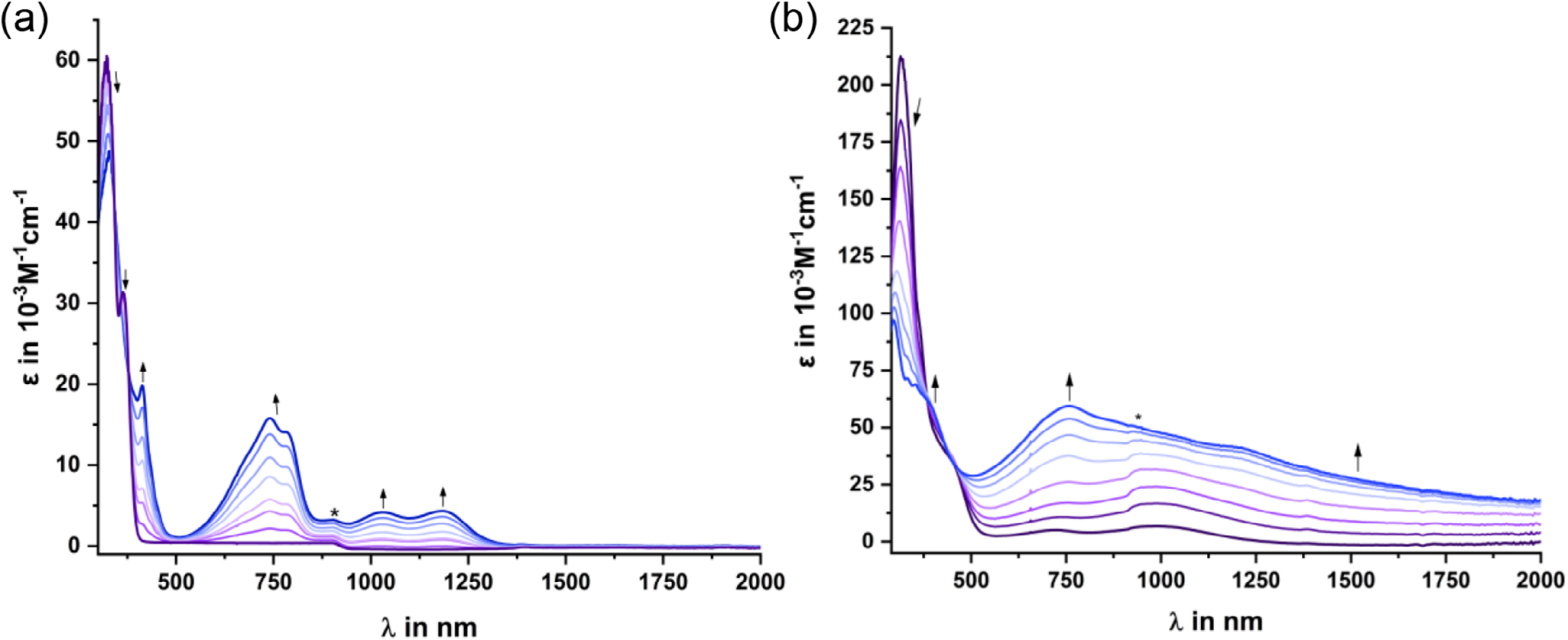
Changes in the UV/vis/NIR spectrum of a) **2** and b) **1** in the 0.14 m 1,2‐C_2_H_4_Cl_2_/NBu_4_^+^ [BArF_24_]^−^ electrolyte at r. t. as recorded during electrolysis in an OTTLE cell at a potential past the **2** ^0/+^ or the **1** ^0/4 +^ redox couple. The asterisk marks an artifact of our instrumentation caused by a detector switch.

The low degree of chemical reversibility of compound **3** prevented us from monitoring the spectroscopic changes concomitant with oxidation. No such problems were encountered for carbinol **2**. Electrochemical or chemical oxidation to radical cation **2^+^** caused the appearance of new absorption bands at 413, 740, 900, 1030, and 1190 nm, while the band at 323 nm decreased in intensity (see Figure 4a and Figure S28 of the Supporting Information). Our TD‐DFT calculations attribute the low‐energy features as well as the band at 413 nm to intervalence charge‐transfer (IVCT) transitions that are confined within the oxidized TAT chromophore, which constitutes an electronically delocalized mixed‐valent system.^[^ ^74^ ^]^ Excitation into these bands shifts electron density from the remaining reduced indolyl subunits of the TAT core to the oxidized one. The transition at 740 nm also has partial IVCT character but is mingled with CT from the (4‐F‐C_6_H_4_)_2_C(OH)‐C≡C‐ moiety to the oxidized TAT, as suggested by the involved molecular orbitals and electron density difference maps (see Figure S90 of the Supporting Information).

Oxidized forms of tetrad **1** show largely similar but significantly more convoluted spectra than **2^+^** that also extend further into the NIR. We noted a continuously increasing absorbance over the entire region from 500 nm to well over 2000 nm during the entire redox process until full oxidation to **1^4+^** was attained, where all TAT subunits are oxidized once. This renders particularly the higher oxidized forms **1^n+^** (n = 1‐4) panchromatic absorbers with continuous light absorption from the near UV well into the NIR. Identical spectral changes ensued during redox titrations with Ag^+^ SbF_6_^−^ as the oxidant. One should note here that the close proximity of the individual redox waves of **1** prevents us from assigning any spectrum recorded until full conversion to **1^4+^** to any defined redox species **1^n+^** as several different oxidation states will coexist in solution. According to our TD‐DFT calculations on **1^4+^**, assuming a quintet ground state, several types of transitions contribute to the broad envelope, among them π→π* bands that are confined to individual open‐shell TAT^+^ chromophores and excitations that involve the transfer of charge between the inner and the peripherally appended TATs (see Figure S95 of the Supporting Information). Complementary to changes in the electronic spectra, the original C≡C stretching vibration of **1** at 2183 cm^−1^ was gradually replaced by a new, broader C≡C band at 2304 cm^− 1^, which first gains and then decreases in intensity as the oxidation proceeds. The latter band was resolved into two separate bands at 2314 cm^−1^ and 2286 cm^− 1^ for samples that were generated by chemical oxidation with Ag^+^ SbF_6_^−^. Precursor alkyne **2‐A_1_‐^Et^TAT** and dyad **2** behaved similarly (see Figures S28, S37, and S38 of the Supporting Information). No higher oxidized forms beyond **1^4+^** were, however, accessible by chemical or electrochemical oxidation. Attempts to enforce this process by increasing the potential at the working electrode to past the **1^4+/8+^** wave resulted in rapid electrode passivation.

**Table 2:** Experimental and TD‐DFT calculated UV/vis data (*λ*_max_ [nm]) of tetrad **1** and of model compounds **2** and **3** in different oxidation states.

Neutral			Oxidized		
**1**	exp. calcd. calcd.	324, 360, 430 **1**: 313, 351 **1–1**: 314 353, 355	**1^4+^**	exp.	295, 398, 430, 757, 1025,1050–2000
**2**	exp. calcd.	323, 365 312, 352	**2^+^**	exp. calcd.	327, 413, 740, 900, 1030, 1190 305, 408, 781, 1021
**3**	exp. calcd.	324, 425 318			

**Figure 5:**
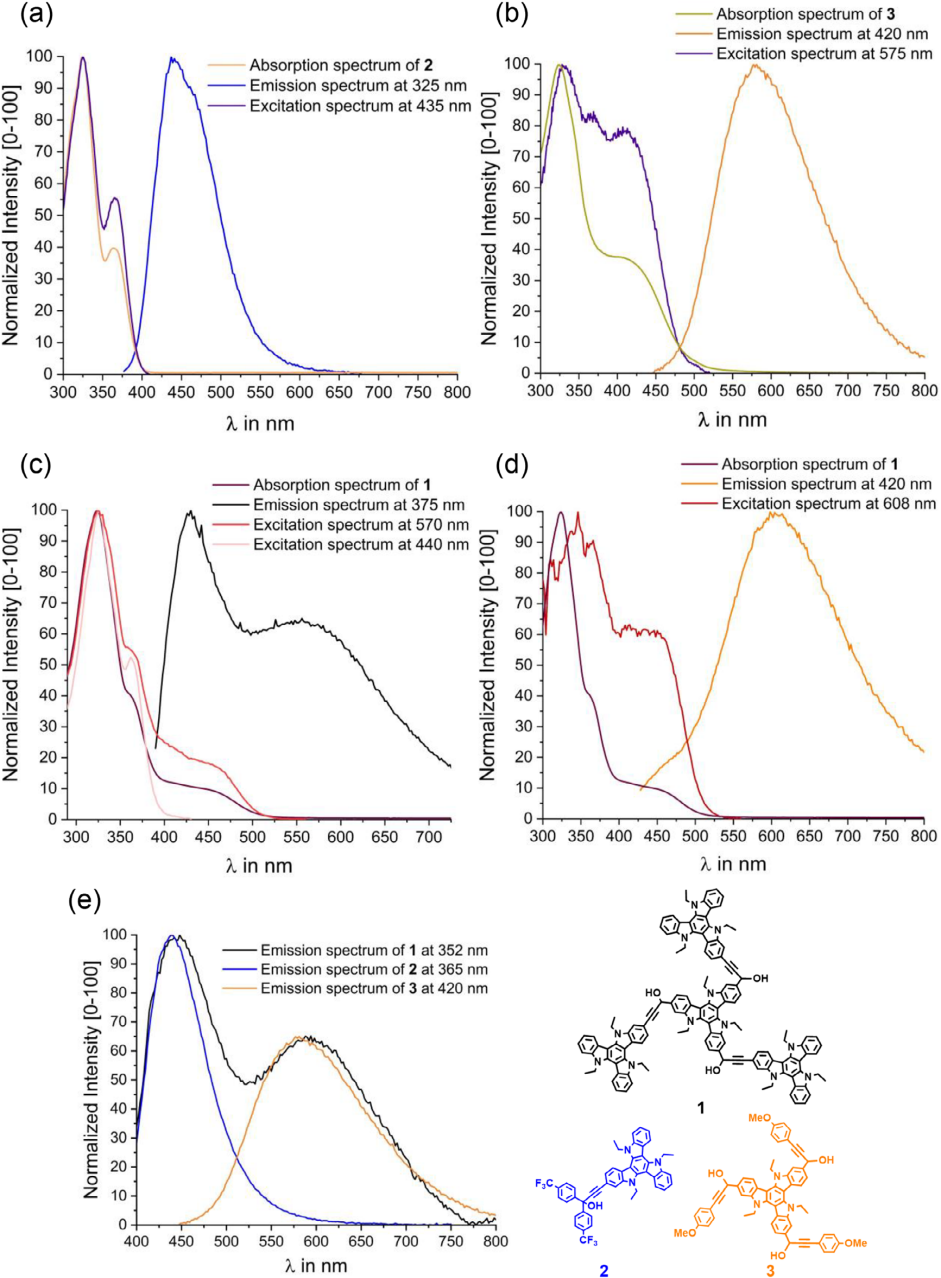
a) Normalized absorption, emission, and excitation spectra of compound **2** in CH_2_Cl_2_ at r. t. with excitation at *λ* = 325 nm. The excitation spectrum was recorded at the maximum of the emission peak at 435 nm. Coordinates for the royal blue emission are x = 0.16, y = 0.12; R = 0, G = 94, B = 224; #005DE0. b) Normalized absorption, emission and excitation spectra of compound **3** in CH_2_Cl_2_ at r. t. recorded at an excitation wavelength of *λ* = 420 nm. The excitation spectrum was recorded at the maximum of the emission peak at 575 nm. Coordinates of the goldenrod emission are: x = 0.50, y = 0.48; R = 238, G = 175, B = 0; #EBB000. c) Normalized absorption, emission, and excitation spectra of tetrad **1** in CH_2_Cl_2_ at r. t. with excitation at *λ*_exc_ = 375 nm and corresponding excitation spectra recorded at the maxima of the emission bands at 428 nm and at 574 nm. CIE coordinates are x = 0.28, y = 0.27; R = 141, G = 139, B = 176; #9C88AA, corresponding to light slate‐grey emission. d) Normalized absorption, emission, and excitation spectra of **1** in CH_2_Cl_2_ at r. t. recorded with *λ*_exc_ = 420 nm and excitation spectra recorded at the emission peak at 575 nm. CIE coordinates are x = 0.49, y = 0.44; R = 242, G = 160, B = 16. corresponding to the orange emission color. e) Comparison of the normalized emission of **1** with excitation at 352 nm (black line), of **2** excited at 365 nm (blue line), and of **3** on excitation at 420 nm (orange line) in CH_2_Cl_2_ at concentrations of 3‐6 µM. Emission intensities of **2** and **3** are normalized to the corresponding bands in the spectrum of **1**. Skeletal formulas of the compounds are shown in the same colors.

After having established the absorptive and electrochromic behavior of **1** and **2**, we now turn to the emissive properties of TATs **1**–**3** and of **2‐CHO_3_‐^Et^TAT**. Photophysical measurements were performed on 3–6 µM solutions in CH_2_Cl_2_ at r. t. as well as in the glassy state in 2‐methyl‐THF at 77 K, with different excitation wavelengths. Pertinent data are summarized in Table 2. As shown in Figure 5a, compound **2** emits blue light with a peak at 435 nm and a quantum yield (QY) of 30.7 % after excitation at 380 nm, or 22 % on excitation at 320 nm. The excitation spectrum recorded at the emission peak overlaps with the absorption spectrum, yet with enhanced intensity of the feature at 365 nm. Model compound **3**, which resembles the core TAT entity of tetrad **1**, shows goldenrod emission peaking at 575 nm with a QY of 4.8 % on excitation at 440 nm (Figure 5b). The excitation spectrum indicates that the emission is triggered by irradiation into any of the bands under the absorption envelope, again with enhanced intensity of the features at lower energies. Lifetimes *τ*_Fl_ of 3.67 ns for **2** and 4.12 ns and 1.52 ns for **3** mark the emissions as fluorescence. The emission of **3** is red‐shifted from that of the starting trialdehyde **2‐CHO_3_‐^Et^TAT** (λ_em_ = 502 nm, *τ*_Fl_ = 4.29 ns, see Figure S39 of the Supporting Information), while the emission lifetime is nearly identical.

**Figure 6:**
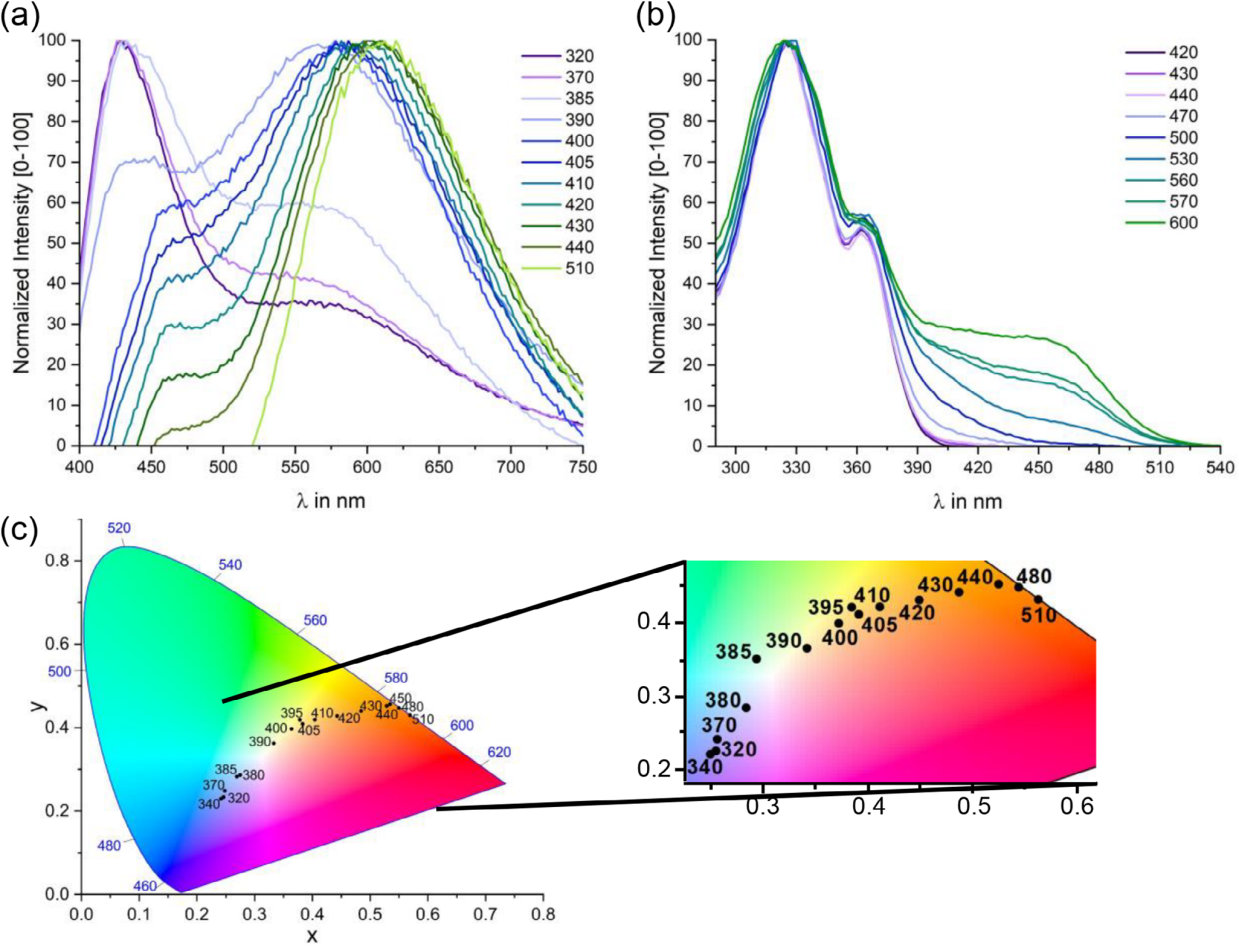
a) Normalized emission spectra of **1** after excitation with light of the indicated wavelengths. The spectra were recorded in CH_2_Cl_2_ at r. t. b) Normalized excitation spectra of **1** with detection wavelengths varying from 420 nm to 600 nm, measured under the same conditions as in a). c) Coordinates of the fluorescence emissions shown in the CIE chromaticity diagram (1931). An enlarged insert is shown on the right.

The notion that the absorption spectrum of tetrad **1** resembles an overlay of those of its two different constituents also pertains to the emission spectra (see Figure 5e). Upon excitation into the low‐energy tail of the absorption envelope, which likely entails CT from the peripherally appended TATs to the central TAT unit (vide supra), **1** emits dark orange light with an emission peak at 608 nm (*τ*_Fl_ = 1.43 and 4.80 ns), slightly red‐shifted to that of **3** (Figure 5d). Excitation spectra recorded at the maximum of this emission show a clear enhancement of the low‐energy features when compared to the absorption spectrum and, apart from a modest redshift, closely resemble the absorption spectrum of model compound **3**. This clearly indicates that the 608 nm emission emanates from the central TAT unit of tetrad **1**. When **1** is, however, excited at 375 or 320 nm, where both kinds of TAT constituents absorb, a second emission peaking at 428 nm emerges (Figure 5c, *τ*_Fl_ = 3.39 ns and 0.60 ns). Excitation spectra recorded at the position of the higher‐energy emission exhibit close similarity with the absorption spectrum of model compound **2**. The dual emission of **1** is hence a clear token of the presence of two types of mutually independent, electronically largely decoupled TAT fluorophores. As the two emissions from **1** are nearly complementary in color, they combine to near white‐light emission as shown in Figure 6. Even more remarkably, the emission color of **1** can be tuned from blue to bluish purple, turquoise, yellowish white, golden yellow, and orange because of the fact that the relative intensities of the emissions from the two different types of TATs depend on the excitation wavelength. Figure 6a,b) show emission spectra recorded at different excitation wavelengths and the corresponding excitation spectra collected from a CH_2_Cl_2_ solution at r. t., while Figure 6(c) provides CIE coordinates.

**Figure 7:**
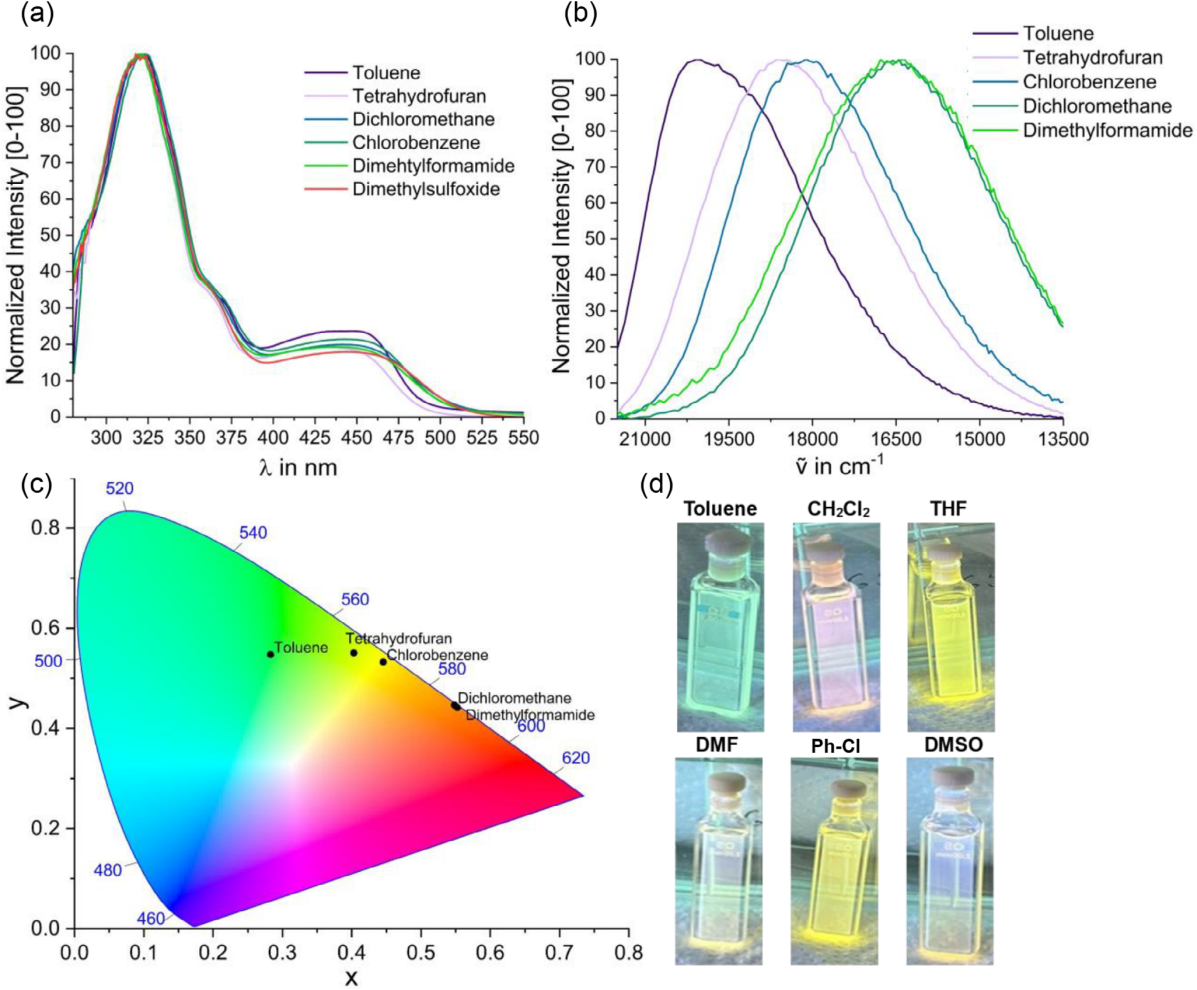
a) Normalized absorption spectra of compound **1** in different solvents at r. t. b) Normalized emission spectra of compound **1** in different solvents at r. t. recorded at an excitation wavelength of 450 nm. c) CIE plot (1931) of the PL spectra of **1** in different solvents under excitation at 450 nm, as shown in b): Toluene: x = 0.28, y = 0.55; R = 0, G = 226, B = 82; #00E252; lime green; tetrahydrofuran: x = 0.40, y = 0.55; R = 176, G = 210, B = 0; #B2D100; yellow‐green; chlorobenzene: x = 0.45, y = 0.53; R = 205, G = 199, B = 0; # CDC900; golden; dichloromethane: x = 0.55, y = 0.45; R = 255, G = 150, B = 0; #FF9500; dark orange; dimethylformamide: x = 0.55, y = 0.44; R = 255, G = 147, B = 0; #FF9500; dark orange. d) Visual impression of the emission color of **1** in different solvents at r. t. upon excitation with a 365 nm handheld UV lamp.

The absorptive and emissive properties of tetramer **1** can, however, not only be tuned by the excitation wavelength but also by the choice of the solvent. Two effects account for the solvent dependence: i) the solvatochromism of the absorption in the visible region of the spectrum, and ii) the differing ratios of absorbances from the inner and outer TATs at a given wavelength in different solvents. The former agrees with a partial CT character of the low‐energy absorptions of tetrad **1** (vide supra). Positional variations with solvent are amplified in the emission spectra as revealed by spectra recorded on solutions of **1** in different solvents under excitation at a uniform wavelength, e. g. at *λ*_exc_ = 450 nm as shown in Figure 7b. Thus the emission color of **1** changes from green (*λ*_em_ = 500 nm, 20 000 cm^−1^) in toluene, to greenish yellow (*λ*_em_ = 540 nm, 18 600 cm^−1^) in tetrahydrofuran, golden yellow in chlorobenzene (*λ*_em_ = 552 nm, 18 100 cm^−1^), and to orange (*λ*_em_ = 620 nm, 16 500 cm^−1^) in dimethylformamide and in CH_2_Cl_2_ (see Figure 7c).

The solvatochromic nature of the emission of **1** is further underpinned by the collection of photographs in Figure 7d that visualize the appearance of solutions under irradiation with a handheld 365 nm UV lamp. No dependence on the concentration of **1** was noted at the concentration levels used in the photophysical studies, which makes it unlikely that the observed variations in the absorption and emission spectra are due to the presence of **1** as different oligomeric forms.

Model compound **3**, whose energetically lowest‐lying excited state also has partial CT character, shows the same qualitative behavior while lacking the peak at higher energy. Figures S40 to S47 of the Supporting Information provide compilations of emission spectra of **1** and **3** recorded in different solvents at *λ*_exc_ = 340 and 450 nm as well as the corresponding CIE plots. Fluorescence QYs likewise vary with excitation wavelength and solvent. Values for 1 range from 7.4 % to 31.0 %, with the highest value obtained in toluene at *λ*_exc_ = 320 nm. Those for model compound **2** are similarly high, while those of **3** are lower (see Table 3, Figure S53, and Table S1 of the Supporting Information).

**Table 3:** PL data of tetrad **1** and model complexes **2** and 3. The data are given as *λ*_exc_ [nm]: *λ*_em_ [nm] (*τ*_em_), emission color, and *Φ*_em_.

r. t.	77 K
1	
**CH_2_Cl_2_** 320: **∙** 428 (*τ*_em_ = 3.39, 0.60 ns) 320: **∙** 574 (*τ*_em_ = 6.36, 1.99 ns), steelblue, 11.8 % 380: **∙** 432 (‐) 380: **∙** 574 (‐), steelblue, 11.2 % 390: **∙** 441 (‐) 390: **∙** 564 (‐) 510: **∙** 608 (*τ*_em_ = 4.80, 1.43 ns), dark orange	**CH_2_Cl_2_** 320: **∙** 608 (‐), dark orange
	Toluene 320: **∙** 562 (‐) 320: **∙** 592 (‐), gold
	Toluene: CHCl_3_ (1:1) 320: **∙** 562 (‐), goldenrod
	**2‐Me‐THF** 320: **∙** 390 (‐) 320: **∙** 410 (‐) 320: **∙** 430 (‐) 320: **∙** 470 (‐) 320: **∙** 500 (‐) 320: **∙** 550 (‐) 320: **∙** 600 (‐), light green, 57.3% 440: **∙** 390 (*τ*_em_ = 5.76 ns, 2.44 ns) 440: **∙** 410 (*τ*_em_ = 5.76 ns, 2.44 ns) 440: **∙** 440 (*τ*_em_ = 5.76 ns, 2.44 ns) 320: **∙** 470 (*τ*_em_ = 5.55, 1.93 ns) 320: **∙** 500 (*τ*_em_ = 6.67, 2.09 ns, 2.5 s) 320: **∙** 550 (‐) 320: **∙** 650 (*τ*_em_ = 5.86, 1.88 ns, 0.38 s), 63.4%
**Toluene** 340: **∙** 415 (‐) 340: **∙** 490 (‐), dark cyan, 31.0 % 450: **∙** 500 (‐), lime green, 28.1%	
**THF** 320: **∙** 412 (‐) 340: **∙** 530 (‐), dark seagreen, 23.2% 440: **∙** 538 (‐), yellow‐green, 28.2%	
**Chlorobenzene** 340: **∙** 420 (‐) 340: **∙** 540 (‐), bluish‐white 450: **∙** 552 (‐), golden	
**Dimethylformamide** 340: **∙** 420 (‐), 570 (‐), cornflower blue 450: **∙** 620 (‐), dark orange	

2	
**CH_2_Cl_2_** 380: **∙** 435 (3.67 ns), royal blue, 30.7%	**2‐Me‐THF** 320: **∙** 410 (*τ*_em_ = 1.73, 5.19 ns) 320: **∙** 437 320: **∙** 467 320: **∙** 500 (*τ*_em_ = 1.90, 6.44 ns, 2.85 s) 320: **∙** 531, cadet blue, 47.7%

3	
**CH_2_Cl_2_** 450: **∙** 575 (*τ*_em_ = 4.11, 1.52 ns), goldenrod, 450: **∙** 4.8% at *λ*_exc _= 440 nm	**2‐Me‐THF** 440: **∙** 532 nm (*τ*_em_ = 3.15, 8.80 ns, 0.48 s), yellow‐green, 430: **∙**37.1%
THF 450: **∙** 540 (*τ*_em_ = 5.93, 2.53 ns), yellow‐green	
2‐CHO_3_‐^Et^TAT	
**CH_2_Cl_2_** 420: **∙** 502 (*τ*_em_ = 4.29 ns), seagreen	–

**Figure 8:**
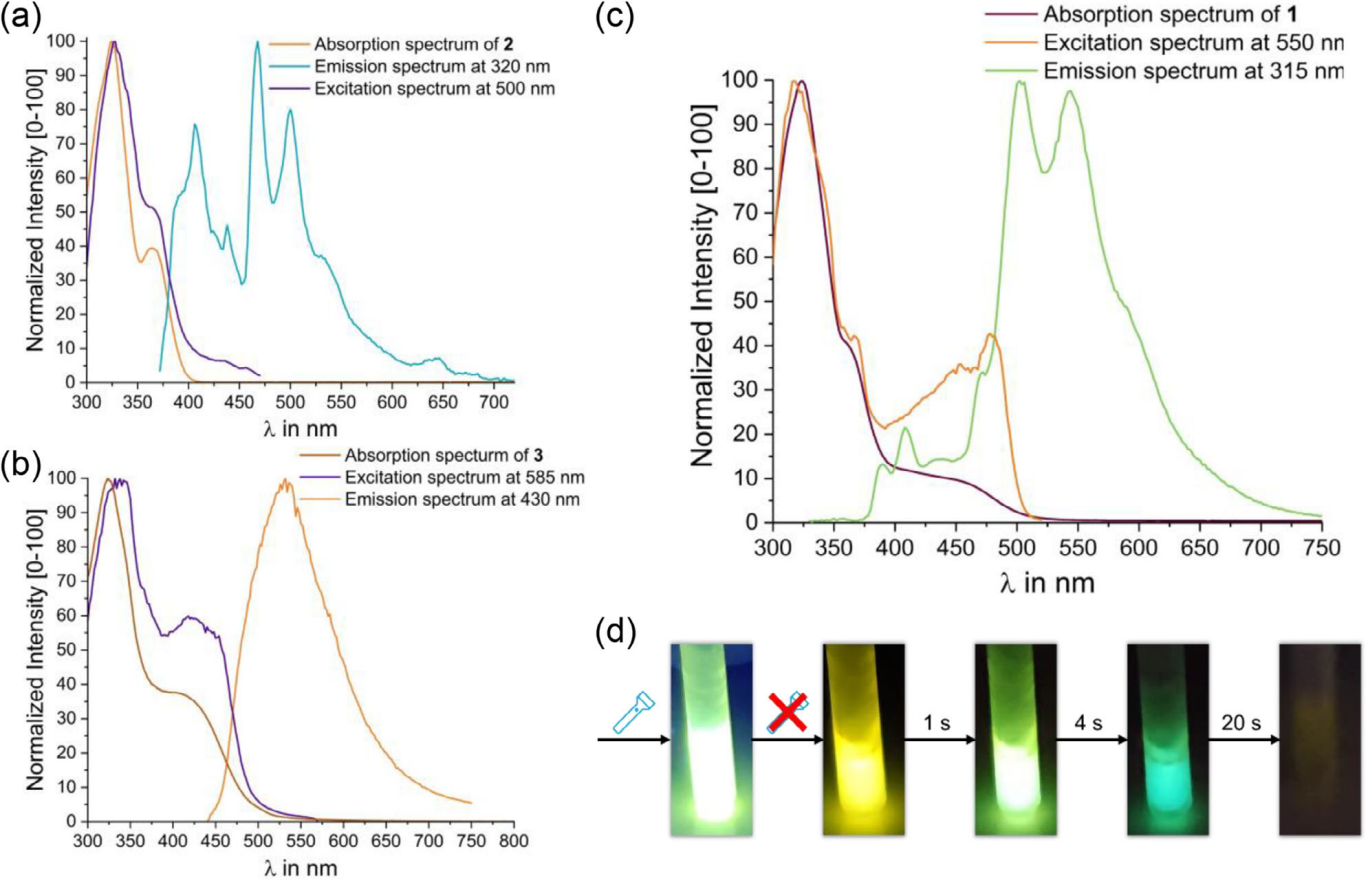
a) Normalized absorption, emission (x = 0.19, y = 0.28, z = 0.53; R = 0, G = 163, B = 190; #00A3BE, cadet blue), and excitation spectra of compound **2** recorded at the indicated wavelengths. The absorption spectrum was measured at r. t. in CH_2_Cl_2_, and PL spectra in 2‐methyl‐THF at 77 K. b) Normalized absorption, emission, and excitation spectra of compound **3** recorded at the indicated wavelengths. The absorption spectrum was measured at r. t. in CH_2_Cl_2_, while PL spectra were obtained in 2‐methyl‐THF at 77 K. c) Normalized absorption, emission (x = 0.33, y = 0.49, z = 0.18; R = 129, G = 205, B = 92; #82CA58, light green), and excitation spectra of **1** recorded at the respective wavelengths as given in the legend. The absorption spectrum was measured in CH_2_Cl_2_ at r. t., while the PL spectra were obtained in 2‐methyl‐THF at 77 K. d) Photographs demonstrating the temporal evolution of the visual color impression of the light emitted from **1** (T = 77 K, 2‐methyl‐THF) under constant irradiation with a 365 nm handheld lamp or at different delay times after the light source was switched off. Complementary Video 3 is provided as Supporting Material.

Photoluminescence measurements at 77 K in 2‐methyl‐THF add another intriguing facet to the rich photophysical properties of TATs **1**–**3**. Under these conditions, solutions of compound **2**, which represents the peripherally appended TAT entities of **1**, show dual blue fluorescence and cyan phosphorescence emission, the latter peaking at 467 nm (see Figure 8a). The phosphorescence lifetime *τ*_Ph_ of ca.  2.85 s leads to a clearly discernible afterglow as demonstrated by Video 1, which is provided as additional material. The combined QY upon excitation at 320 nm is 48 %. In the 2‐Me‐THF glass at 77 K, compound **3**, which models the inner TAT moiety of **1**, exhibits a single, broad phosphorescence emission at 532 nm with an overall QY of 37 % and *τ*_Ph _= 483 ms (see Figure 8b and complementary Video 2).

Tetrad **1** again behaves as a molecular blend of model compounds **2** and **3** and exhibits dual phosphorescence emissions at 77 K, each associated with its own, specific lifetime of 375 ms for the yellow and of 2.50 s for the green emission (see Figure 8c, d). The combined QY maximizes at 63 % for *λ*_exc_ = 440 nm. The continuous change of emission color with time is clearly visible by naked eye. Figure 8d shows photographs recorded under irradiation at 365 nm and at variable delays after the light source was switched off. Emission spectra recorded at different wavelengths suggest that the yellow phosphorescence is specifically triggered by irradiation into the low‐energy vis band within the absorption envelope of **1**, which is specific to the inner TAT moiety (see Figure S50 of the Supporting Information). The phosphorescence emissions are complemented by the blue and orange fluorescence emissions of the two different kinds of TATs present within **1** that both show biexponential decays with associated lifetimes of 2.44 and 5.69 ns and of 1.90 and 6.44 ns. Just like in fluid solution, the exact locations of the emission peaks at 77 K also vary with solvent. Thus, the phosphorescence emission from the inner TAT entity is shifted to 608 nm in a matrix of frozen CH_2_Cl_2_, leading to an orange afterglow (see Figures S48, S49, and Videos 3 and 4 of the Supporting Information). We note that others have already reported TADF emissions of TATs or TAT donor‐acceptor dyads and triads.^[^ ^75^, ^76^, ^77^, ^78^, ^79^, ^80^ ^]^

### 2.3 OLED Devices with **1** in the Emissive Layer

**Figure 9:**
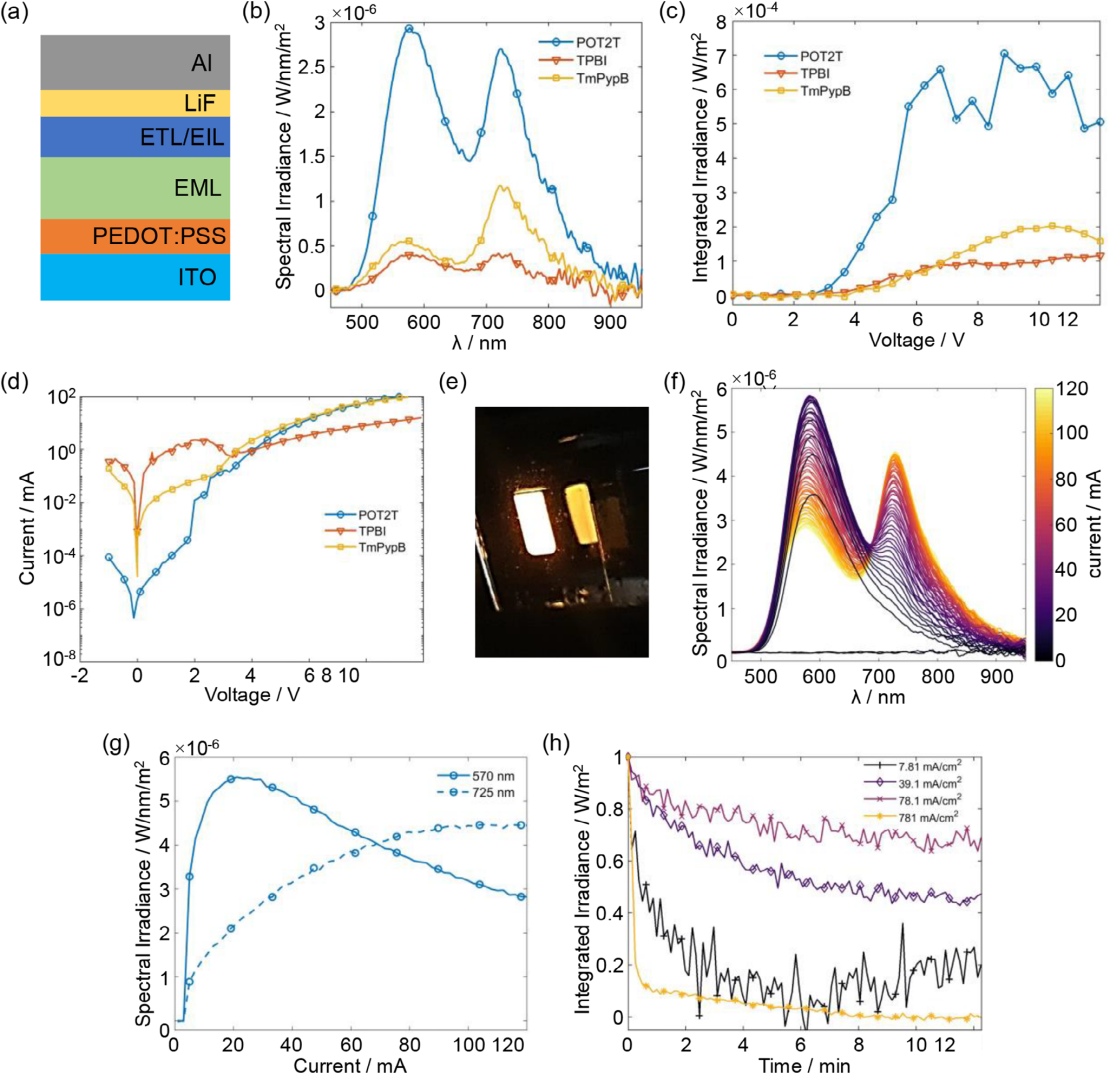
a) Schematic design of our OLEDs. b) Spectral profiles of OLEDs at an applied voltage of 12 V. c) Integrated radiance as a function of the applied voltage. d) *J*‐*V* curves for OLEDs with the different ETLs employed in this study. e) Photograph of light emission from our OLEDs. f) Current sweep of the EL spectra. g) Integrated current of a representative OLED employing **1** as the EML spin coated from a THF solution with a concentration of 2 mg/mL and **PO‐T2T** as ETL. CIE coordinates of the emission recorded at a current of 21 mA are x = 0.519 and y = 0.476; RGB R = 249 G = 168 B = 0; color code #F9A800; gold. h) Lifetime of an OLED under an ambient atmosphere at different injection current densities.

**Figure 10:**
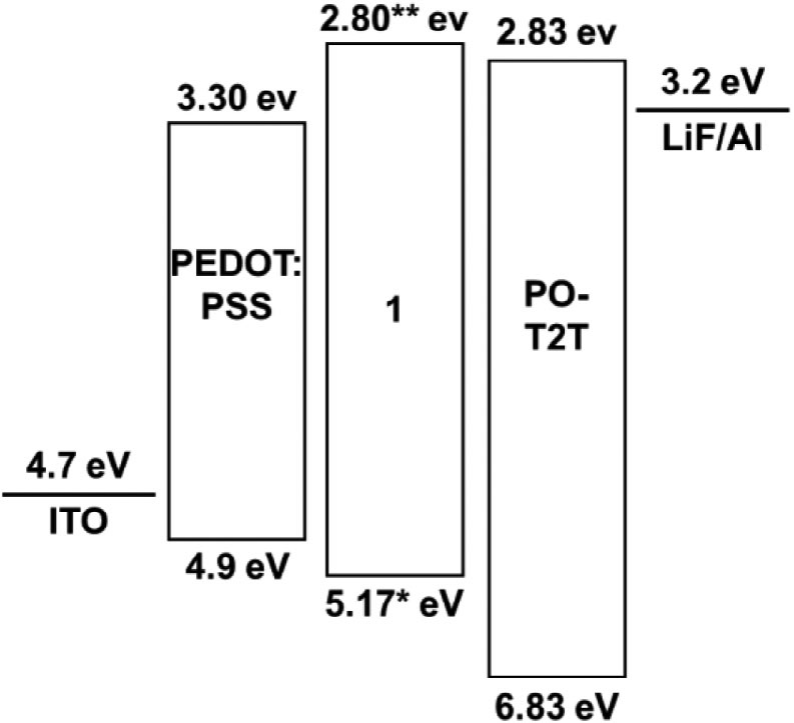
Energy level diagram of the best‐performing OLED. The HOMO level of **1** was calculated using the potential for the first multi‐electron redox process given in Table 1 with calibration against the Cp_2_Fe^0/+^ redox couple (*E*_HOMO_ = ‐ 4.80 eV).^[^ ^85^, ^86^ ^]^ The corresponding LUMO level was calculated using the onset of the electronic absorption of 2.37 eV as obtained from the solid absorption spectrum of films of **1**. The energies of ITO,^[^ ^87^ ^]^ **PEDOT**:**PSS**,^[^ ^82^ ^]^ **PO‐T2T**,^[^ ^81^ ^]^ and LiF/Al^[^ ^88^ ^]^ were taken from the literature.

Its intriguing emissive properties prompted us to fabricate and investigate prototype OLEDs with **1** in the emissive layer. A device architecture glass/ITO/**PEDOT**:**PSS**/EML(**1**)/ETL/LiF/Al as shown in Figure 9a was applied, and different electron transport layers (ETLs) were tested. Fabrication protocols are provided in the Supporting Information. Among the investigated designs, the **PEDOT**:**PSS**/**PO‐T2T** charge carrier pair, in combination with a concentration of 2 mg/mL of **1** in THF for the preparation of the EML, showed better performance than devices with **TPBi** or **TmPyPb** as the ETL (for structures, see Figures S2 and S3 of the Supporting Information). Data for devices prepared by spin‐coating using different concentrations of **1** are shown as Figure S78 of the Supporting Information. So‐optimized OLEDs showed a turn‐on voltage of 3.7 V, as opposed to 7 V for OLEDs with **TPBi** or **TmPyPb** in the ETL, and produced significantly higher light output and optical power densities, as shown in Figure 9b‐d). The superior performances of devices with **PO‐T2T** as the ETL are likely due to more balanced charge carrier injection (electrons and holes) and increased charge carrier mobilities, as shown by the data in Table 4. This also matches with the good energetic alignment of the relevant frontier MOs of the components involved (see Figure 10). From the onset of electronic absorption of films of **1** at 525 nm, which is very similar to that in solution, we calculate a HOMO‐LUMO gap of 2.37 eV, which places the HOMO of **1** at an energy of 5.17 eV, based on the onset of the oxidation at 0.366 V as determined by cyclic voltammetry (vide supra). The estimated LUMO energy of **1** of 2.80 eV aligns well with the LUMO of **PO‐T2T** (2.83 eV),^[^ ^81^ ^]^ while the HOMO energy is well‐matched by that of **PEDOT** (4.9 eV),^[^ ^82^ ^]^ thereby providing efficient charge carrier injection (Figure 10). We note that the turn‐on voltage of 3.7 V is in the typical range of other TAT‐based OLEDs in the literature (Table 5).^[^ ^80^, ^83^, ^84^ ^]^

**Table 4:** Carrier mobility in cm2/Vs of the used HIL/EIL layers in the OLED devices.

HIL/EIL	Charge carrier mobility *μ* [cm^2^/Vs]
**PEDOT:PSS** ^[^ ^89^ ^]^	3⋅10^−4^
**PO‐T2T** ^[^ ^90^ ^]^	∼ 1⋅10^−3^
**TPBi** ^[^ ^91^ ^]^	5.6⋅10^−8^
**TmPyPb** ^[^ ^92^ ^]^	1⋅10^−5^ ‐ 1⋅10^−6^

**Table 5:** Electroluminescence Data of tetrad **1** as EML and the OLED with the mixed EML of model complexes **2** and **3** (3:1).

glass/ITO/PEDOT:PSS/EML[1]/ETL/LiF/Al			glass/ITO/PEDOT:PSS/EML[2 + 3/ 3:1]/ETL/LiF/Al		
*λ*_max_^[^ ^a^ ^]^ [nm]	*F*_max_^[^ ^b^ ^]^ [µW⋅nm^−1^⋅m^−2^], I_inj_	*Turn‐On Voltage [V]*	*λ*_max_^[^ ^a^ ^]^ [nm]	*F*_max_^[^ ^b^ ^]^ [µW⋅nm^−1^⋅m^−2^]	*Turn‐On Voltage [V]*
**580**	5.87 at 21 mA	3.7	**580**	3.10 at 54 mA	4.2 V
**725**	4.66 at 120 mA	6.0	–		

*J* ^[^ ^c^ ^]^ [mA/cm^2^]	Relative *F* ^[^ ^b^ ^]^ after 12 min [%]	*J* ^[^ ^c^ ^]^ [mA/cm^2^]	Relative *F* ^[^ ^b^ ^]^ after 12 min [%]
7.81	20	7.81	55
39.1	70	39.1	63
78.1	48	78.1	48
781	4	781	‐

^[a]^ Emission maximum;

^[b]^ Peak spectral irradiance;

^[c]^ Injected current density

Figure 9e provides photographic impressions of the light output from an OLED at an applied voltage of 7 V; a video is provided as accompanying material. Corresponding EL curves and spectral irradiances at the two emission maxima of the representative best‐performing OLED employing the **PEDOT**:**PSS**/**PO‐T2T** design are provided in Figure 9f, g). Somewhat surprisingly, the OLED shows exclusively the emission from the core TAT entity at 580 nm. This may be due to the quenching of the energetically higher‐lying emission from the peripheral TATs in the film or to reabsorption of the emission from the peripheral TAT units by the inner TAT. Spectral irradiance of this emission peaks at 5.87 µW⋅nm^−1^⋅m^−2^ at a current of 21 mA.

At higher driving voltages, a second emission peaking at 725 nm that extends well into the NIR develops. This specific emission has no counterpart in solutions of **1** or **3** and was also not observed for films of **1** (see Figure S75 of the Supporting Information). This feature emerges at an applied voltage of >6 V and gains in intensity at higher driving voltages and current densities. The peak spectral irradiance of this emission is 4.66 µW nm^−1^ m^−2^ at 120 mA. Considering its low energy and the high turn‐on voltages, we tentatively assign this emission to aggregates of emissive units in the film, or possibly to **1^+^** radical cations, e. g., in the form of pimers.

Figure 9h shows how the integrated irradiances of our devices change over time when operated at different injection current densities under ambient conditions. The highest spectral stability was obtained at 78 mA/cm^2^. The EQE roll‐off at *J* = 781 mA/cm^2^ and the resulting EQE drop are likely due to imbalanced charge injection caused by Joule heating. We also studied OLEDs with EMLs consisting of a 3:1 mixture of **2** and **3**, that is, the same molar ratio as in tetrad **1**. The latter devices yielded lower peak irradiances of 3.10 µW⋅nm^−1^⋅m^−2^ at 54 mA. Moreover, they exhibited inferior stability and showed linear performance decay during powering. Corresponding data is shown as Figure S79 to S84 of the Supporting Information.

## 3 Summary and Conclusions

We have presented the TAT tetrad **1**, where three peripheral TAT entities are connected to an internal TAT via 2‐propyne‐1‐ol linkers, as well as two new mono‐TAT derivatives, **2** and **3**, that model the internal and the peripheral TAT moieties of **1**. As every linker features a chiral C atom, trisubstituted TATs **1** and **3** come as mixtures of four enantiomers. Mutual insulation of the emissive TAT entities in **1** gives rise to two distinct fluorescence emissions whose relative intensities depend on the excitation wavelength and the solvent, the latter being due to excitations with partial CT between the different TAT units. This renders tetrad **1** a one‐component emitter whose visual emission can be tuned from blue to orange, including near‐white emission, as the two emission bands are nearly complementary in color. At 77 K, both types of emissive subunits additionally provide long‐lived phosphorescence with lifetimes of 0.4 and 2.5 s, respectively, leading to a continuously changing afterglow, and with combined QYs of up to 63 %. The continuous color change of the cryogenic emission makes **1** a viable candidate for anticounterfeiting applications.

OLEDs fabricated with **1** in the emissive layer provided the best balance between hole and electron injection and the lowest leakage ratio for a design glass/ITO/**PEDOT**:**PSS**/**1**/**PO‐T2T**/LiF/Al, with a turn‐on voltage of 3.7 V for yellow light emission peaking at 580 nm. The highest irradiance of 5.87 µW⋅nm^−1^⋅m^−2^ was observed for driving voltages of 6.7 V (21 mA), which ensures low‐voltage operation of the OLED. At higher driving voltages of >6 V, a second emission peaking at 725 nm was observed, with a gradual change of the emission color to goldenrod. Stability tests of nonencapsulated devices showed that 70 % or 48 % of the initial irradiance remained after 12 minutes of operation under ambient conditions, at current densities of 39.1 or 78.1 mA/cm^2^. Our studies thereby indicate that the electronically isolating propynol linker allows for shaping arrays of chemically different, emissive entities. This offers exciting possibilities for the design of single‐molecule emitters with tunable multicolor emission and their application in OLEDs.

## Supporting Information

Additional references cited within the Supporting Information.^[^ ^93^, ^94^, ^95^, ^96^, ^97^, ^98^, ^99^, ^100^, ^101^, ^102^, ^103^, ^104^, ^105^, ^106^, ^107^ ^]^

## Conflict of Interest

There are no conflicts to declare.

## Supporting information



Supporting Information

Supporting Information

Supporting Information

Supporting Information

Supporting Information

Supporting Information

Supporting Information

## Data Availability

The data that support the findings of this study are available in the supplementary material of this article.
