# Supplementary material for: A Bichromophoric Triazatruxene Tetrad as a Highly Tunable Multicolor Emitter and Its Application in OLEDs
Source: Chemistry. 2025 Aug 30;31(56):e01992. doi: 10.1002/chem.202501992 (PMC12510140; doi:10.1002/chem.202501992)
Supplement: Supplementary file 1 — Supporting Information [file CHEM-31-e01992-s005.pdf]

A Bichromophoric Triazatruxene Tetrad as a Highly Tunable Multicolor Emitter and its Application in OLEDs

Lars Vogelsang, Muhammad Irfan Haider, Kai Vogelsang, Tobias Seewald, Azhar Fakharuddin, Niklas Bauch, Gabriel Maier, Katharina L. Deuter, Tobias Birk, Mikhail Fonin, Lukas Schmidt-Mende*, and Rainer F. Winter**

Supporting Information

List of contents

Materials and methods 2
- NMR spectroscopy 2
- Electrochemical investigations 2
- UV/vis/NIR spectroscopy and spectroelectrochemistry 2
- DFT calculations 2
- OLED fabrication 3
- OLED characterization and photoluminescence measurements 4
- Scanning tunnelling microscopy 4

Synthetic details and characterization 5
- NMR spectroscopy and mass spectrometry 7
- Voltammetric measurements 14
- UV/vis/NIR and IR spectroscopy and spectroelectrochemistry 17
- Photoluminescence measurements 25
- Scanning tunneling microscopy experiments 47
- OLED performances 48
- Quantum chemistry 52

References 61

Materials and methods

NMR spectroscopy

NMR measurements were conducted on a *Bruker* Avance III 400 (^{1}H-NMR: 400 MHz, ^{13}C-NMR: 101 MHz), a *Bruker* Avance Neo 800 (^{1}H-NMR: 800 MHz, ^{13}C-NMR: 202 MHz), a *Bruker* Avance III 600 (^{1}H-NMR: 600 MHz, ^{13}C-NMR: 152 MHz) or on a *Jeol* JNM-ECZR (^{1}H-NMR: 500 MHz), ^{13}C-NMR: 121 MHz) spectrometer. All spectra were measured in deuterated solvents and referenced to the residual protons in the deuterated solvent for ^{1}H-NMR spectra or their ^{13}C-signal for ^{13}C{^{1}H}-NMR spectra.

Electrochemical investigations

Cyclic voltammograms were measured under an argon atmosphere in the CH_2Cl_2/NBu_4^+ $[BAr^{F24}]^-$ ($[BAr^{F24}]^-$ = $[B\{C_6H_3(CF_3)_2\text{-}3,5\}_4]^-$) electrolyte. Na^+ $[BAr^{F24}]^-$ was synthesized from 3,5-bis(trifluoromethyl)bromobenzene according to published procedures.[93] Subsequent exchange of the Na^+ cation for NBu_4^+ was conducted according to literature-known procedures.[94] Voltammetric measurements were performed with a computer-controlled *BASi* potentiostat. A custom-made cylindrical, vacuum-tight single-compartment cell with a platinum working electrode was utilized. A spiral-shape platinum wire was used as counter electrode and a silver wire as the (pseudo)reference electrode. These electrodes are sealed into glass capillaries and fixated to sidearms of the cell using Quickfit screws. The working electrode was polished with diamond pastes of 1 μm and 0.25 μm grain size prior to measurements. The working electrode was inserted into the top port of the cell through a Quickfit screw. Referencing was done using either decamethylferrocene (Cp^*_2Fe, $E_{1/2}$ = –550 mV vs $Cp_2Fe^{0/+}$) or cobaltocenium hexafluorophosphate ($[Cp_2Co]^+$ $[PF_6]^-$, $E_{1/2}$ = –1330 mV vs $Cp_2Fe^{0/+}$) as internal reference. Potentials are provided relative to the ferrocene/ferrocenium ($Cp_2Fe^{0/+}$ (further on denoted as FcH/FcH^+) redox couple.

UV/vis/NIR spectroscopy and spectroelectrochemistry

FT-IR spectra were recorded on a *Bruker* Tensor III setup in a range between 1000 cm^{-1} to 11500 cm^{-1}. UV/vis/NIR spectra were measured with a *TIDAS* fiberoptic diode array spectrometer using MCS UV/vis and PGS NIR instruments from *J&M*. Extinction coefficients were measured in *Hellma* quartz cuvettes of 0.1 cm thickness. Spectroelectrochemical measurements were conducted in an OTTLE (optically transparent thin-layer electrochemical) cell according to the design of Hartl et al.[95] The cell is custom-built with CaF_2 windows, Pt-mesh working and counter electrodes and a Ag/AgCl pseudo-reference electrode. The measurements were conducted in a dry and degassed 1,2-$C_2H_4Cl_2/NBu_4^+$ $[BAr^{F24}]^-$ electrolytes. Potentials were applied using a *Wenking Pos 2* potentiostat by *Intelligent Controls GmbH*. IR spectra in transmission were recorded in a KBr-pellet using the same IR spectrometer using a MIR-lamp. The pellets were made by doping pre dried potassium bromide powder with the compound and subsequent pressing with 10 tons of pressure. ATR FT-IR spectra were measured using dry powders on a *Bruker* Alpha ATR-spectrometer equipped with a DTGS detector.

DFT calculations

Quantum chemical calculations on the compounds were executed using GAUSSIAN 16 program packages.[96] Alkyne functionalities were supported with dummy atoms to avoid angles of 180°. Electronic transitions were rendered by the time-dependent DFT approach (TD-DFT). For all atoms, triple-ζ basis sets (6-31G(d))[97] were applied for geometrical optimization and construction of the molecular orbitals. PBE1PBE functionals were used for all calculations.[98] For energetic considerations, the Grimme's dispersion with Becke-Johnson damping was added to account for dispersion interactions in the dimer and in the monomer of **1** (prompt: EmpiricalDispersion=GD3BJ).[99] Solvent effects were accounted for by using the polarizable conductor continuum model (PCCM).[100] Molecular orbitals are pictured in blue and white for positive and negative signs of the wave functions. The isovalues were set to 0.02 for molecular orbitals, and to 0.001 for spin orbitals.

OLED fabrication

ITO substrates (*Lumtec*, 14x14) were etched/patterned (Zn powder and conc. HCl) and passed through sequential cleaning in a sonication bath with a neutral detergent, deionized water, acetone and isopropanol for 15 minutes each. Subsequently, the substrates were dried with N_2 and further cleaned with an UV ozone cleaner (*Ossila*) for 25 minutes before the HTL deposition. A solution of PEDOT:PSS was filtered through a 0.45 µm PVDF membrane and spin coated at 6000 rpm for 60 seconds. This was followed by annealing at 130 °C for 10 minutes. Without delay, the films were transferred into the glove box. The compounds used for the emissive layer (EML) were dissolved in dry THF in concentrations of 1, 2, 5 and 10 mg/mL for spin coating and transferred into the glovebox. Subsequently, the films were spin coated with the EML at 2000 rpm for 30 seconds and annealed at 80 °C for 10 minutes. The substrates were then transferred to a thermal evaporator fitted in the glovebox to complete the device fabrication at a pressure of less than $6{\cdot}10^{-6}$ mbar. 40 nm of TmPyPB, TPBi and PO-T2T each (all from *Lumtec*) were thermally evaporated as electron transport layer (ETL) at a rate of 0.2 Å/s, followed by vapor deposition of 1.5 nm of LiF. Finally, 100 nm of Al was evaporated (2 Å/s) by using a shadow mask of 0.13 cm^2 to define the active area of the devices. The construction of the device is schematically shown in Figure S1, while the structures of the indicated compounds are compiled in Figures S2 and S3.

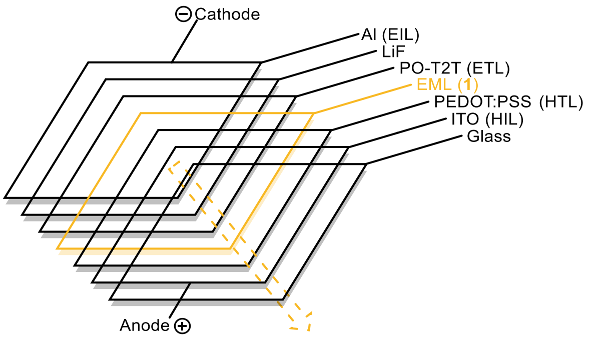

Figure S1. Schematic depiction of the design of the best-performing OLED.

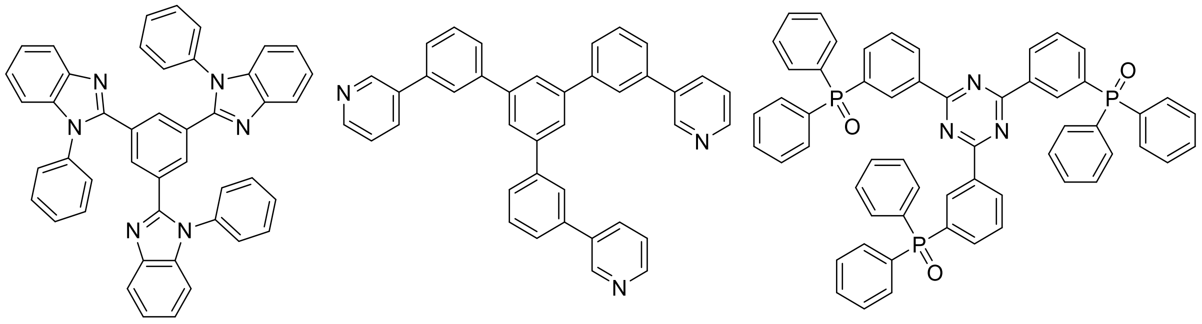

TPBi

2,2′,2"-(1,3,5-Benzenetriyl)-tris(1-phenyl-1-H-benzimidazole))

TmPyPb

1,3,5-Tri(*m*-pyridin-3-ylphenyl) benzene

PO-T2T

(1,3,5-Triazine-2,4,6-triyl)-tris(benzene-3,1-diyl)-tris(diphenylphosphine oxide)

Figure S2. Chemical structures and IUPAC names of the compounds used to form the ETLs.

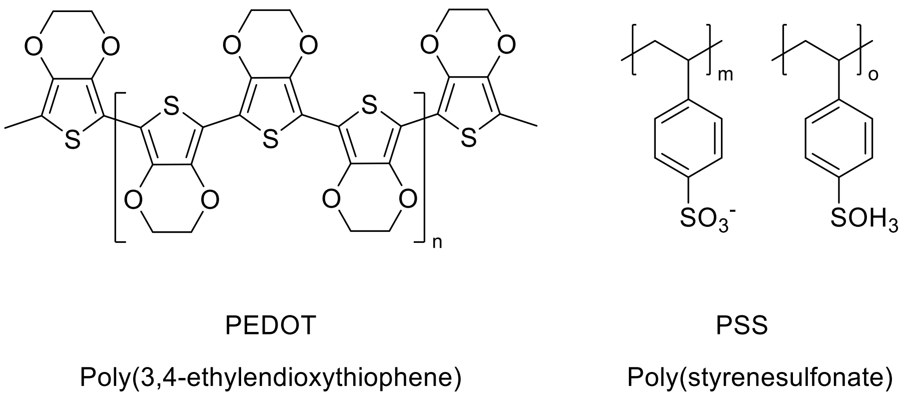

Figure S3. Chemical structures and IUPAC names of the compounds used for the HTL composite polymer.

OLED characterization and photoluminescence measurements

A PHELOS angular luminescence spectrometer (*FLUXiM*) was used to record the *J*-*V* curves, electroluminescence spectra, voltage sweeps, current sweeps and lifetimes of the OLEDs. (Excitation intensity-dependent) photo luminescence measurements were performed by using a FluoTime 300 fluorimeter by *PicoQuant*. The used excitation intensities for the intensity-dependent measurement of a quartz-plated film of compound **1** are given in Figure S72. Spectra at room temperature (r. t.) were recorded on either quartz- or glass-plated films obtained by spin coating, or from solutions in the indicated solvent in *Hellma* cuvettes (d = 1 cm). Low-temperature work at 77 K employed frozen, glassy solutions in 2-methyl-THF in Boroeco 5-7 NMR tubes (d = 0.5 cm) purchased from *Deutero* GmbH. Quantum yields were determined with a C-11347-01 absolute PL quantum yield spectrometer by *Hamamatsu photonics* for solutions in the respective solvents in 0.6 cm quartz tubes for 77 K measurements, or in modified *Hellma* cuvettes (d = 1cm) for studies at r. t., using the software U6039-05 by *Hamamatsu photonics*.

Scanning tunnelling microscopy

The Ag(111) crystal (Surface Preparation Laboratory B. V.), which was used as the substrate, was cleaned by repeated cycles of Ar^+ sputtering (2 kV) and subsequent annealing to 600 °C. Molecular materials were deposited *in situ via* electrospray deposition (ESD) form freshly prepared solutions in a mixture of dichloromethane and methanol. The Ag(111) crystal was kept at room temperature during the deposition. The used ESD setup is described elsewhere.[101] All STM measurements were performed in a two-chamber ultra-high vacuum (UHV) system (base pressure $5 \cdot 10^{-11}$ mbar), equipped with an *Omicron* Cryogenic-STM. Data acquisition was performed at 3 - 6 K. For all measurements, grinded and polished PtIr tips (*Nanoscore GmbH*) were used. For the normalized differential conductance ($\mathrm{d}I/\mathrm{d}U)/(I/U)$ curves, $I(U)$ spectra were obtained, numerically differentiated and normalized to the I/U signal.

Synthetic details and characterization

All syntheses were carried out under nitrogen atmosphere using common Schlenk techniques. Solvents were dried over appropriate drying agents, deoxygenated by purging with dinitrogen, or by three freeze-pump-thaw cycles, and stored under nitrogen atmosphere. All starting materials either were purchased from commercial suppliers and used without further purification or prepared from literature-known procedures.

2-Br$_3$-^{Et}TAT[66, 102]**, 2-CHO$_3$-^{Et}TAT**[64] and **2-A$_1$-^{Et}TAT**[66] were prepared according or similar to known literature procedures.

2,2',2''-Tri(formyl)-*N*,*N*',*N*''-triethyltriazatruxene **(2-CHO$_3$-^{Et}TAT)**[64]

522 mg (0.78 mmol, 1.0 equiv.) of **2-Br$_3$-^{Et}TAT** were dissolved in 25 mL of dry THF and cooled to – 78 °C. Subsequently, 1.41 mL (3.53 mmol, 4.5 equiv.) of a 2.5 M solution of ^{n}BuLi in ^{n}hexane were dropwisely added. The reaction mixture was stirred for 90 minutes. 0.5 mL (5.09 mmol, 6.5 equiv.) of dimethylformamide were then added to the reaction mixture, and the reaction was left stirring for 3 h after reaching r. t. After this time, 6 mL of HCl_{aq} (3 M) were added. The mixture was extracted with CH_2Cl_2 (3×200 mL). The combined organic phases were dried over Na_2SO_4 and the solvents were removed under reduced pressure. The crude product was dissolved in 5 mL of CH_2Cl_2 and reprecipitated by adding of 40 mL of ^{n}pentane. The product was obtained as a yellow solid in a yield of 75% (300 mg, 0.58 mmol). The product was characterized *via* ^{1}H-NMR spectroscopy, and the observed resonances agreed with those in the literature.[64]

2,2',2''-Tris-(*N,N',N''*-triethyltriazatruxene-2-propyne-3-yl)-*N*,*N*',*N*''-triethyltriazatruxene (**1**)

155 mg (0.34 mmol, 3.4 equiv.) of **2-A$_1$-^{Et}TAT** were dissolved in 15 mL of dry THF and cooled to – 78 °C. Subsequently, 0.14 mL (0.35 mmol, 3.6 equiv.) of a 2.5 M solution of ^{n}BuLi in ^{n}hexane were added dropwise. The reaction mixture was stirred for 90 minutes. A solution of 50 mg (0.10 mmol, 1.0 equiv.) of **2-CHO$_3$-^{Et}TAT** in 7 mL of dry THF was then added. The reaction mixture was allowed to reach r. t. and was stirred overnight. 50 mL of a mixture of CH_2Cl_2 and MeOH (10:1, vol/vol) were added. The solution was washed with H_2O (2×50 mL) and the aqueous phase was extracted with a 10:1 mixture (vol/vol) of CH_2Cl_2 and MeOH (3×50 mL). The combined organic phases were dried over $MgSO_4$ and the solvents were removed under reduced pressure. The red crude product was dissolved in 3 mL of CH_2Cl_2 and reprecipitated by the addition of 40 mL of ^{n}pentane. The solid was washed with dry diethyl ether (4×40 mL). The product was isolated as red microcrystalline powder by dissolving the solid material in 5 mL of CH_2Cl_2, filtering, and slowly evaporating the solvent. The yield was 93% (170 mg, 0.09 mmol).

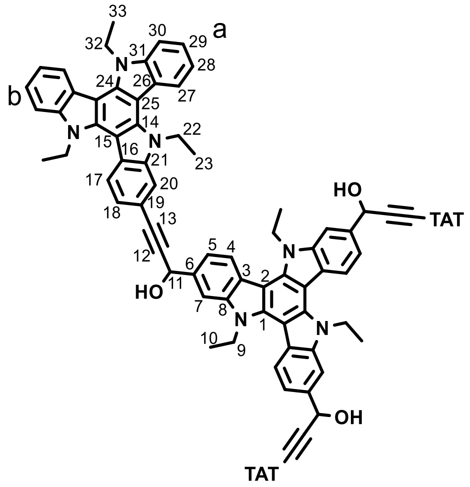

^{1}H-NMR (400 MHz, CD_2Cl_2, 300 K) δ [ppm] = 8.39 – 8.16 (m, 12H, H-4, H20, H-$29_{a,b}$), 8.04 –7.88 (m, 3H, H-5), 7.92 (s, 3H, H-22), 7.74 – 7.56 (m, 9H, H-7, H-$30_{a,b}$), 7.52 (d, *J* = 8.2 Hz, 3H, H-19), 7.48 – 7.37 (m, 6H, H-19), 7.42 – 7.36 (m, 6H, H-$32_{a,b}$), 7.37 – 7.32 (m, 6H, H-$31_{a,b}$), 6.17 – 5.95 (m, 3H, H-11), 5.13 – 4.63 (m, 12H, H-9,H-24, H-$34_{a,b}$), 1.73 – 1.40 (m, 18H, H-10, H-25, H-$35_{a,b}$).

^{13}C-NMR (101 MHz, CD_2Cl_2, 300 K) δ [ppm] = 141.1 (m, C-8, C-$33_{a,b}$), 140.7 (s, C-23), 139.7 (s, C-16), 139.3 (s, C-$26_{a,b}$), 138.6 (s, C-1), 136.6 (s, C-6), 123.6 (s, C-3, C-15), 124.1 (m, C-18, C-21), 123.8 (s, C-20), 123.6 (m, C-3, C-28), 123.4 (s, C-32), 121.8 (m, C-4,C-19, C-$29_{a,b}$), 120.4 (s, C-$31_{a,b}$), 119.2 (m, C-7), 116.4 (s, C-18), 113.9 (s, C-22), 110.8 (s, C-$30_{a,b}$), 108.9 (m, C-5), 103.8 (s, C-17), 103.4 (m, C-2, C-$27_{a,b}$), 89.4 (m, C-14), 88.1 (m, C-15), 66.1 (m, C-11), 42.1 (s, C-9, C-24, C-$34_{a,b}$), 15.9 (m, C-10), 15.7 (m, C-25, C-$35_{a,b}$).

ESI-MS (CH_2Cl_2): 936.43(06) m/z (**1**$^{2+}$, calculated: 936.43(28) m/z, intensity = 1.0); 935.92(23) ({**1**-H}$^{2+}$, calculated: 935.92(89) m/z, intensity = 0.223).

2-(4,4’-Bis(trifluoromethyl)-diphenylmethanol)-ethynyl-*N*,*N*’,*N*’’-triethyltriazatruxene (**2**)

90 mg (0.21 mmol, 1.0 equiv.) of **2-A$_1$-^{Et}TAT** were dissolved in 10 mL of dry THF and cooled to – 78 °C. Subsequently, 0.12 mL (0.31 mmol, 1.5 equiv.) of a 2.5 M solution of ^{n}BuLi in ^{n}hexane were added dropwise. The reaction mixture was stirred for 90 minutes. Subsequently, a solution of 113 mg (0.36 mmol, 1.7 equiv.) of 4,4’-bis(trifluoromethyl)benzophenone in 5 mL of dry THF was added dropwise. The reaction mixture was allowed to warm to r. t. and stirred overnight. The contents of the flask were then poured into 200 mL of H_2O. The organic contents were extracted with CH_2Cl_2 (3×50 mL). The combined organic phases were dried over Na_2SO_4 and the solvents were removed under reduced pressure. The crude product was extracted with pentane / diethyl ether solution (6:1, vol:vol) three times and the solvents were removed in vacuo. The solid was purified *via* column chromatography using CH_2Cl_2 : petroleum ether (1:1, vol:vol) as the eluent. The product was isolated as a microcrystalline, orange powder in a yield of 38 % (0.08 mmol, 55 mg).

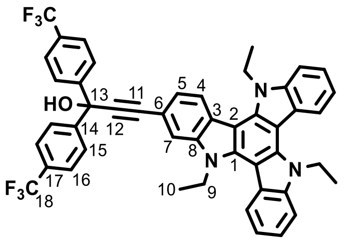

^{1}H-NMR (400 MHz, CD_2Cl_2, 300 K) δ [ppm] = 8.36 (t, *J* = 8.4 Hz, 2H, H-$4_{b,c}$), 8.31 (d, *J* = 8.4 Hz, 1H, H-4), 7.93 (d, *J* = 7.7 Hz, 2H, H-16), 7.80 (d, *J* = 1.5 Hz, 1H, H-7), 7.76-7.60 (m, 6H, H15 + H-$7_{b,c}$), 7.57-7.46 (m, 5H, H5 + H-$6_{b,c}$), 7.39 (t, *J* = 8.2, 7.1, 1.2 Hz, 2H, H-$5_{b,c}$), 5.11-4.96 (m, 6H, H-9, H-$9_{b,c}$), 3.15 (s, 1H, H-OH), 1.70-1.60 (m, 9H, H-10, H-$10_{b,c}$).

^{13}C-NMR (101 MHz, CD_2Cl_2, 300 K) δ [ppm] = 148.5 (d, J = 1.4 Hz, C-14_a), 140.8 (s, C-$8_{b,c}$), 140.2 (s, C-8_a), 139.6, 139.1, 138.4 (each s, C-1, C-$1_{b/c}$), 130.3 (q, $^{2}J_{CF}$ = 32.5 Hz, C-17_a), 125.6 (q, $^{3}J_{CF}$ = 3.7 Hz, C-16_a), 124.3 (s, C-6_a), 124.1 (q, $^{1}J_{CF}$ = 272.2 Hz, C-18_a), 123.6 (s, C-5_a), 123.4 (s, C-$2_{b,c}$), 123.3 (d, J = 18.7 Hz, C-$6_{b,c}$), 121.6, 121.5 (each s, C-$4_{b/c}$), 121.3 (s, C-4_a), 120.1 (vd, *J* = 2.4 Hz, C-$5_{b,c}$), 115.0 (s, C-2_a), 113.5 (s, C-7_a), 110.5 (vd, *J* = 3.9 Hz, C-$7_{b,c}$), 103.6 (s, C-3_a), 103.2 (s, C-$3_{b,c}$), 90.1 (s, C-11_a), 89.3 (s, C-12_a), 74.4 (s, C-13_a), 41.8 (s, C-9_{a-c}), 15.6 (s, C-10_a), 15.5 (vd, *J* = 3.9 Hz, C-$10_{b,c}$).

ESI-MS (CH_2Cl_2): 771.27 m/z (**2**$^{+}$, calculated: 771.27, intensity = 0.4), 772.27 m/z ({**2**+H}$^{+}$, calculated: 772.27, intensity = 1).

2,2’,2‘‘-Tris-((4-methoxyphenyl)-2-propyne-3-yl)-*N*,*N’*,*N’’*-triethyltriazatruxene (**3**)

100 mg (0.76 mmol, 7.8 equiv.) of 4-ethynylanisole were dissolved in 10 mL of dry THF and cooled to –78 °C. Tetramethylethylenediamine 0.78 mL (0.76 mmol, 7.8 equiv.) was added. Subsequently, 0.12 mL (0.31 mmol, 3.3 equiv.) of a 2.5 M solution of ^{n}BuLi in ^{n}hexane were added dropwise. The reaction mixture was stirred for 90 minutes. Subsequently, 50.0 mg (0.09 mmol, 1.0 equiv.) of **2-CHO$_3$-^{Et}TAT**, dissolved in 7 mL of dry THF, were added. The reaction mixture was allowed to reach r. t. and stirred for two days. Afterwards, 100 mL of 1 M $NaOH_{aq}$ were added. The mixture was extracted with CH_2Cl_2 (4×75 mL). The combined organic phases were dried over Na_2SO_4 and the solvents were removed under reduced pressure. The crude product was subsequently washed with ^{n}pentane (3×5 mL) and diethyl ether (4×5 mL). This procedure of dissolving the crude product in CH_2Cl_2, precipitation with ^{n}pentane, pipetting off the supernatant and washing with ^{n}pentane was repeated twice. For further purification, the isolated product was subjected to column chromatography using CH_2Cl_2:NEt_3 (9:1) to yield the product as a reddish solid (70%, 0.06 mmol, 55.0 mg).

^{1}H-NMR (400 MHz, CD_2Cl_2, 300 K) δ [ppm] = 8.54 – 7.35 (m, 17H), 7.04 – 6.75 (m, 6H), 5.98 (s), 6.63 (s), 5.18 – 4.75 (m, 6H), 3.93 – 3.74 (m, 9H), 1.77 – 1.54 (m, 9H).

NMR spectroscopy and mass spectrometry

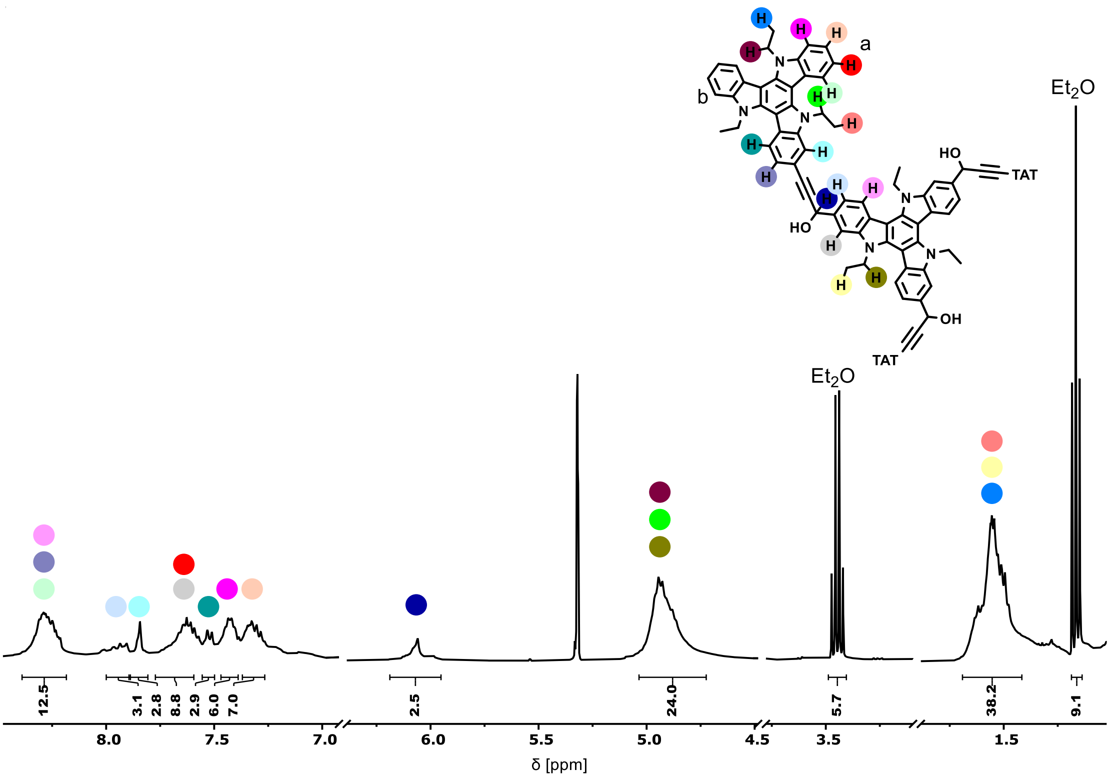

Figure S4. ^{1}H-NMR spectrum (CD_2Cl_2, 400MHz, 300 K) of **1**.

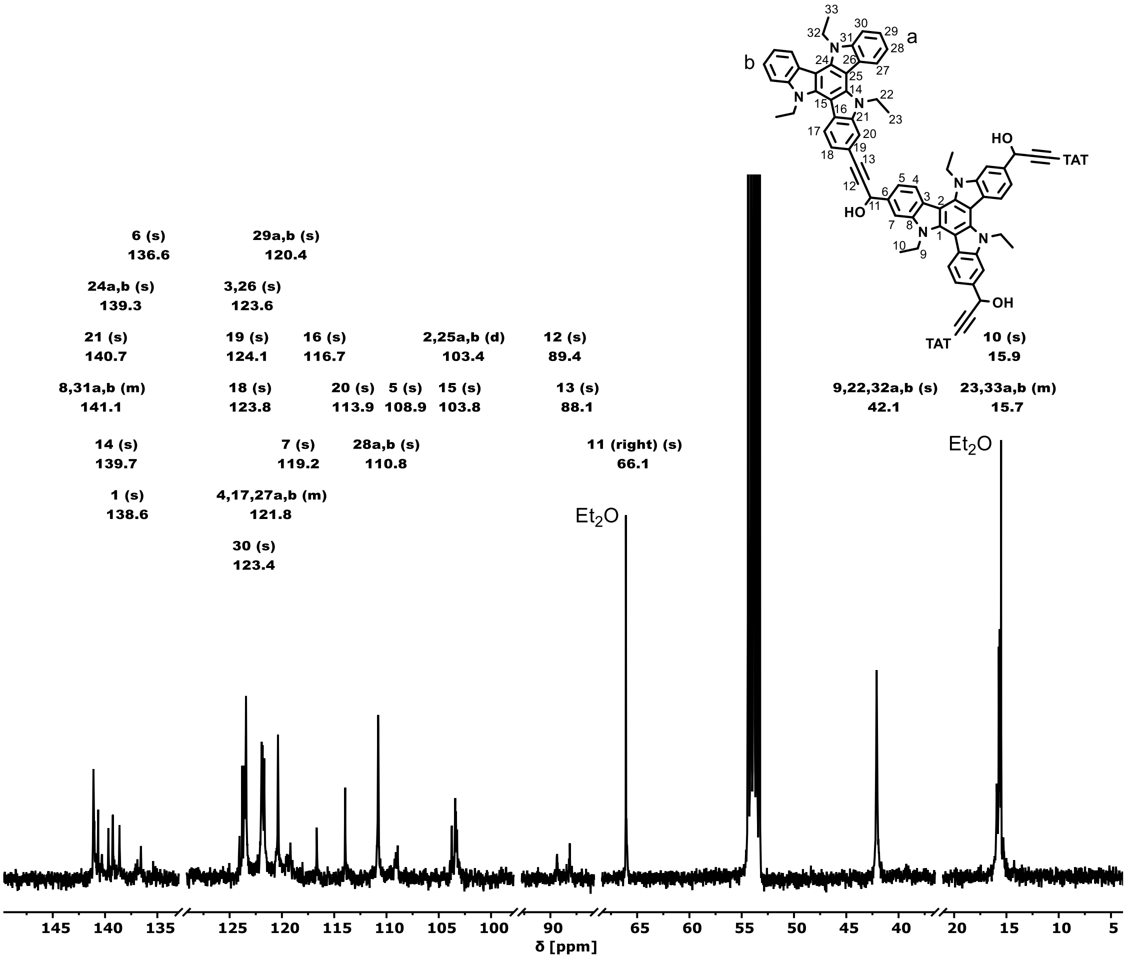

Figure S5. $^{13}C\{^1H\}$-NMR spectrum (CD_2Cl_2, 101 MHz, 300 K) of **1**.

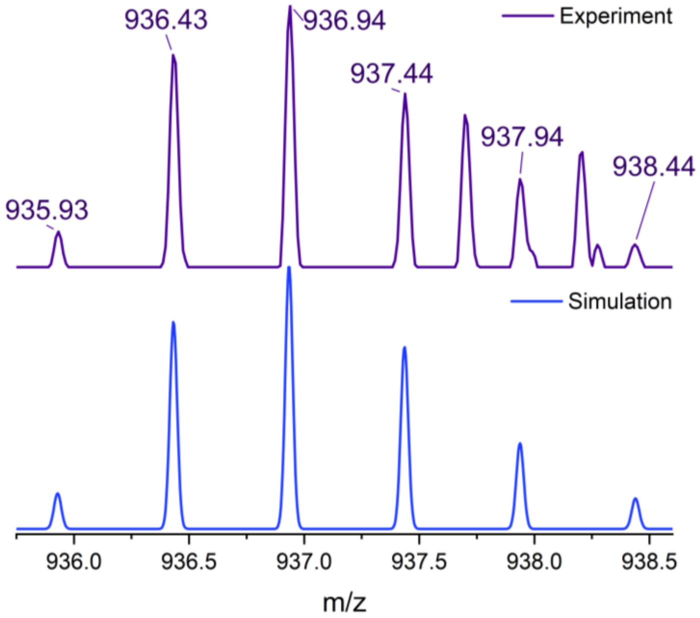

Figure S6. Purple: ESI mass spectrum (CH_2Cl_2) of **1** with selected mass peaks indicated. Blue: Simulated isotopic pattern of $\mathbf{1}^{2+}$ (intensity = 1.0) and $\{\mathbf{1}\text{–H}\}^{2+}$ (intensity = 0.228).

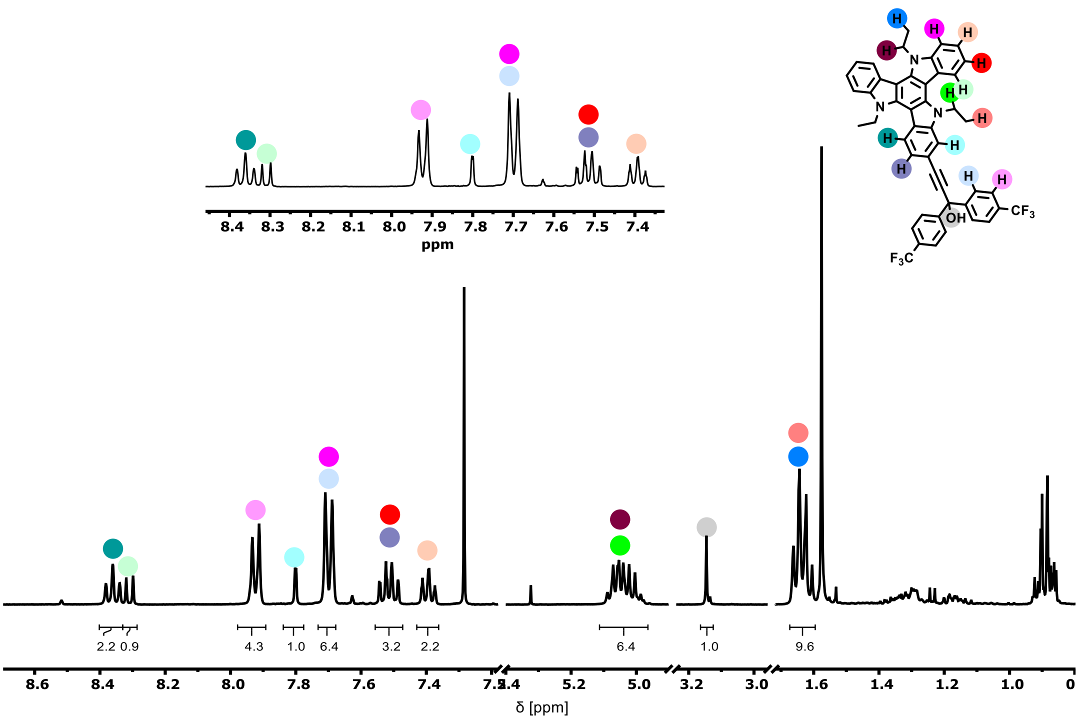

Figure S7. ^{1}H-NMR spectrum ($CDCl_3$, 400 MHz, 300 K) of **2**.

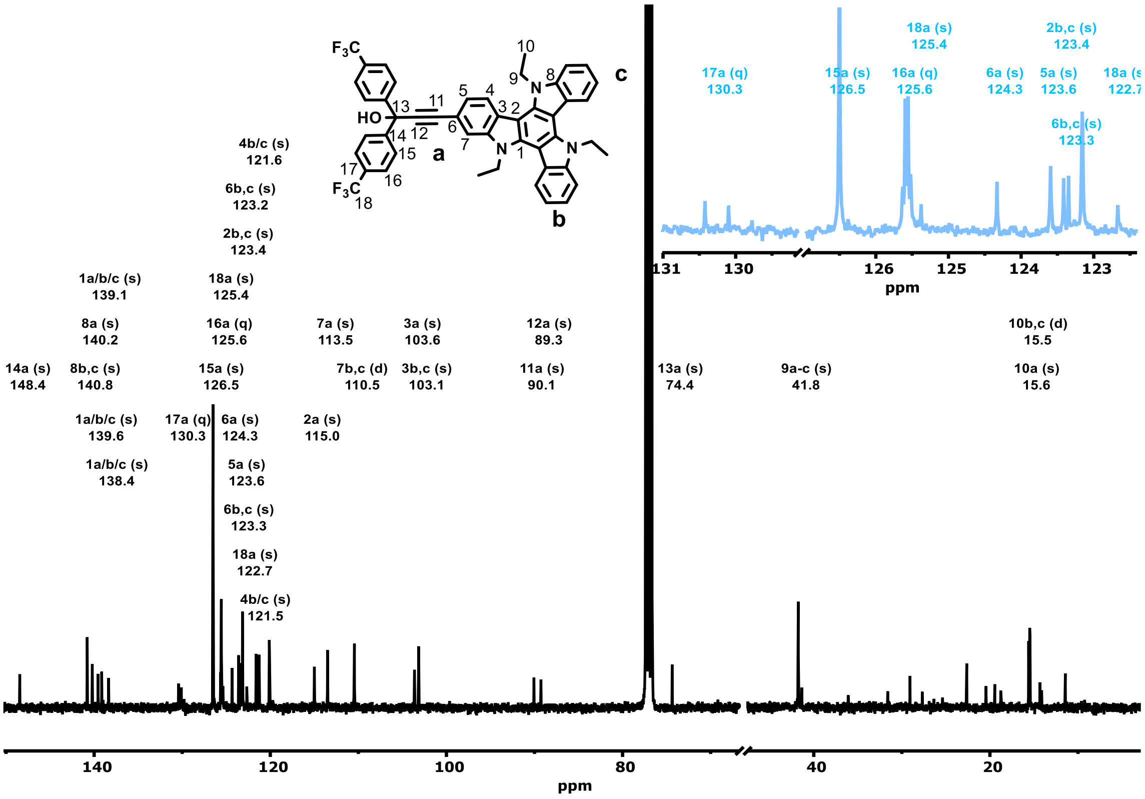

Figure S8. ^{13}C{^{1}H}-NMR spectrum ($CDCl_3$, 101 MHz, 300 K) of **2**.

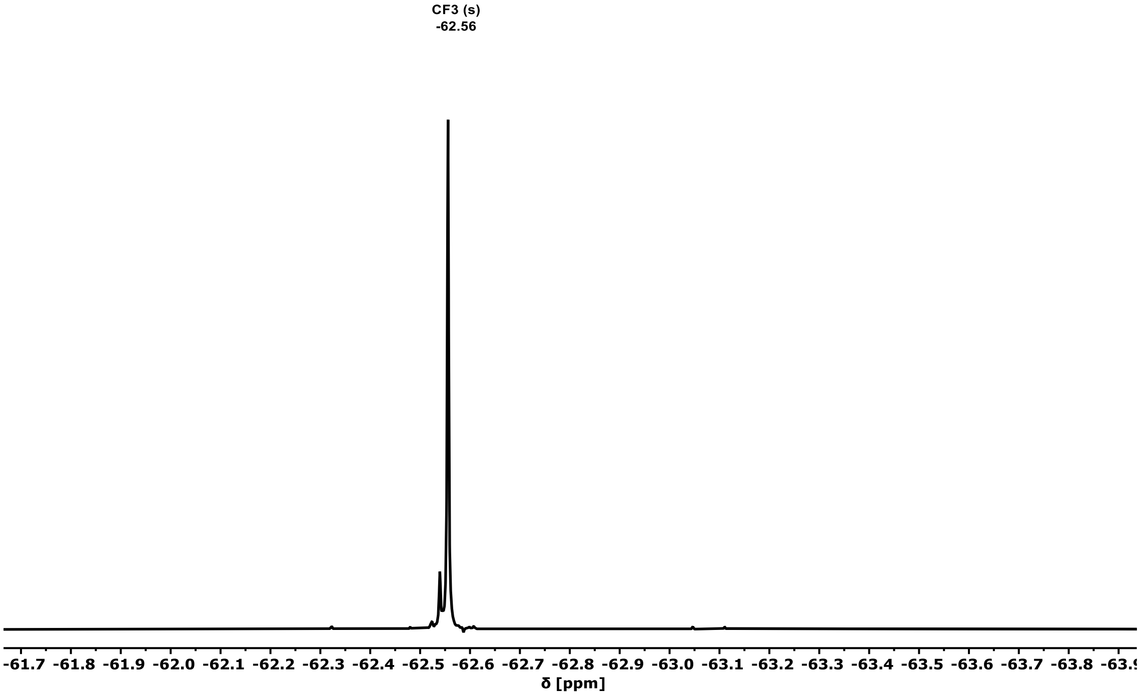

Figure S9. ^{19}F-NMR spectrum ($CDCl_3$, 376 MHz, 300 K) of **2**.

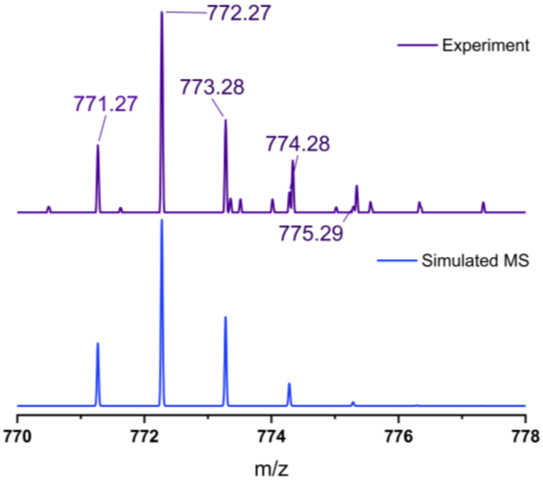

Figure S10. Purple: Molecular ion peak of compounds **2**$^+$ and {**2**+H}$^+$ in the experimental ESI mass spectrum (CH_2Cl_2) with selected mass peaks indicated. Blue: Simulated mass peaks of **2**$^+$ (intensity = 0.4) and {**2**+H}$^+$ (intensity = 1.0).

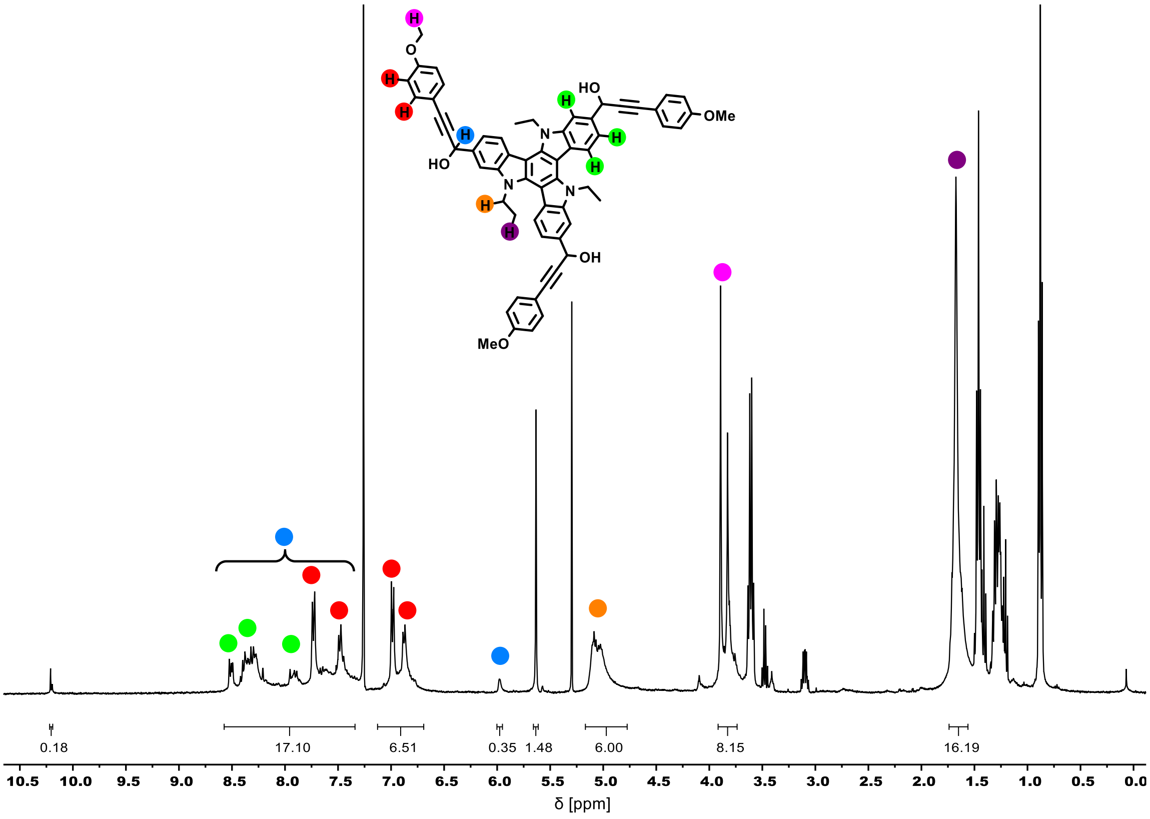

Figure S11. ^{1}H-NMR spectrum ($CDCl_3$, 400 MHz, 300 K) of **3** prior to washing after column chromatography.

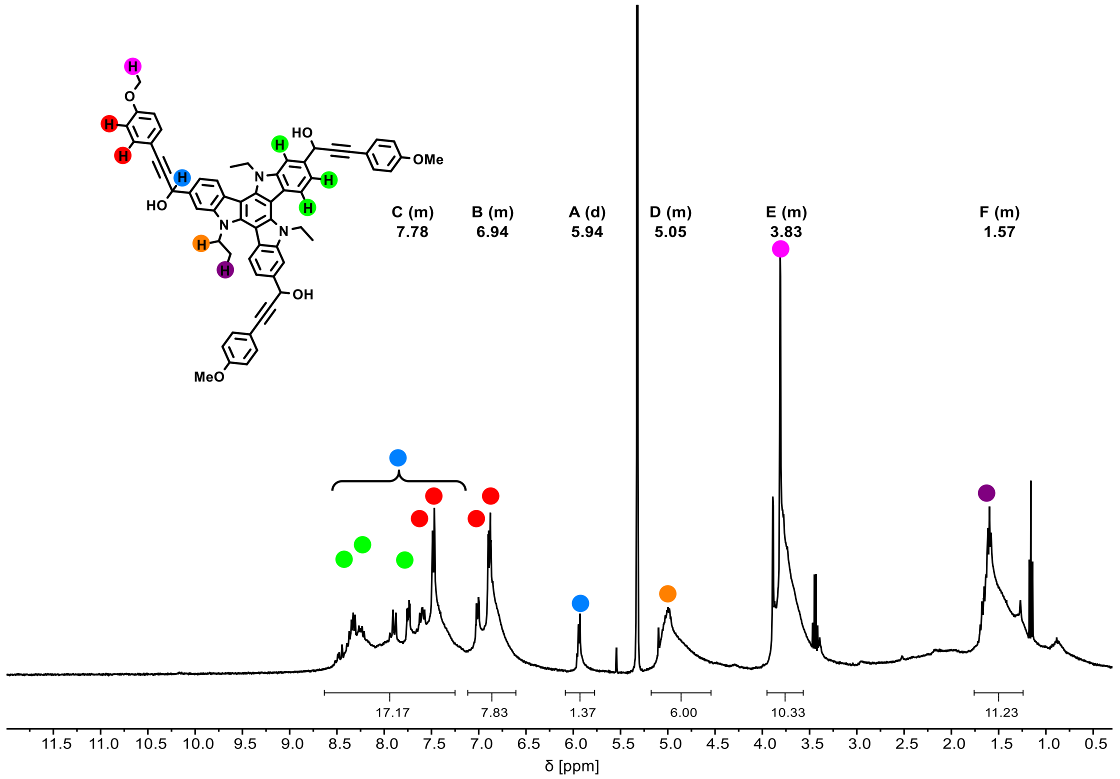

Figure S12. ^{1}H-NMR spectrum ($CDCl_3$, 400 MHz, 300 K) of **3** after column chromatography. These samples were used for PL studies. The presence of aggregates is indicated by the broadening of the signals.

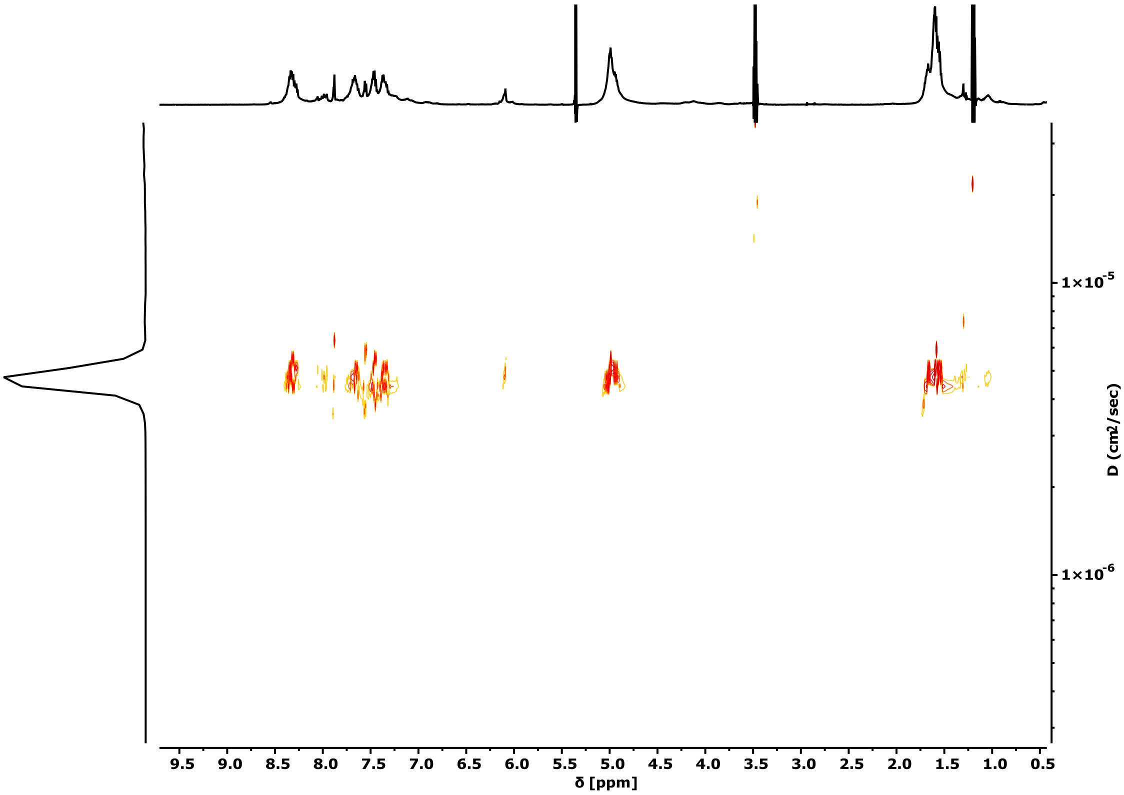

Figure S13. DOSY-NMR spectrum of compound **1** at c = 2.5 mM in CD_2Cl_2 (400 MHz, 300 K). A diffusion coefficient $D = 4.74\cdot10^{-6}$ cm^2/s was obtained, yielding an estimated molecular weight of ca. 3844 g/mol. A value of 0.413 mPa·s was used for the viscosity of CH_2Cl_2 at 300 K.[103–105] The theoretical value of a dimer of **1** is MW = 3748.72 g/mol.

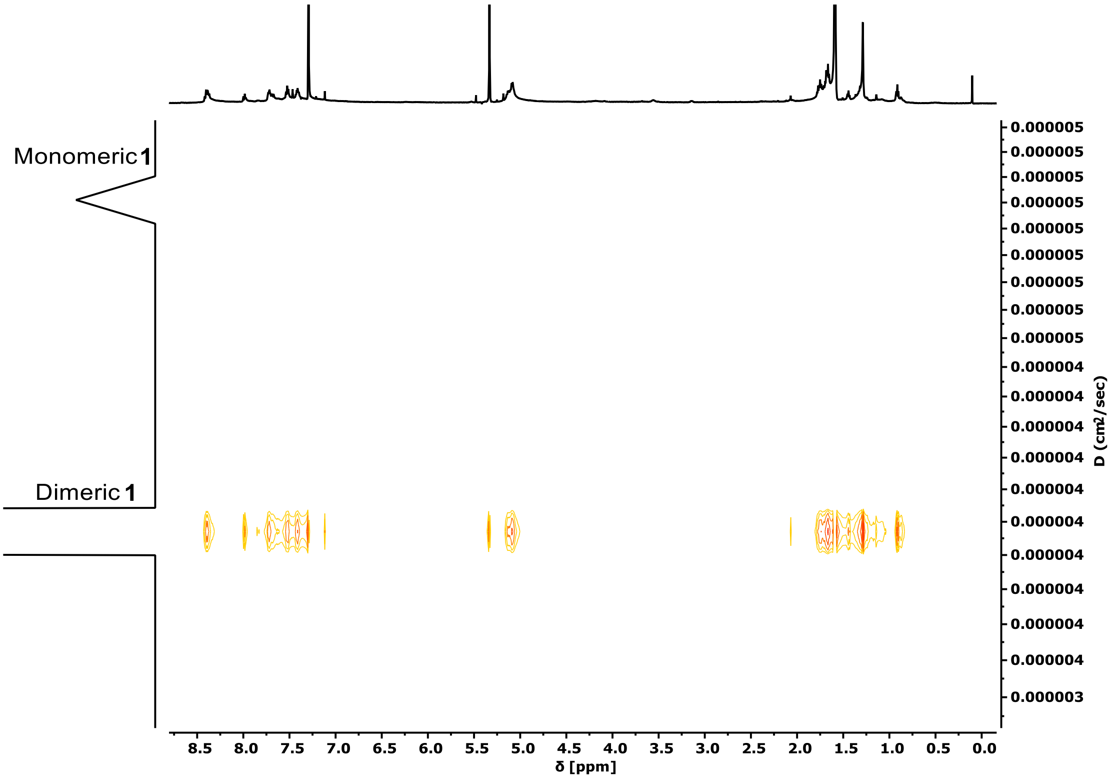

Figure S14. DOSY-NMR spectrum of compound **1** in $CDCl_3$ (400 MHz, 300 K) at c = 0.7 mM. Under these conditions, a diffusion coefficient of $D_1 = 3.82\cdot10^{-6}$ cm^2/s (lower peak with higher intensity) and a diffusion coefficient of $D_2 = 5.10\cdot10^{-6}$ cm^2/s (upper peak, cross peaks not visible due to low intensity) were obtained. The calculated molecular weights are 3744 g/mol for the major, and 1890 g/mol for the minor constituent. A value of 0.533 mPa·s was used for the viscosity of $CHCl_3$ at 300 K.[103,104] The theoretical value of **1** is MW = 1874.36 g/mol for the monomer, and 3748.72 g/mol for the dimer.

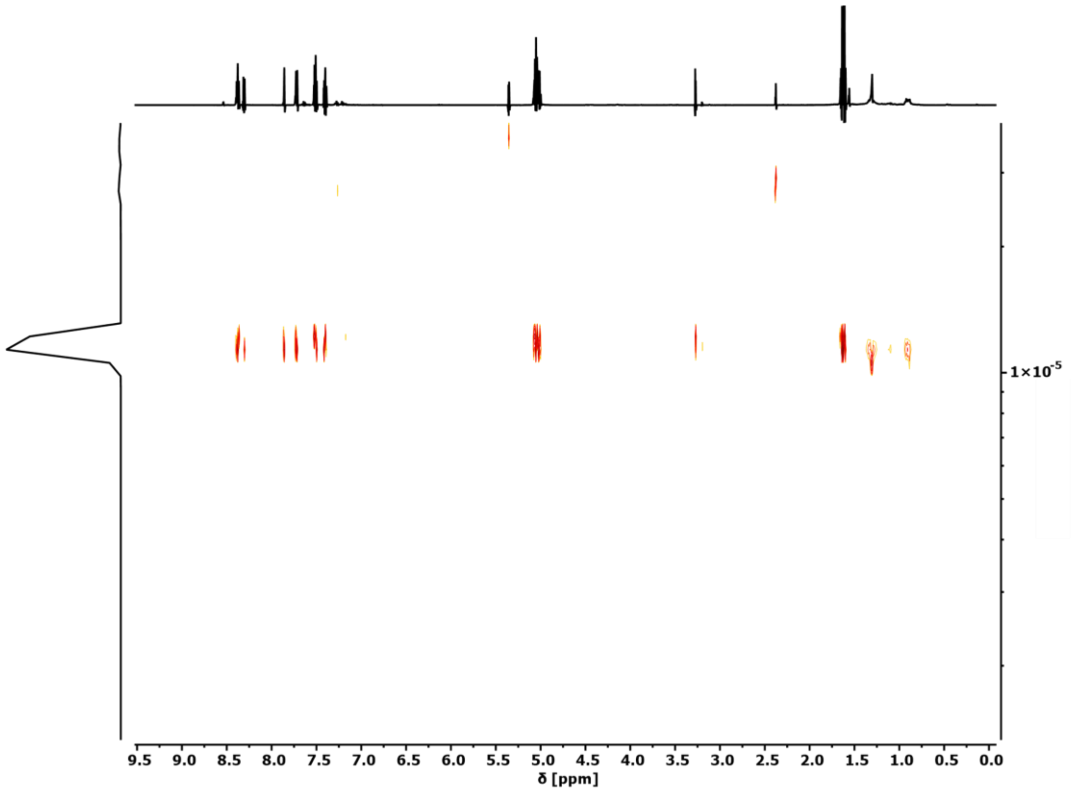

Figure S15. DOSY-NMR spectrum of compound **2-A1-^{Et}TAT** in CD_2Cl_2 (400 MHz, 300 K). A diffusion coefficient $D = 1.19{\cdot}10^{-5}$ cm^2/s was obtained from this measurement, yielding an estimated molecular weight of ca. 469 g/mol. The theoretical value of **2-A1-^{Et}TAT** is MW = 453.59 g/mol. A value of 0.413 mPa·s was used for the viscosity of CH_2Cl_2 at 300 K.[103–105]

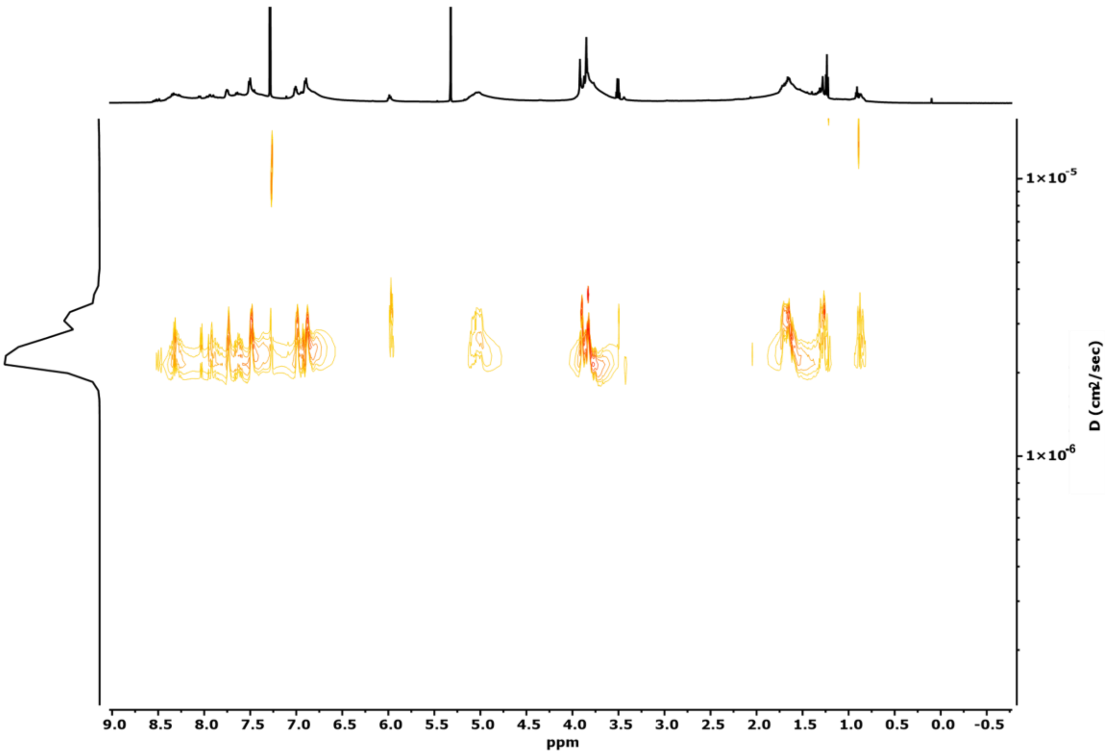

Figure S16. DOSY measurement of compound **3** in $CDCl_3$ (400 MHz, 300 K) in $CDCl_3$. The diffusion coefficient was determined as $D = 2.30{\cdot}10^{-6}$ cm^2/s, yielding an estimated molecular weight of ca. 13450 g/mol. A value of 0.533 mPa·s was used for the viscosity of $CHCl_3$ at 300 K.[103,104]

Voltammetric measurements

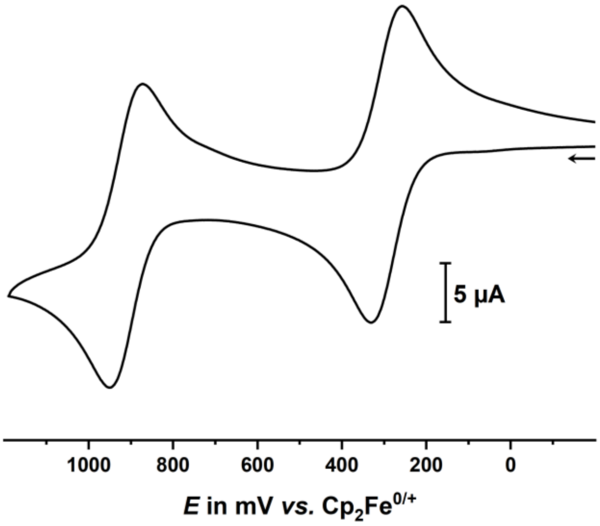

Figure S17. Cyclic voltammogram of **^{Et}TAT** (v = 100 mV/s, CH_2Cl_2, 0.06 M NBu_4^+ PF_6^-, r. t.).

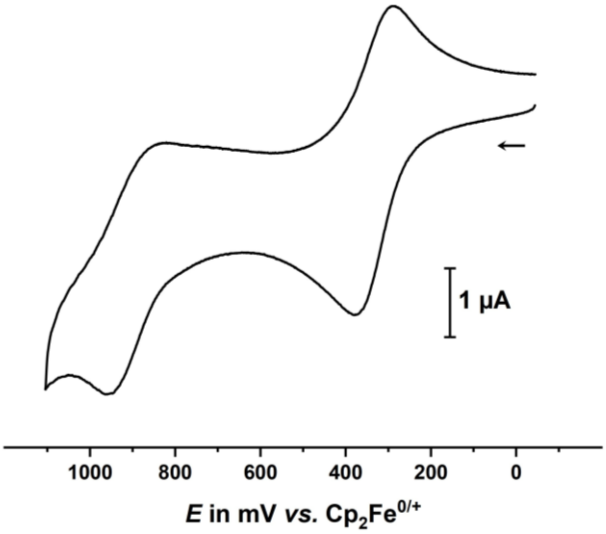

Figure S18. Cyclic voltammogram of **2-A$_1$-^{Et}TAT** (v = 100 mV/s, CH_2Cl_2, 0.06 M NBu_4^+ PF_6^-, r. t.).

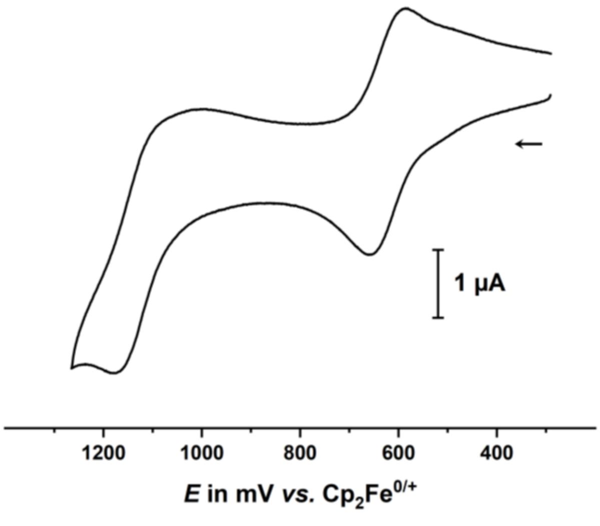

Figure S19. Cyclic voltammogram of **2-CHO$_3$-^{Et}TAT** (v = 100 mV/s, CH_2Cl_2, 0.06 M NBu_4^+ PF_6^-, r. t.).

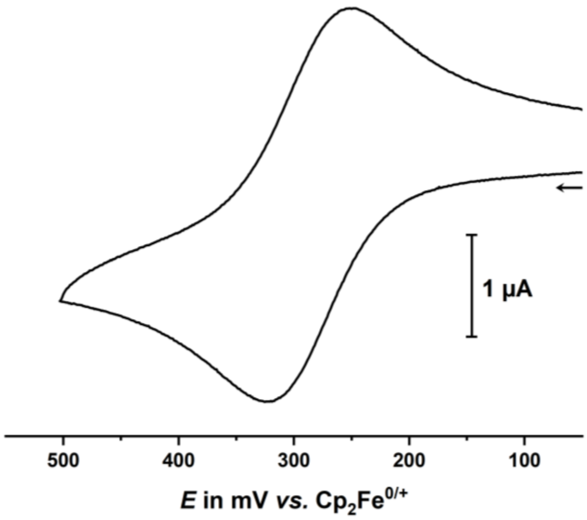

Figure S20. Cyclic voltammogram of **2** (v = 100 mV/s, CH_2Cl_2, 0.04 M NBu_4^+ $[BAr^{F24}]^-$, r. t.).

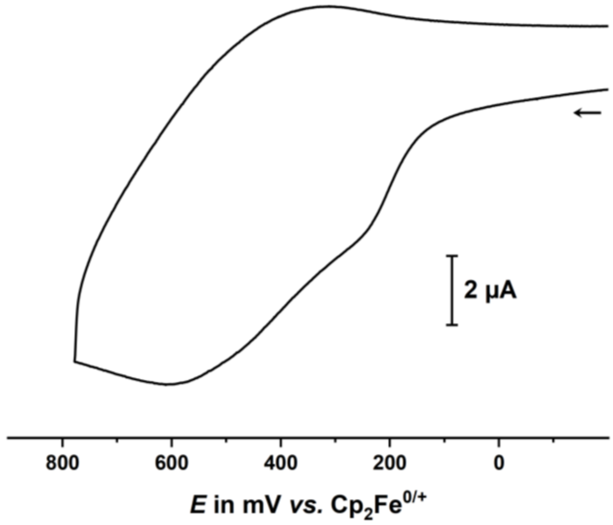

Figure S21. Cyclic voltammogram of **3** (v = 100 mV/s, CH_2Cl_2, 0.04 M NBu_4^+ $[BAr^{F24}]^-$, r. t.).

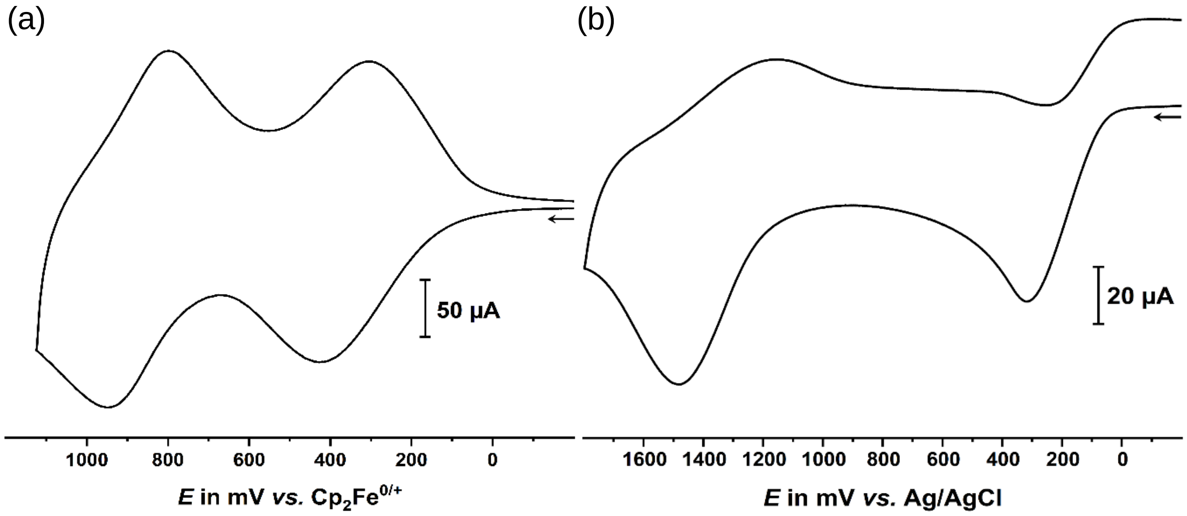

$$i_p = 2.69 \cdot 10^5 \cdot c_{red} \cdot \sqrt{{n_{rel}}^3 \cdot \nu \cdot D_{red}} \; (1)$$

$$n_{rel} = \sqrt[3]{\frac{\left(\frac{i_p}{2.69 \cdot 10^5 \cdot c_{red}}\right)^2}{\nu \cdot D_{red}}} \; (1')$$

Compound **1**: $i_p = 1.60 \cdot 10^{-5} A; c_{red} = 8.75 \cdot 10^{-7} \frac{g}{L}; \nu = 2\frac{V}{s}; D_{red} = 4.74 \cdot 10^{-10} \frac{m^2}{s} \rightarrow$
$n_{rel,compound1} = 1.69$

Cp*₂Fe: $i_p = 1.97 \cdot 10^{-5} A; c_{red} = 5.82 \cdot 10^{-7} \frac{g}{L}; \nu = 2\frac{V}{s}; D_{red} = 1.07 \cdot 10^{-9} \frac{m^2}{s} \rightarrow$
$n_{rel,FeCp2*} = 0.42$

Absolute number of transferred electrons for **1**:

$$n_{absolute,compound1} = \frac{n_{rel,compound1}}{n_{rel,FeCp2*}} = \frac{1.69}{0.42} = 4.03$$

Figure S22 Cyclic voltammogram (a) of compound **1** in CH_2Cl_2/NBu_4^+ $[BAr^{F24}]^-$ (0.04 M) at r. t. and v = 1 V/s and (b) of compound **1** ($c = 8.75 \cdot 10^{-7}$ g·L^{-1}; D_{red} was taken from Figure S13) with added Cp^*_2Fe ($c = 5.82 \cdot 10^{-7}$ g·L^{-1}, D_{red} taken from ref. [106]) in CH_2Cl_2/NBu_4^+ $[BAr^{F24}]^-$ (0.04 M) at r. t. and at v = 2 V/s, and the calculation of the transferred electrons per wave. Addition of Cp^*_2Fe decreased the reversibility of the oxidation wave of **1**.

UV/vis/NIR and IR spectroscopy and spectroelectrochemistry

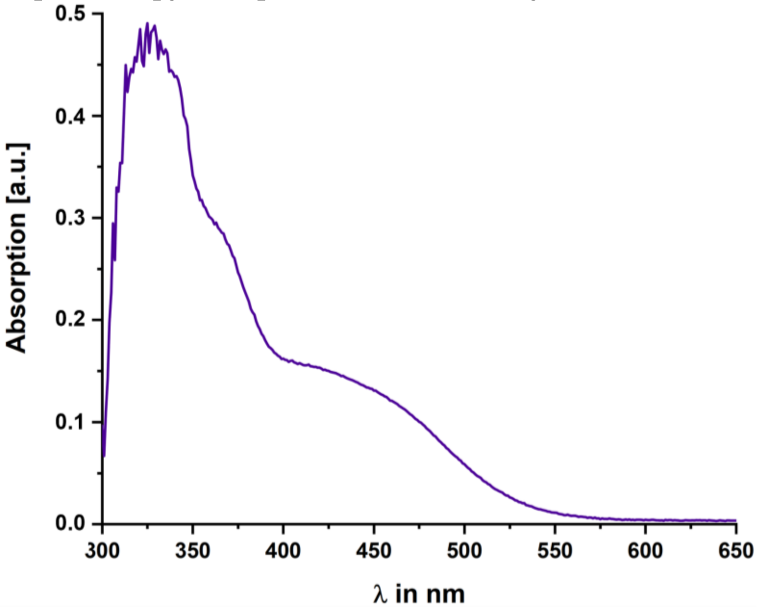

Figure S23. Electronic absorption spectrum of a film of **1**, spin-coated on glass.

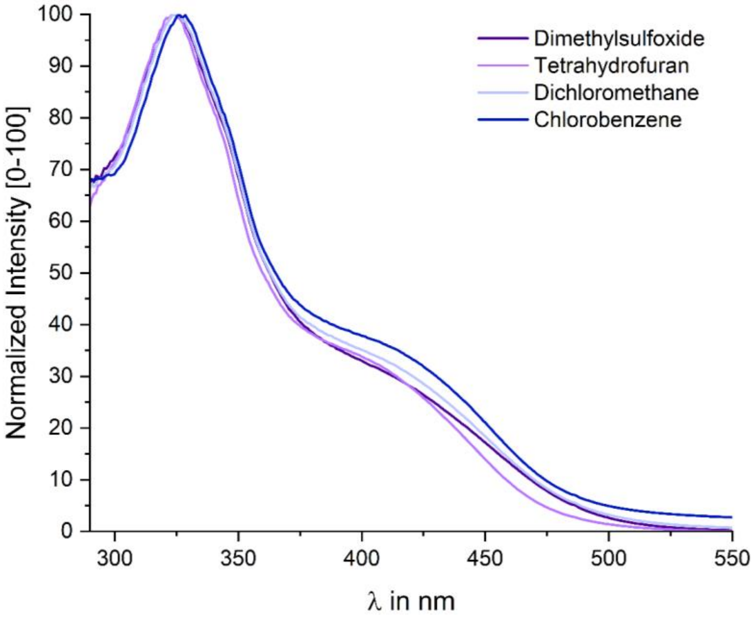

Figure S24. UV/vis/NIR spectra of compound **3** in different solvents at r. t.

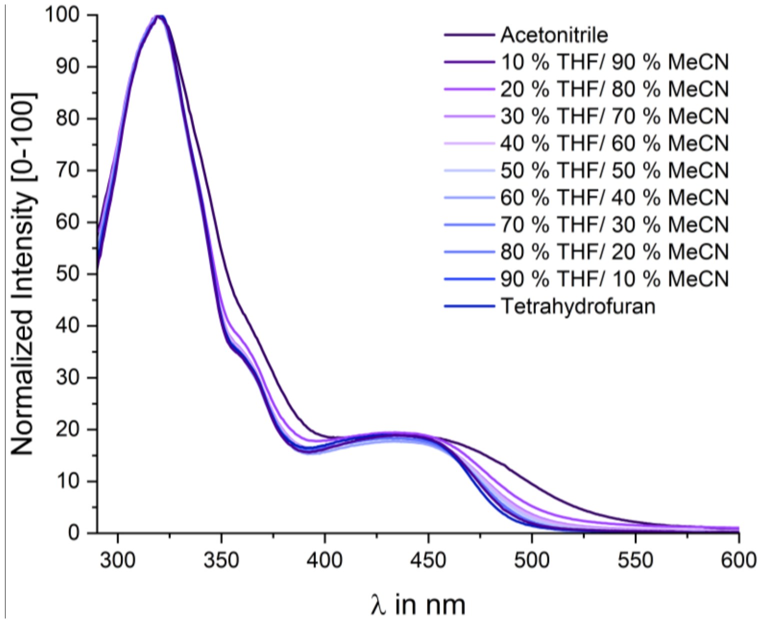

Figure S25. UV/vis/NIR spectra of **1** in different mixtures of THF and acetonitrile at r. t.

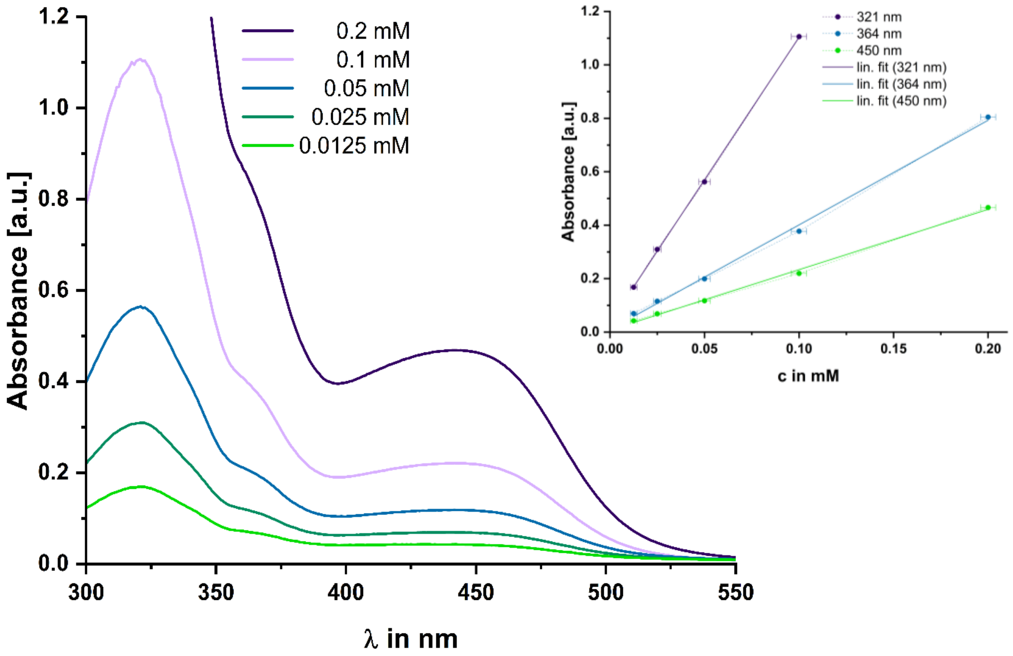

Figure S26. UV/Vis spectra of **1** recorded at the indicated concentrations in CH_2Cl_2 at r. t. The inset is the corresponding Lambert-Beer plot with added linear regressions (propagating error of 2.5 % of the concentration) for different bands. Positions are given in the legend in nm.

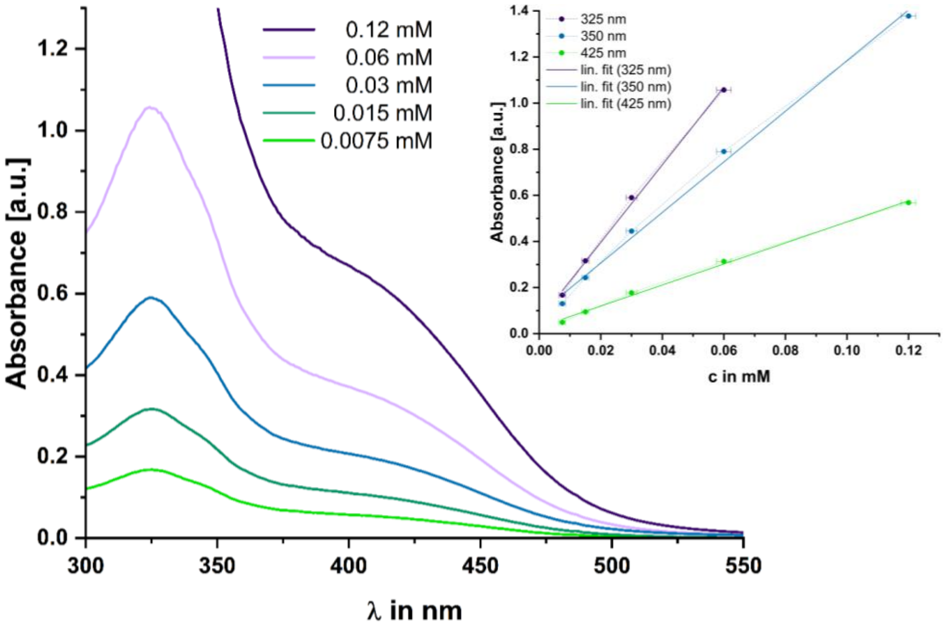

Figure S27. UV/Vis spectra of **3** recorded at different concentrations in CH_2Cl_2 at r. t. The inset provides the corresponding Lambert-Beer plot with added linear regressions (propagating error of 2.5 % of the concentration) for different bands. Positions are given in the legend in nm.

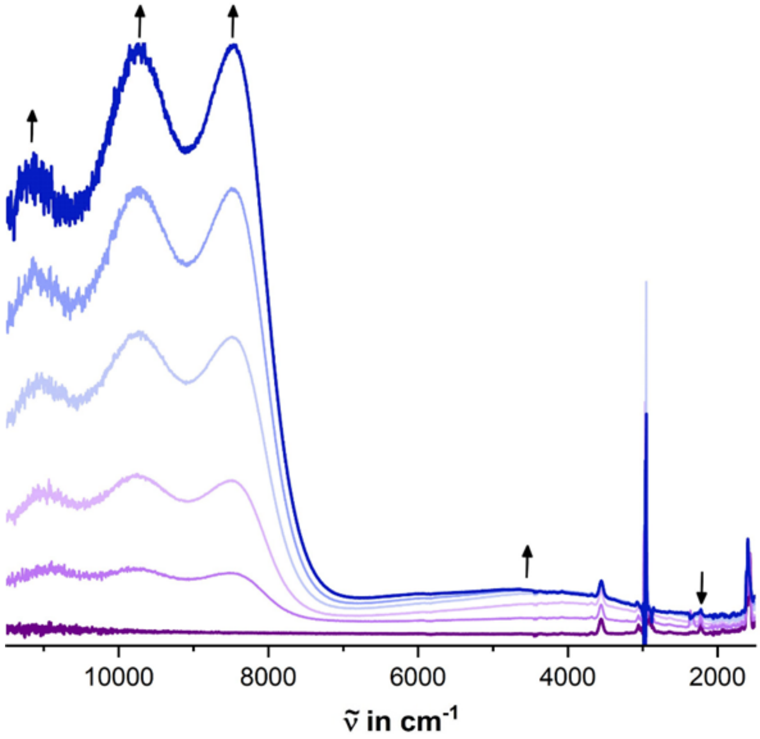

Figure S28. Changes in the IR/NIR spectrum of **2** recorded during electrolysis in an OTTLE cell in the 0.14 M 1,2-$C_2H_4Cl_2$/NBu_4^+ $[BAr^{F24}]^-$ electrolyte at r. t.

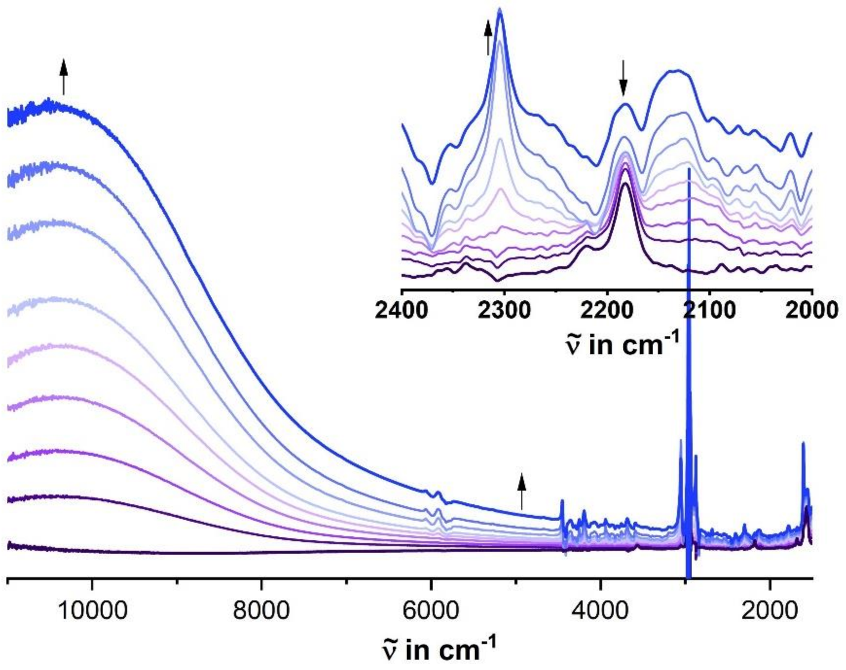

Figure S29. Changes in the IR/NIR spectrum and of the alkyne stretching vibrations (see insert) of **1**, as recorded during electrolysis in an OTTLE cell in the 0.14 M 1,2-$C_2H_4Cl_2$/NBu_4^+ $[BAr^{F24}]^-$ electrolyte at r. t.

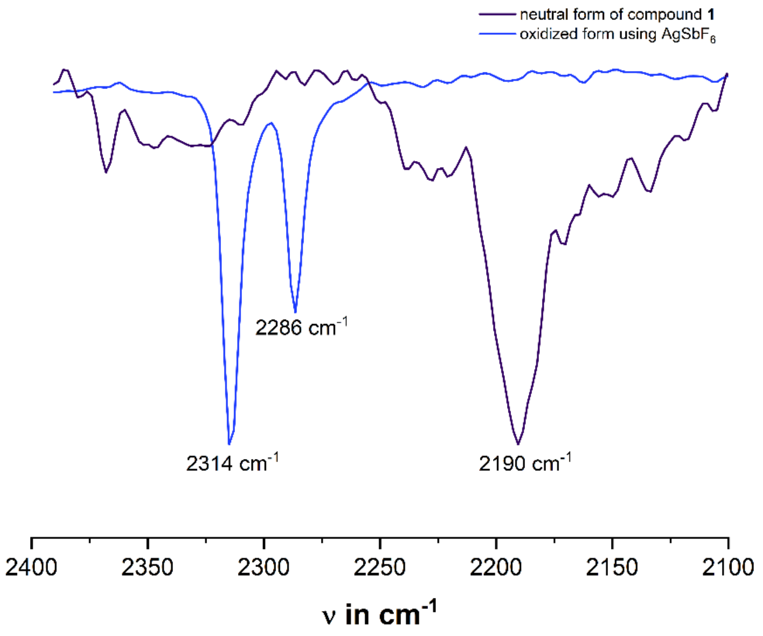

Figure S30. Alkyne stretching vibrations of **1** and of $\mathbf{1}^{4+}$ as recorded on an ATR-FT-IR spectrometer. The oxidized form $\mathbf{1}^{4+}$ was generated by oxidizing **1** with excess (6 equiv.) of Ag^+ SbF_6^- in CH_2Cl_2. The precipitated Ag was removed through centrifugation and the solvent was removed under reduced pressure.

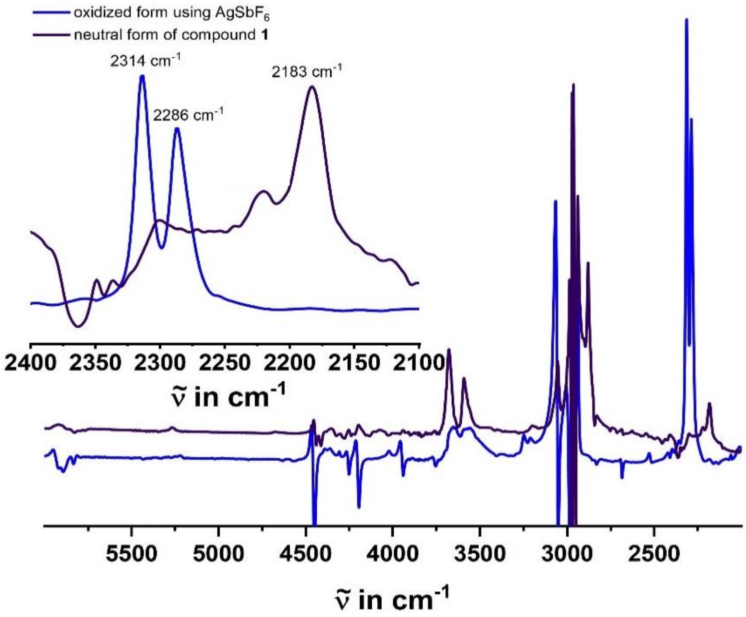

Figure S31. Alkynyl stretching vibrations of **1** and of $\mathbf{1}^{4+}$ in CH_2Cl_2. $\mathbf{1}^{4+}$ was generated by oxidizing **1** with excess (6 equiv.) of $AgSbF_6$.

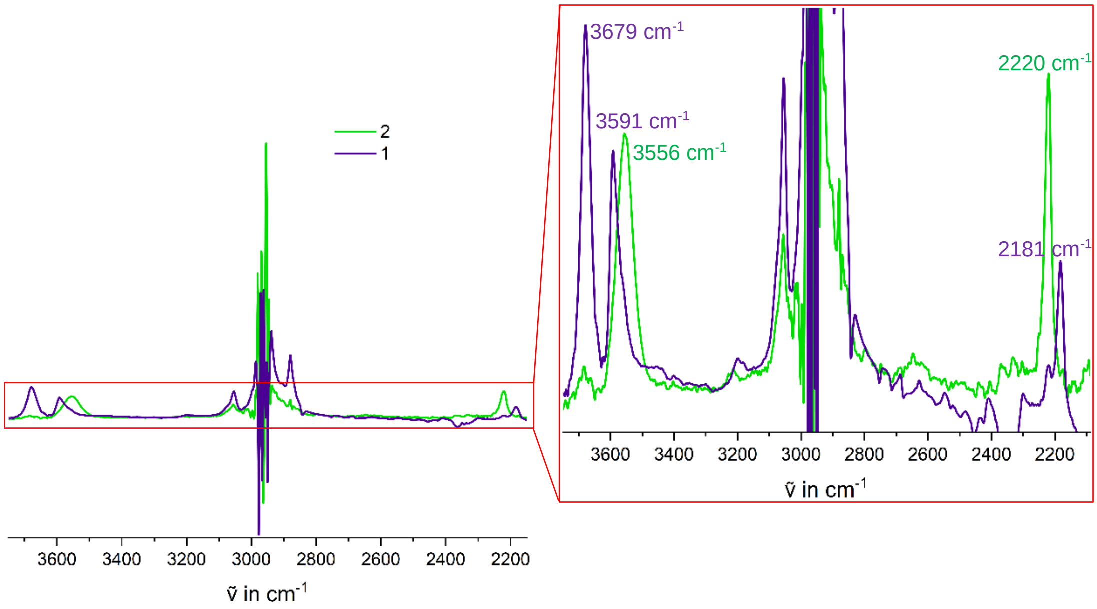

Figure S32. Comparison of IR spectra of the alkynyl C≡C and the O-H stretching vibrations of compound **1** (purple) and **2** (green) in 1,2-$C_2H_4Cl_2$.

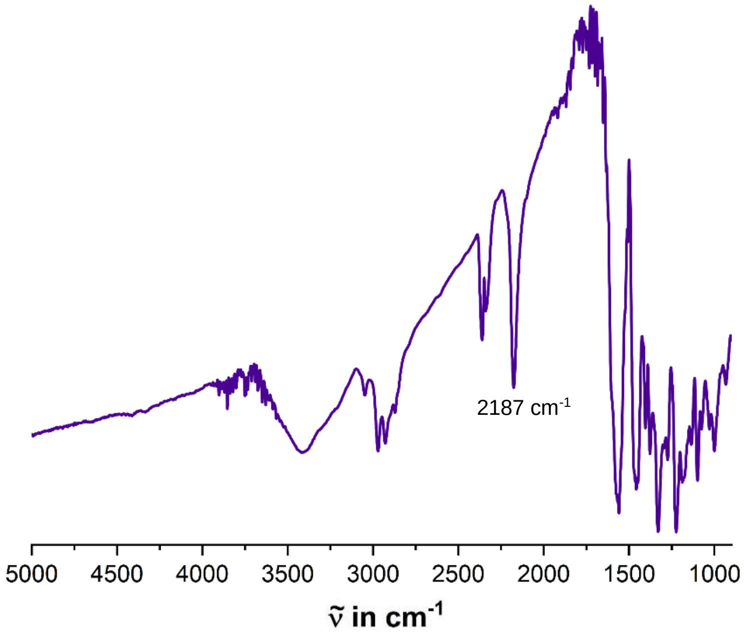

Figure S33. IR spectrum of **1** recorded as a KBr-pellet in the transmittance mode. The energy of the alkynyl C≡C stretching mode is indicated.

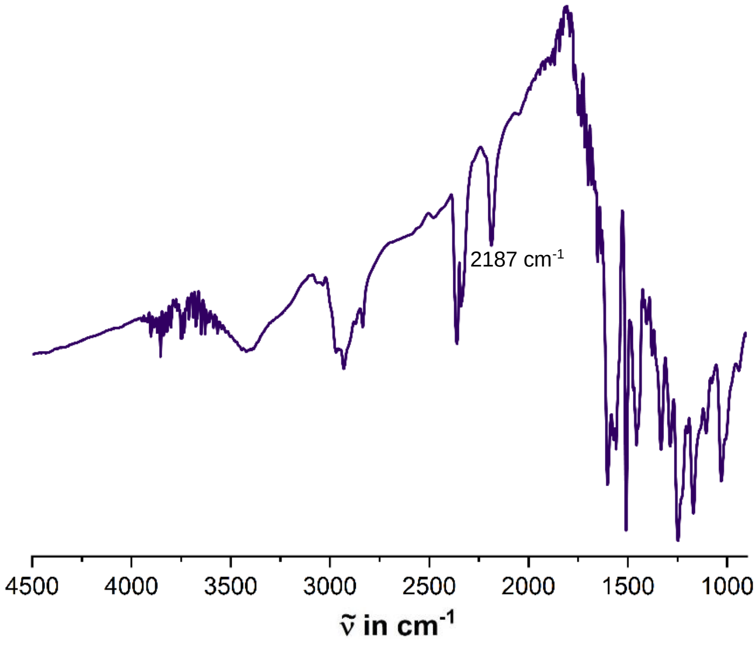

Figure S34. IR spectrum of **3** recorded as a KBr-pellet in the transmittance mode. The energy of the alkynyl C≡C stretching vibration is indicated.

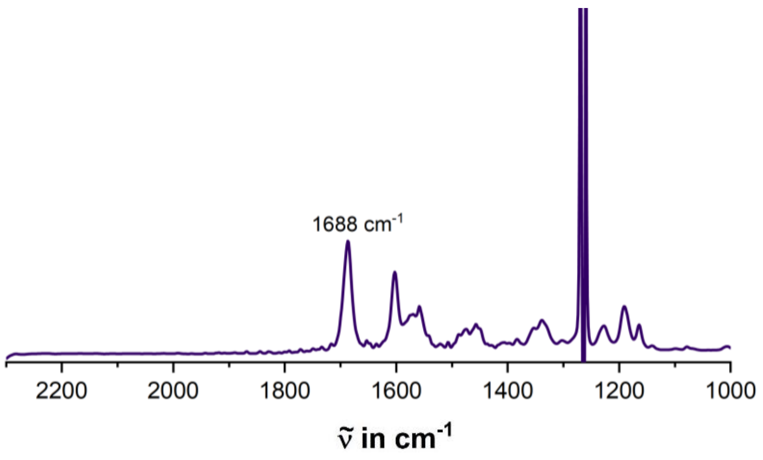

Figure S35. IR spectrum of **2-CHO3-^{Et}TAT** recorded in CH_2Cl_2. The energy of the C=O stretching mode is indicated.

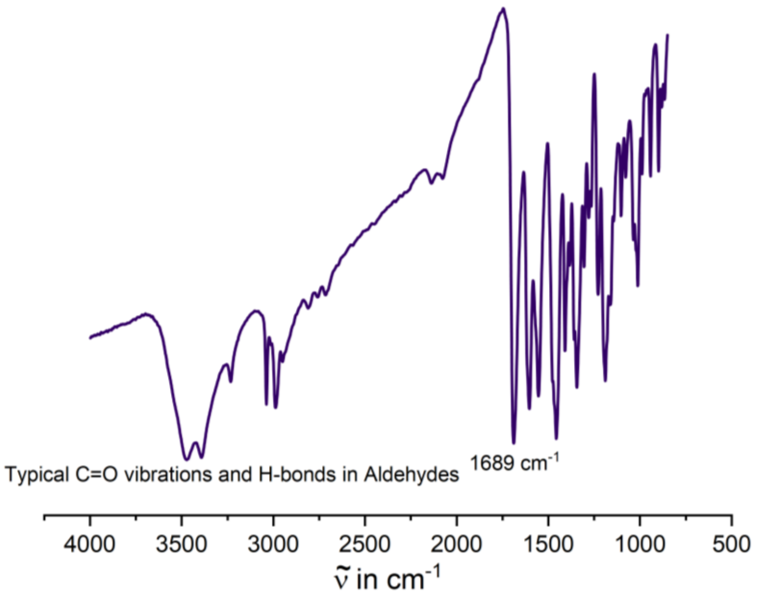

Figure S36. IR spectrum of **2-CHO3-^{Et}TAT** recorded as a KBr-pellet in the transmittance mode. The energy of the C=O stretching mode is indicated.

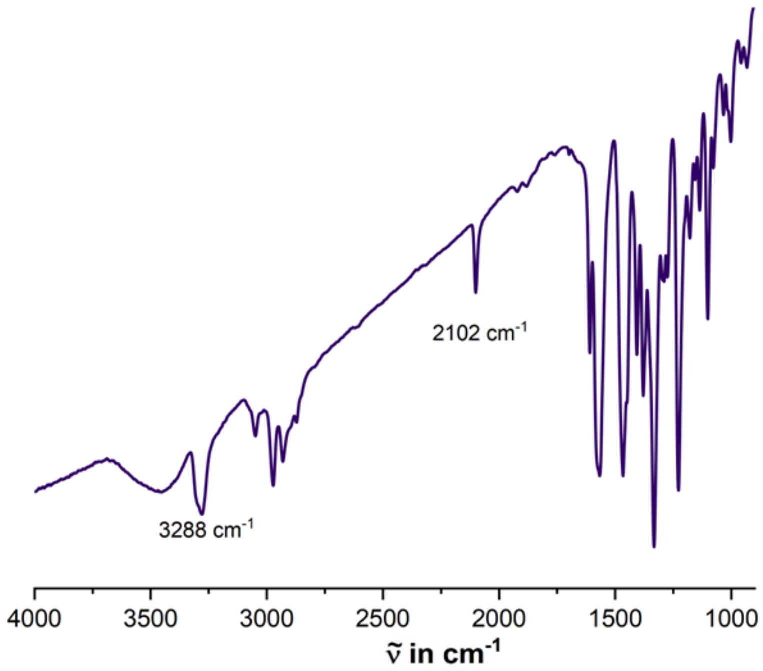

Figure S37. IR spectrum of **2-A$_1$-^{Et}TAT** recorded as a KBr-pellet in the transmittance mode with the energies of the alkynyl ≡C-H and C≡C vibrations.

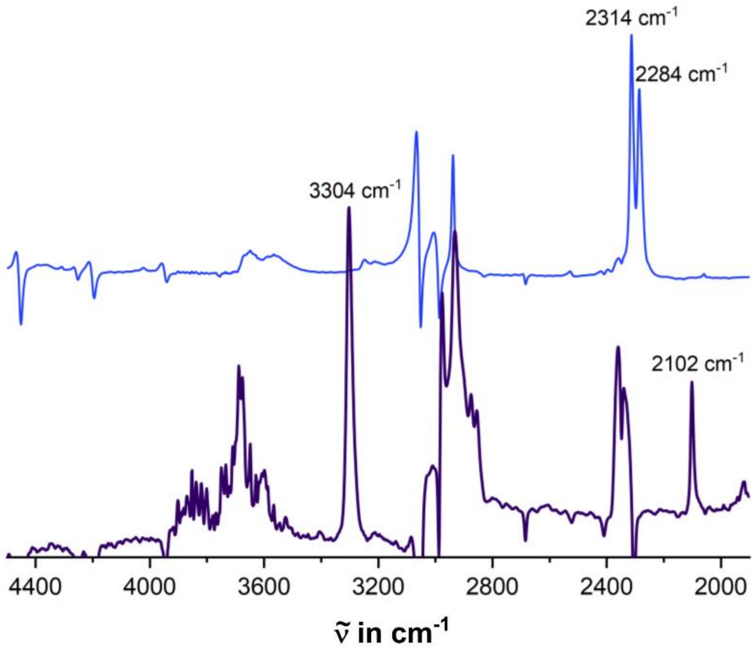

Figure S38. IR spectrum of neutral (bottom, purple curve) and the one-electron oxidized form (top, blue curve) of **2-A$_1$-^{Et}TAT** in CH_2Cl_2. The oxidized form was generated using an excess of >1 equiv. of Ag^+ SbF_6^-. The energies of the alkynyl ≡C-H and C≡C vibrations are indicated.

Photoluminescence measurements

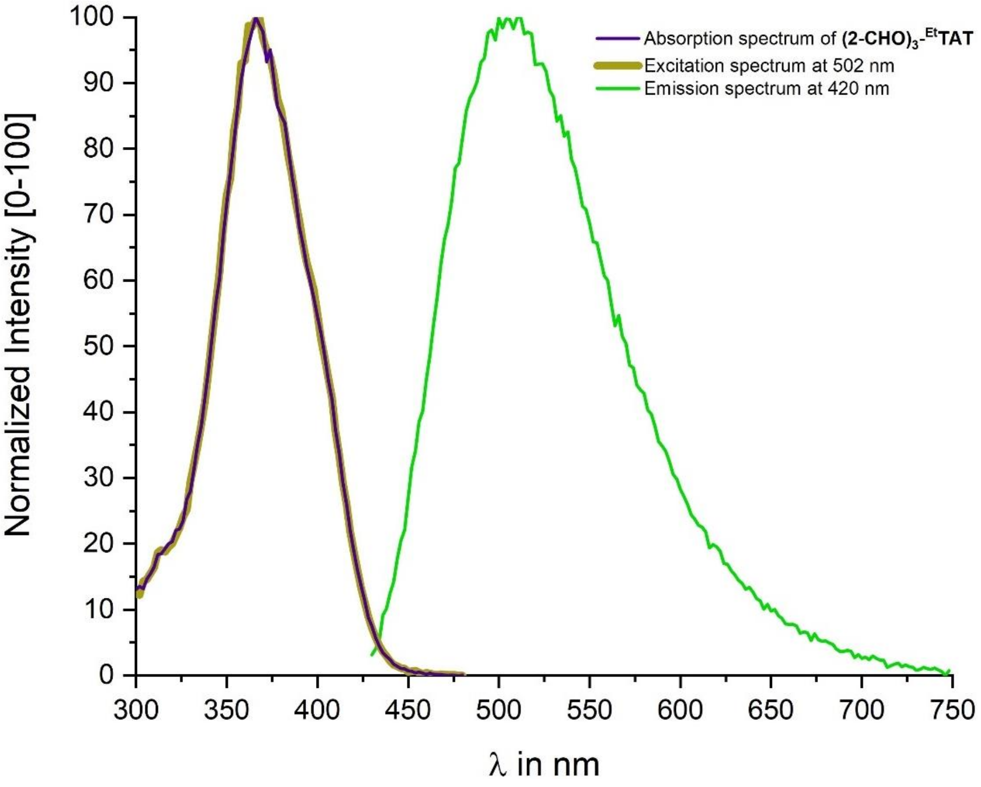

Figure S39. Normalized absorption, emission and excitation spectra of **2-CHO$_3$-^{Et}TAT** at λ_{exc} = 420 nm. The spectra were recorded on a CH_2Cl_2 solution at r. t.

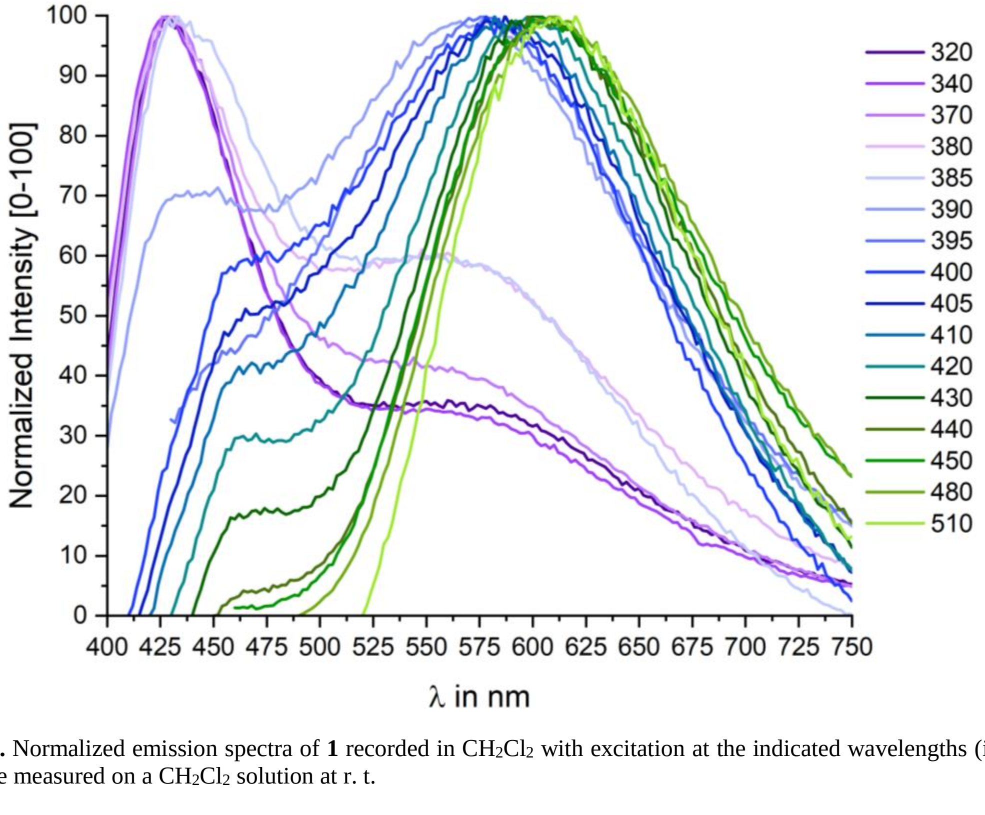

Figure S40. Normalized emission spectra of **1** recorded in CH_2Cl_2 with excitation at the indicated wavelengths (in nm). The spectra were measured on a CH_2Cl_2 solution at r. t.

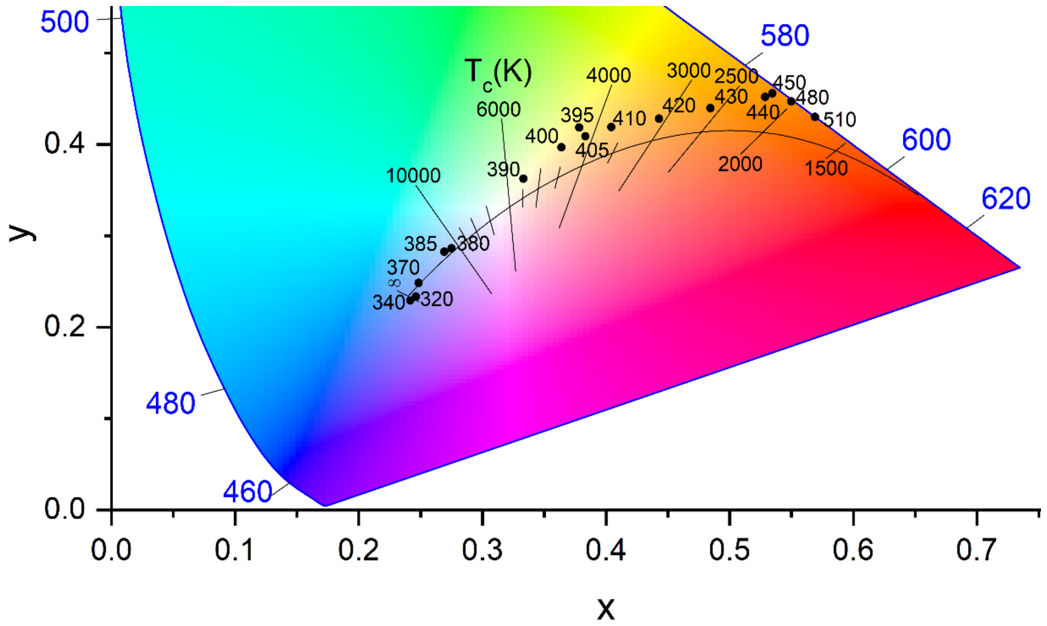

Figure S41. CIE color temperature (1931) plot for emission spectra of **1** recorded in CH_2Cl_2 at the indicated excitation wavelength.

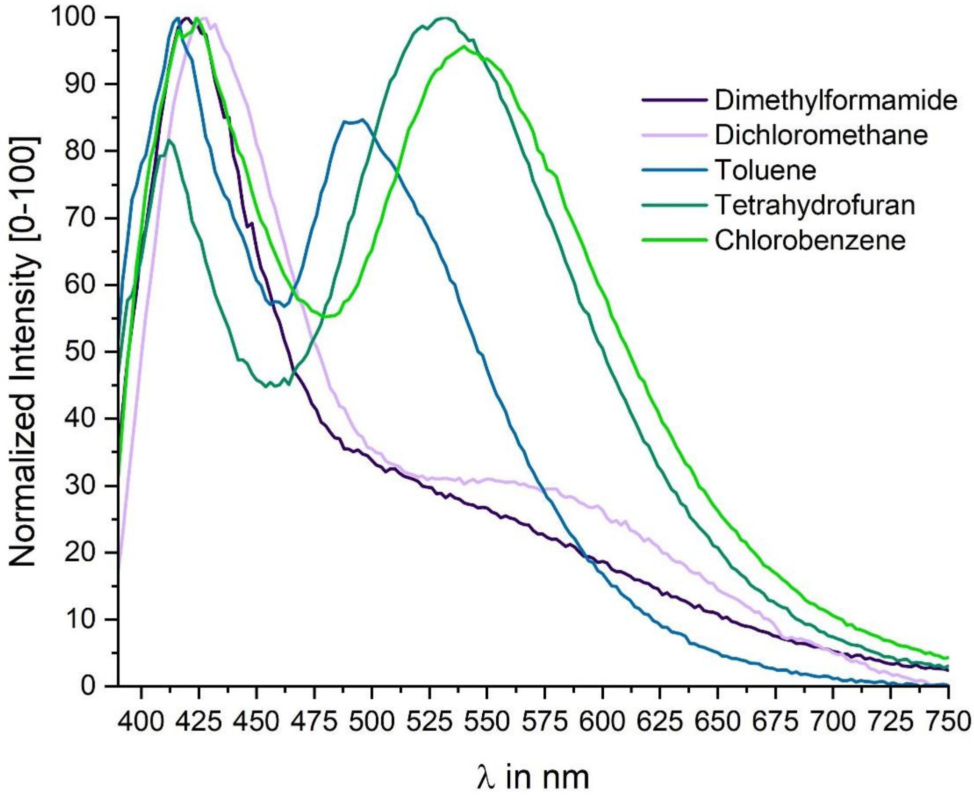

Figure S42. Normalized emission spectra of **1** recorded in different solvents with excitation at 340 nm. The spectra are measured at r. t.

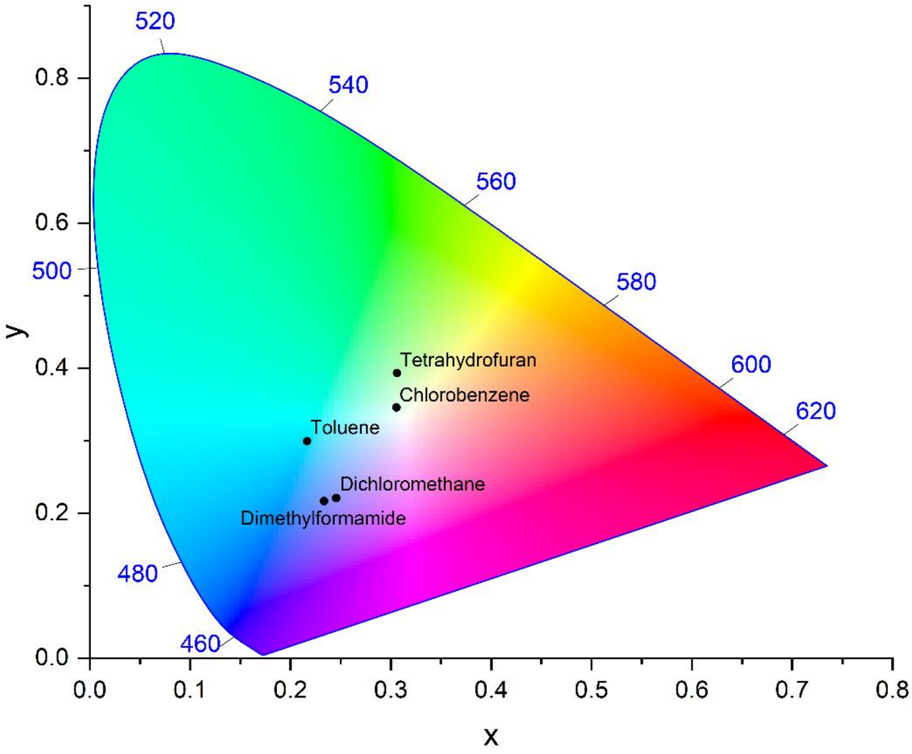

Figure S43. CIE color temperature plot (1931) of the PL spectra of **1** with excitation at 340 nm in different solvents, as shown in Figure S40. Tetrahydrofuran: x = 0.30, y = 0.39; R = 134, G = 180, B = 138; #83AF8B; dark seagreen; dimethylformamide: x = 0.23, y = 0.22; R = 108, G = 125, B = 196; #6D7DC5, cornflower blue; dichloromethane: x = 0.25, y = 0.22; R = 120, G = 123, B = 193; #777DC0; cornflower blue; toluene: x = 0.22, y = 0.30; R = 0, G = 164, B = 181; #00A1B3, dark cyan; chlorobenzene: x = 0.31, y = 0.35, blueish-white.

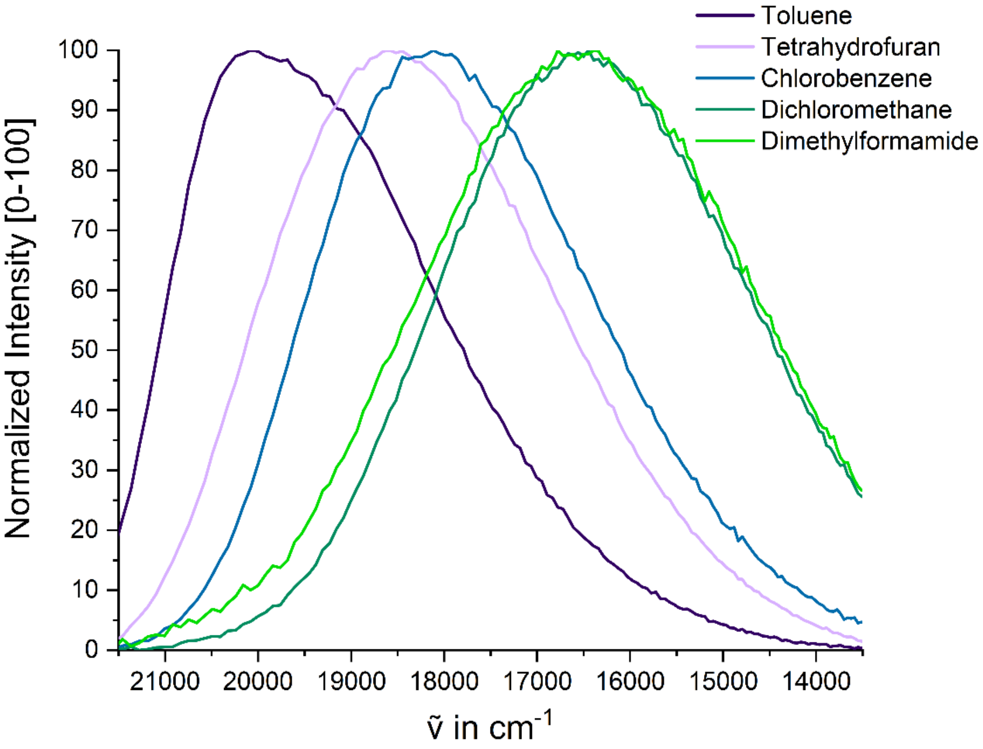

Figure S44. Normalized emission spectra of **1** in different solvents, recorded at an excitation wavelength of 450 nm. The spectra were recorded at r. t.

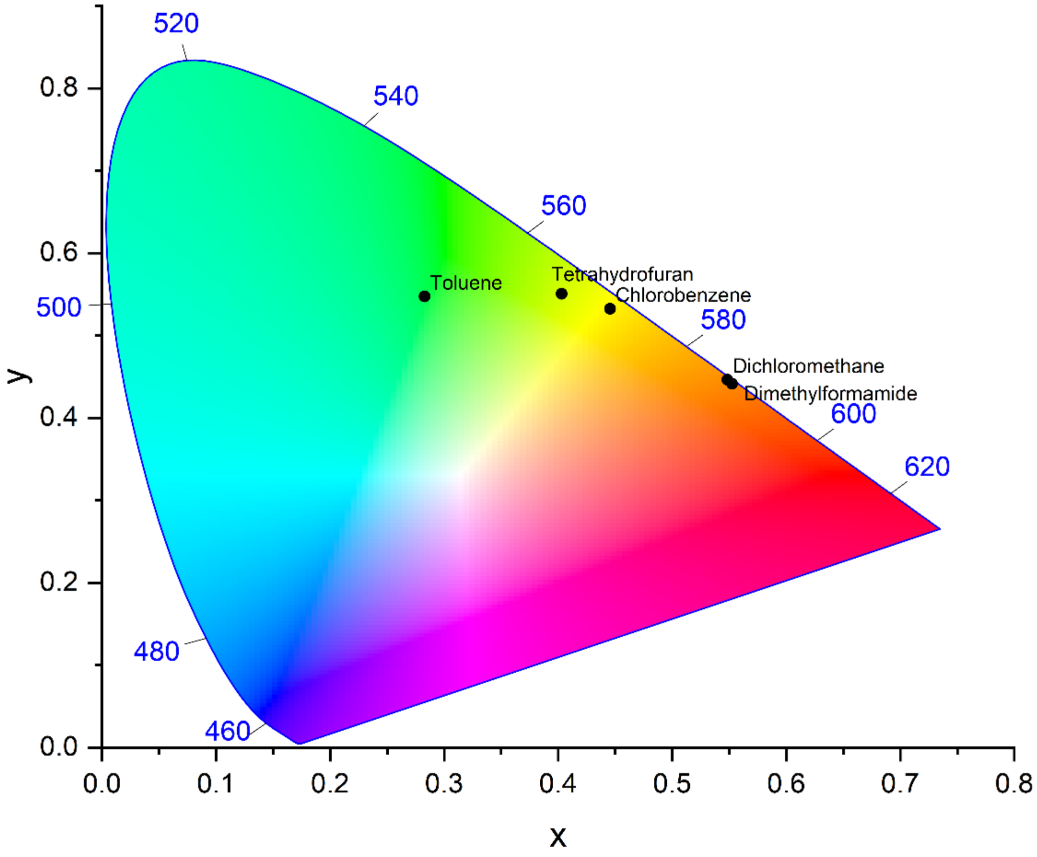

Figure S45. CIE color temperature plot (1931) of the PL spectra of **1** in different solvents with excitation at 450 nm, as shown in Figure S42. Toluene: x = 0.28, y = 0.55; R = 0, G = 226, B = 82 #00E252; lime green; tetrahydrofuran: x = 0.40, y = 0.55; R = 176, G = 210, B = 0; #B2D100; yellow-green; chlorobenzene: x = 0.45, y = 0.53; R = 205, G = 199, B = 0; # CDC900; golden; dichloromethane: x = 0.55, y = 0.45; R = 255, G = 150, B = 0; #FF9500; dark orange; dimethylformamide: x = 0.55, y = 0.44; R = 255, G = 147, B = 0; #FF9500; dark orange.

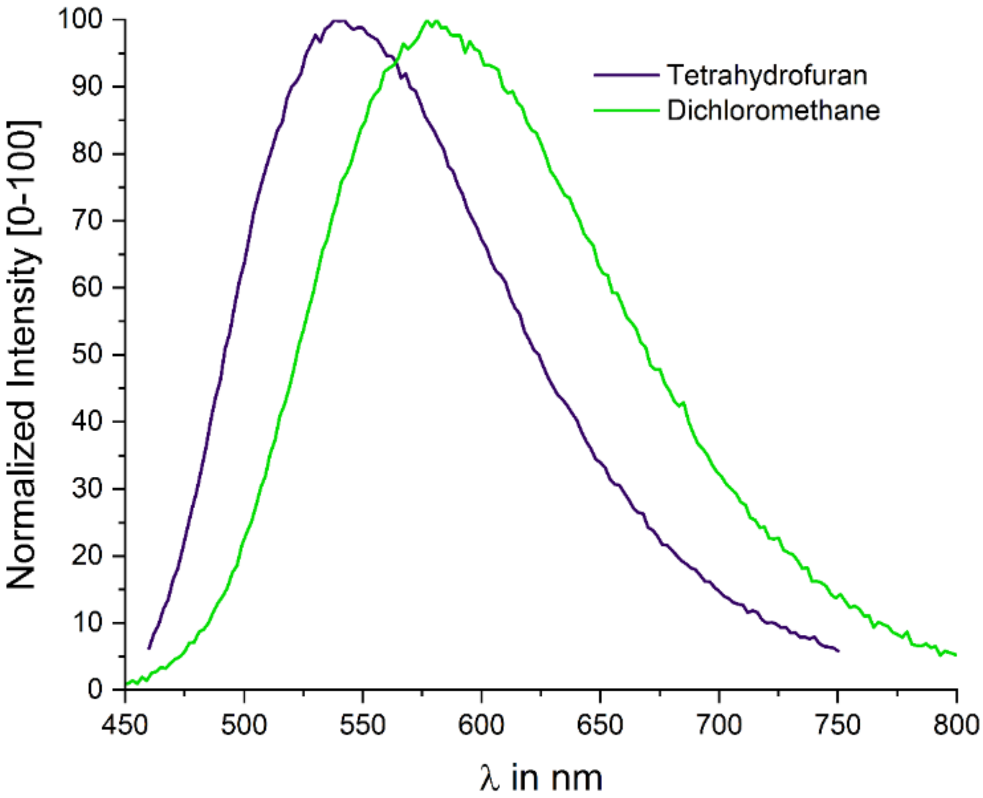

Figure S46. Normalized emission spectra of **3** in CH_2Cl_2 (λ_{exc} = 450 nm) and in THF (λ_{exc} = 450 nm) at r. t.

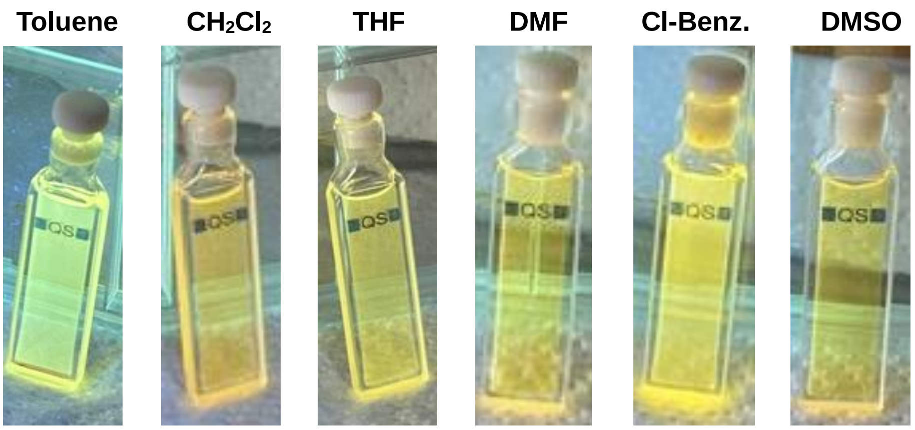

Figure S47. Optical impression of the emission color of **3** in different solvents at r. t., triggered with 365 nm handheld UV lamp.

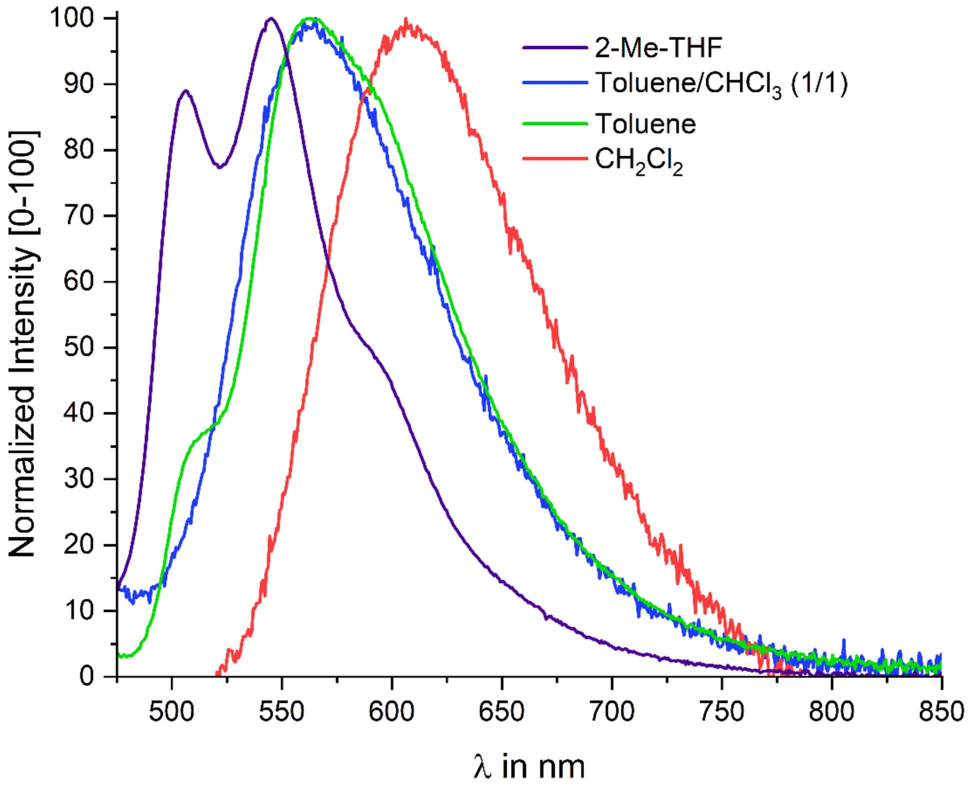

Figure S48. Normalized emission spectra of **1** recorded with excitation at 320 nm in different solvents at 77 K as given in the legend. The spectra were recorded in a glassy matrix of 2-methyl-THF and frozen solutions of the other solvents.

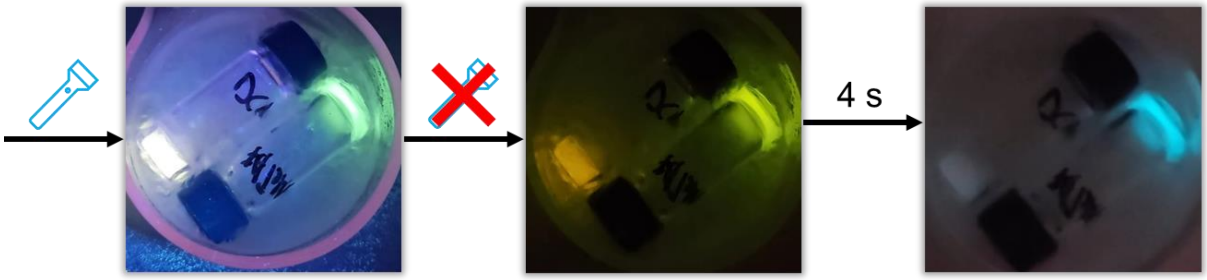

Figure S49. Temporal evolution of the afterglow of compound **1** in frozen CH_2Cl_2 (top vial, compound on the left-hand side) and frozen 2-methyl-THF (bottom vial, compound at the right-hand side) excited using a handheld UV lamp with an excitation wavelength of 365 nm.

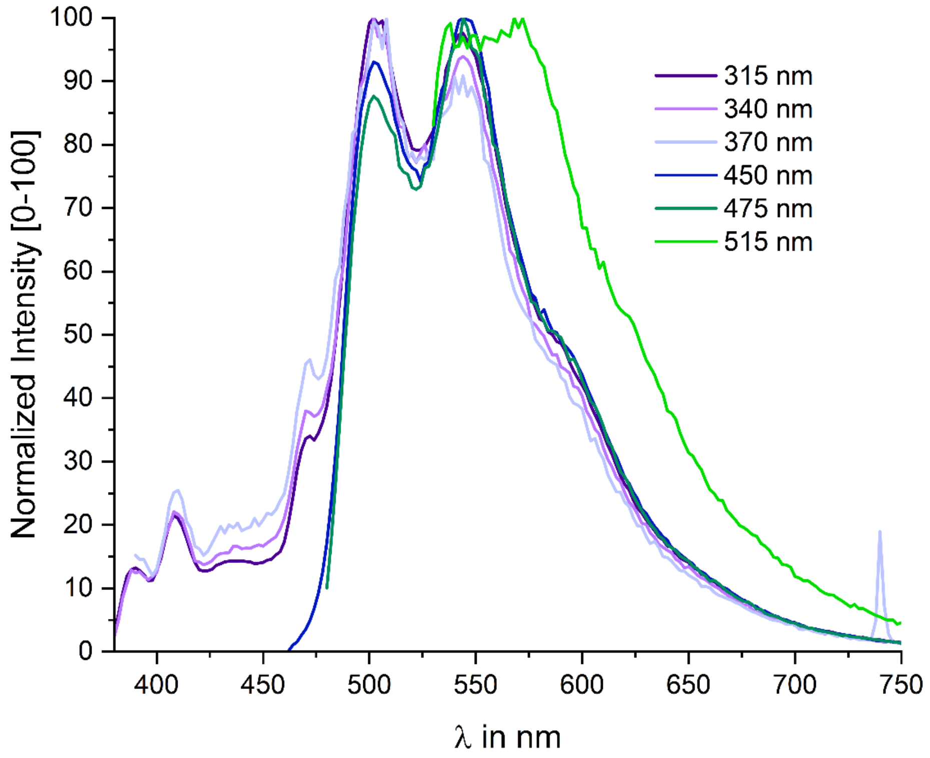

Figure S50. Normalized emission spectra of **1** recorded at different excitation wavelengths as given in the legend. The spectra were recorded in a glassy matrix of 2-methyl-THF at 77 K.

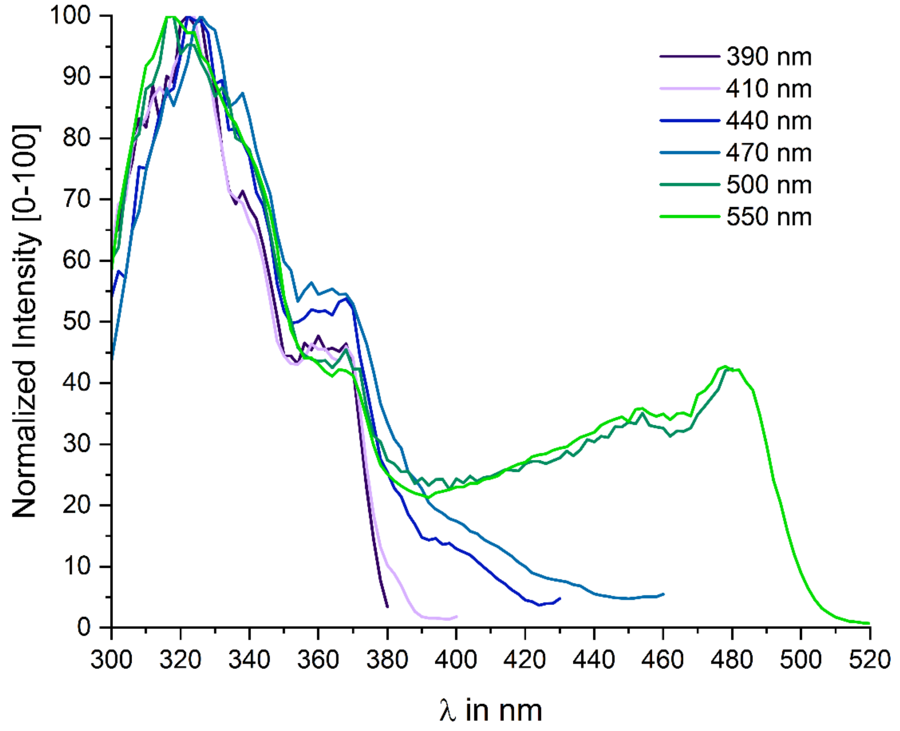

Figure S51. Normalized excitation spectra of **1** recorded at different emission wavelengths. The spectra were recorded in a glassy matrix of 2-methyl-THF at 77 K.

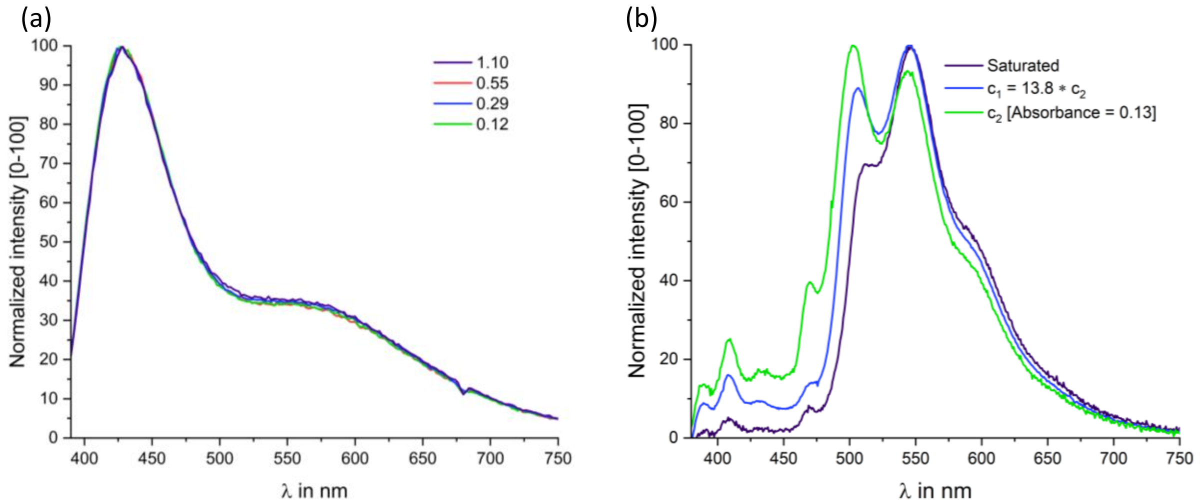

Figure S52. Normalized emission of solutions of **1** at different concentrations in (a) CH_2Cl_2 at r. t., and (b) as a glassy matrix in 2-methyl-THF recorded at an excitation wavelength of 320 nm.

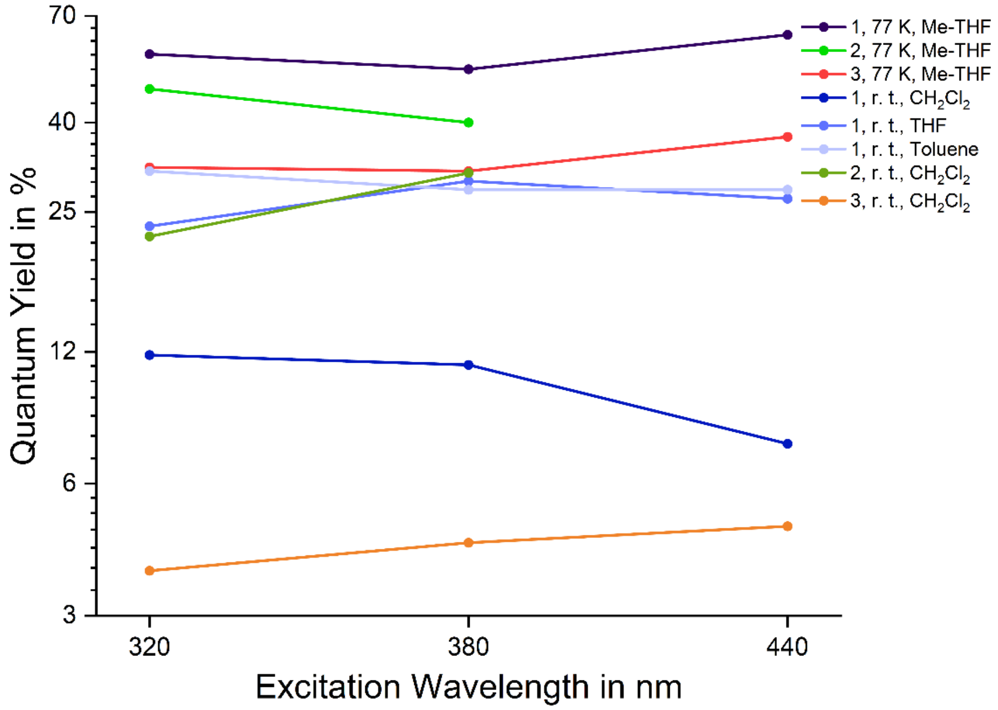

Figure S53. Quantum yields of compounds **1**, **2** and **3** at excitation wavelengths of 320, 380 and 440 nm. The lines are intended as a guide to the eye. Quantum yields were measured at 77 K in 2-methyl-THF and at r. t. in CH_2Cl_2 for all three compounds. For compound **1**, quantum yields were additionally determined in THF and in toluene. The data is also provided in Table S1 below.

Table 1. Quantum yields (QYs) of compounds **1**, **2** and **3** at excitation wavelengths of 320, 380 and 440 nm.

	77 K, 2-methyl-THF			*r. t., CH_2Cl_2*			*r. t., THF*			*r. t., toluene*		
**λ_{exc} [nm]**	320	380	440	320	380	440	320	380	440	320	380	440
**1**	57.3[a]	52.9	63.4	11.8	11.2	7.4	23.2	29.4	28.2	31.0	28.1	28.1
**2**	47.7[b]	40.0	-	22.0	30.7	-	-	-	-	-	-	-
**3**	31.6	31.0	37.1	3.8	4.4	4.8	-	-	-	-	-	-

[a] Phosphorescence: QY = 53.7%, Fluorescence: QY = 3.6%. [b] Phosphorescence: QY = 39.4%, Fluorescence: QY = 8.3%.

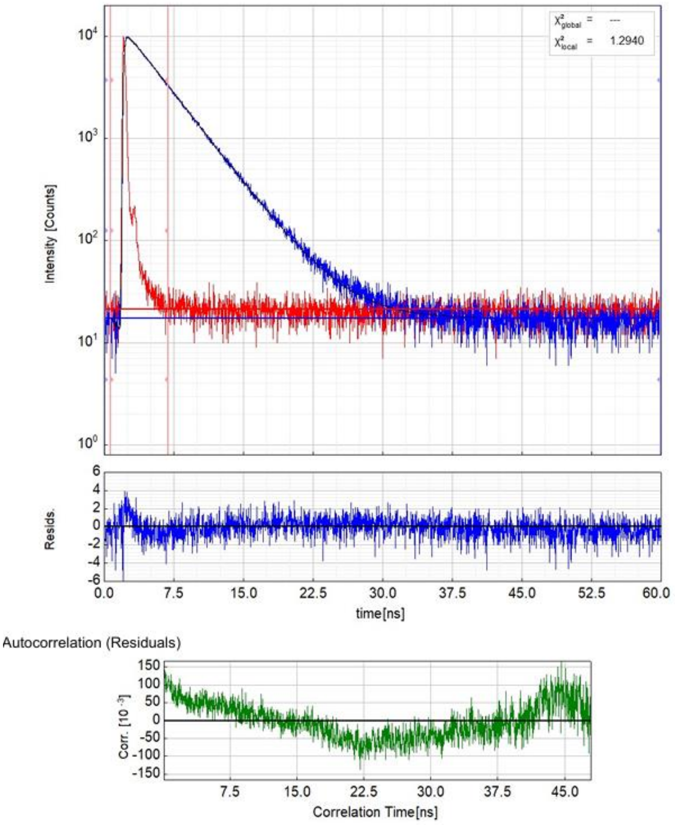

Parameter	Value	Δ	δ
A_1[kCnts/Chnl]	12.147	±0.017	0.1%
τ_1[ns]	3.6658	±0.0018	0.0%
I_1[kCnts]	1 781.1	±2.3	0.1%
$Bkgr_{Dec}$[kCnts]	0.0174	±0.0002	0.6%
$Bkgr_{IRF}$[Cnts/Chnl]	21.31	±0.35	1.6%
$Shift_{IRF}$[ps]	118.2	±1.9	1.6%
τ_{AvInt}[ns]	3.6658	±0.0018	0.0%
τ_{AvAmp}[ns]	3.6658	±0.0018	0.0%

Figure S54. Lifetime measurement of the luminescence of **2** at r. t. in CH_2Cl_2 on excitation with a 375 nm laser and detection at the emission maximum of 435 nm (τ = 3.67 ns).

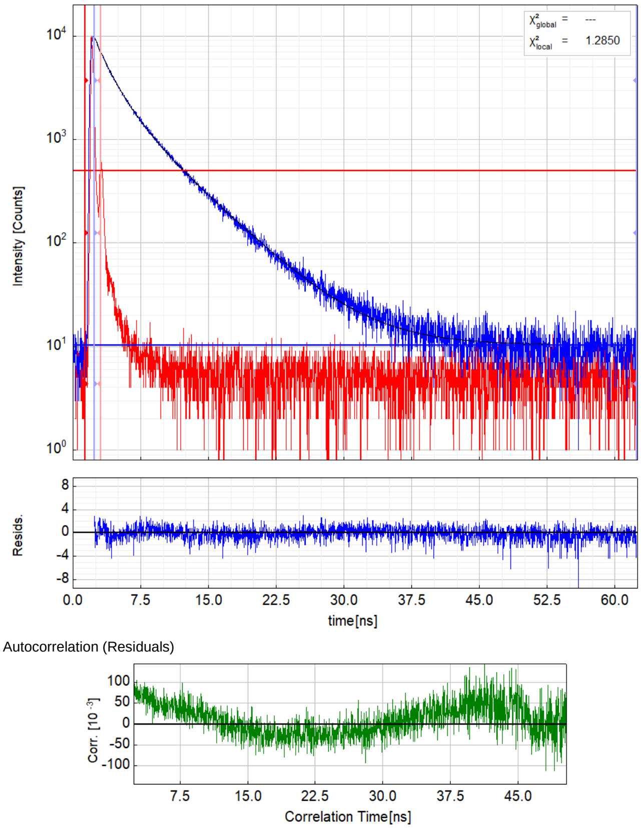

Parameter	Value	Δ	δ
A_1[kCnts/Chnl]	10.02	±0.15	1.4%
τ_1[ns]	1.726	±0.032	1.8%
I_1[kCnts]	692	±21	3.0%
A_2[kCnts/Chnl]	4.55	±0.11	2.2%
τ_2[ns]	5.191	±0.037	0.7%
I_2[kCnts]	944	±14	1.4%
$Bkgr_{Dec}$[kCnts]	0.0104	±0.0002	1.6%
$Bkgr_{IRF}$[Cnts/Chnl]	502	±16	3.2%
$Shift_{IRF}$[ps]	260	±16	5.8%
τ_{AvInt}[ns]	3.7254	±0.0052	0.1%
τ_{AvAmp}[ns]	2.808	±0.018	0.6%

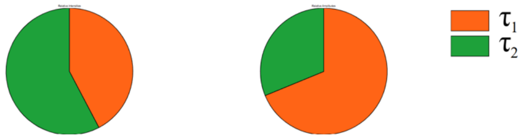

Figure S55. Lifetime measurement of the luminescence of **2** at 77 K in 2-methyl-THF on excitation with a 375 nm laser and detection at the emission maximum of 410 nm (τ = 1.73, 5.19 ns).

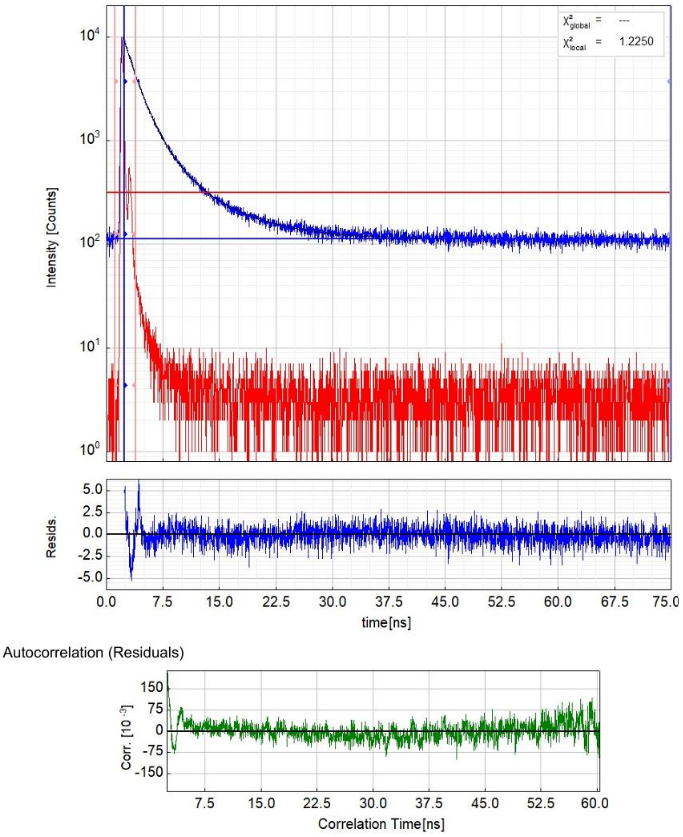

Parameter	Value	Δ	δ
A_1[kCnts/Chnl]	1.435	±0.029	2.0%
τ_1[ns]	6.440	±0.069	1.1%
I_1[kCnts]	369.5	±6.4	1.7%
A_2[kCnts/Chnl]	11.68	±0.21	1.8%
τ_2[ns]	1.895	±0.019	1.0%
I_2[kCnts]	885	±17	1.9%
$Bkgr_{Dec}$[kCnts]	0.1129	±0.0010	0.8%
$Bkgr_{IRF}$[Cnts/Chnl]	314	±12	3.8%
$Shift_{IRF}$[ps]	209	±16	7.7%
τ_{AvInt}[ns]	3.234	±0.026	0.8%
τ_{AvAmp}[ns]	2.392	±0.020	0.8%

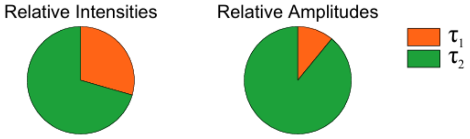

Figure S56. Lifetime measurement of the luminescence of **2** at 77 K in 2-methyl-THF on excitation with a 375 nm laser and detection at the emission maximum of 500 nm (τ = 1.90, 6.44 ns).

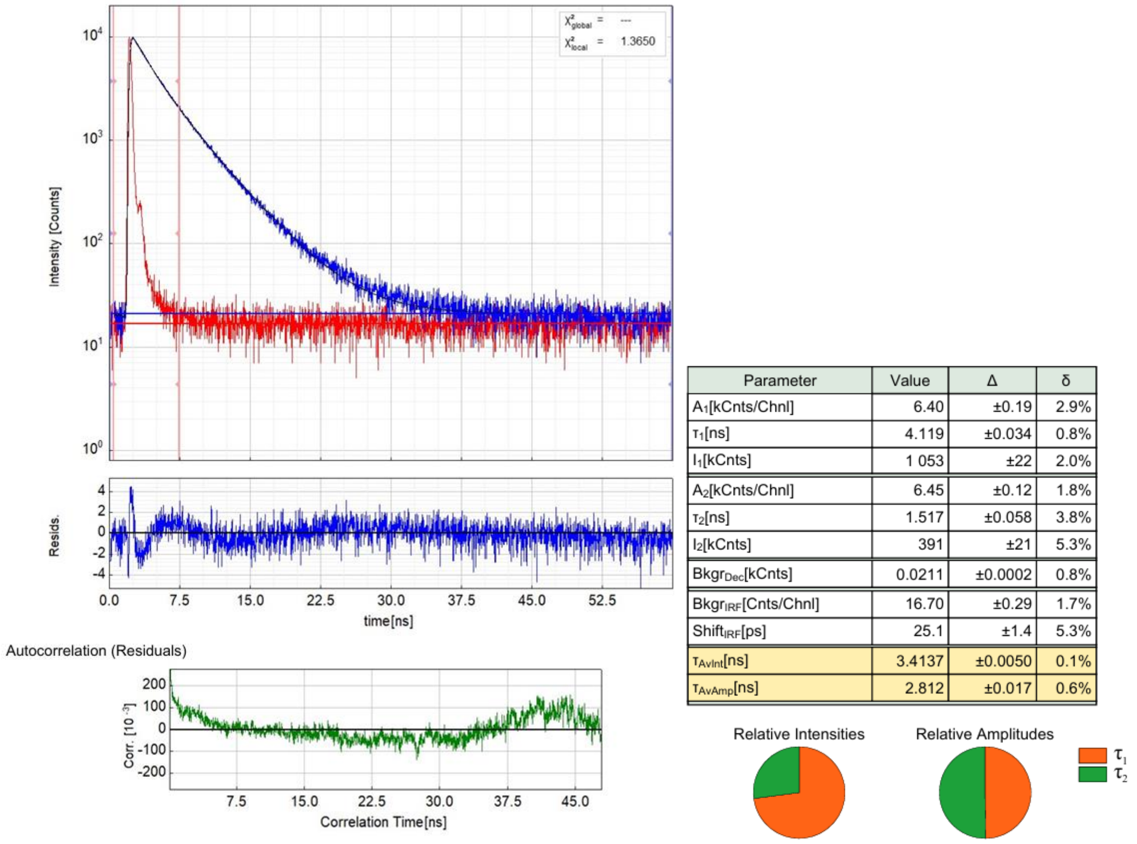

Parameter	Value	Δ	δ
A_1[kCnts/Chnl]	6.40	±0.19	2.9%
$τ_1$[ns]	4.119	±0.034	0.8%
I_1[kCnts]	1 053	±22	2.0%
A_2[kCnts/Chnl]	6.45	±0.12	1.8%
$τ_2$[ns]	1.517	±0.058	3.8%
I_2[kCnts]	391	±21	5.3%
$Bkgr_{Dec}$[kCnts]	0.0211	±0.0002	0.8%
$Bkgr_{IRF}$[Cnts/Chnl]	16.70	±0.29	1.7%
$Shift_{IRF}$[ps]	25.1	±1.4	5.3%
$τ_{AvInt}$[ns]	3.4137	±0.0050	0.1%
$τ_{AvAmp}$[ns]	2.812	±0.017	0.6%

Figure S57. Lifetime measurement of the luminescence of **3** at r. t. in CH_2Cl_2 on excitation with a 420 nm laser and detection at the emission maximum of 575 nm (τ = 1.52, 4.12 ns).

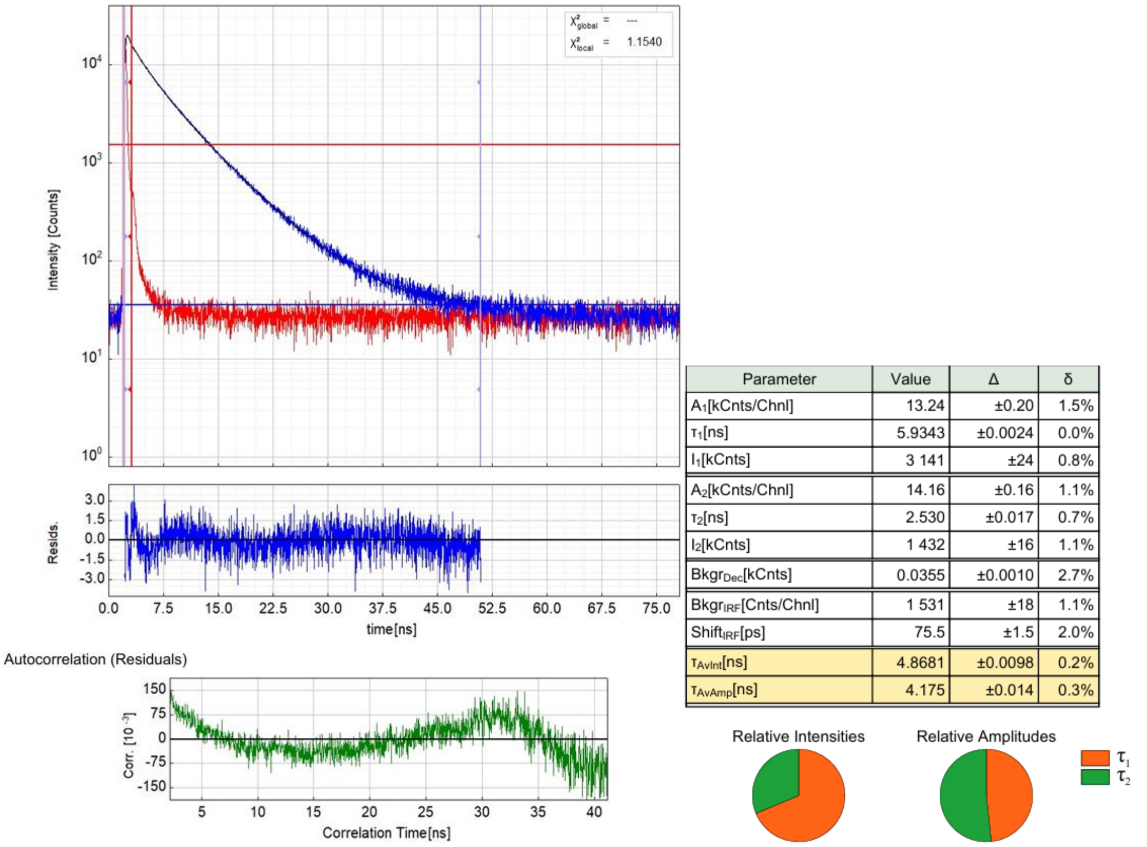

Parameter	Value	Δ	δ
A_1[kCnts/Chnl]	13.24	±0.20	1.5%
$τ_1$[ns]	5.9343	±0.0024	0.0%
I_1[kCnts]	3 141	±24	0.8%
A_2[kCnts/Chnl]	14.16	±0.16	1.1%
$τ_2$[ns]	2.530	±0.017	0.7%
I_2[kCnts]	1 432	±16	1.1%
$Bkgr_{Dec}$[kCnts]	0.0355	±0.0010	2.7%
$Bkgr_{IRF}$[Cnts/Chnl]	1 531	±18	1.1%
$Shift_{IRF}$[ps]	75.5	±1.5	2.0%
$τ_{AvInt}$[ns]	4.8681	±0.0098	0.2%
$τ_{AvAmp}$[ns]	4.175	±0.014	0.3%

Figure S58. Lifetime measurement of the luminescence of **3** at r. t. in THF on excitation with a 420 nm laser and detection at the emission maximum of 540 nm (τ = 2.53, 5.93 ns).

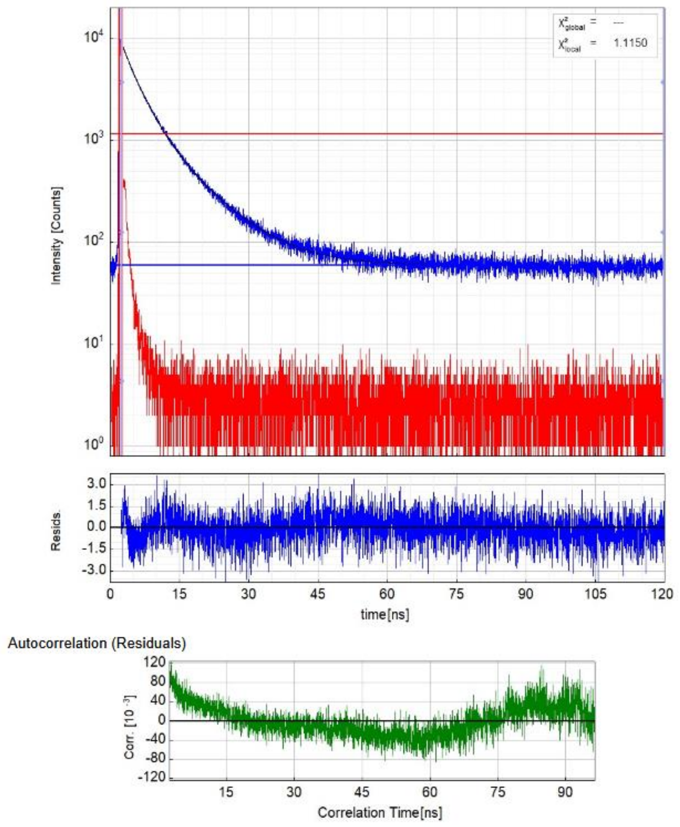

Parameter	Value	Δ	δ
A_1[kCnts/Chnl]	10.484	±0.064	0.6%
τ_1[ns]	3.1502	±0.0033	0.1%
I_1[kCnts]	1 321.1	±7.7	0.6%
A_2[kCnts/Chnl]	3.414	±0.025	0.7%
τ_2[ns]	8.8050	±0.0079	0.1%
I_2[kCnts]	1 202.4	±7.6	0.6%
$Bkgr_{Dec}$[kCnts]	0.0599	±0.0007	1.1%
$Bkgr_{IRF}$[Cnts/Chnl]	1 160.02	±0.67	0.1%
$Shift_{IRF}$[ps]	325.011	±0.025	0.0%
τ_{AvInt}[ns]	5.8446	±0.0085	0.1%
τ_{AvAmp}[ns]	4.5392	±0.0065	0.1%

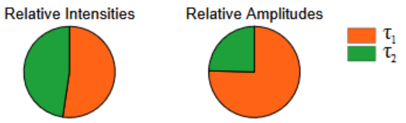

Figure S59. Lifetime measurement of the luminescence of **3** at 77 K in 2-methyl-THF on excitation with a 375 nm laser and detection at the emission maximum of 532 nm (τ = 3.15, 8.80 ns).

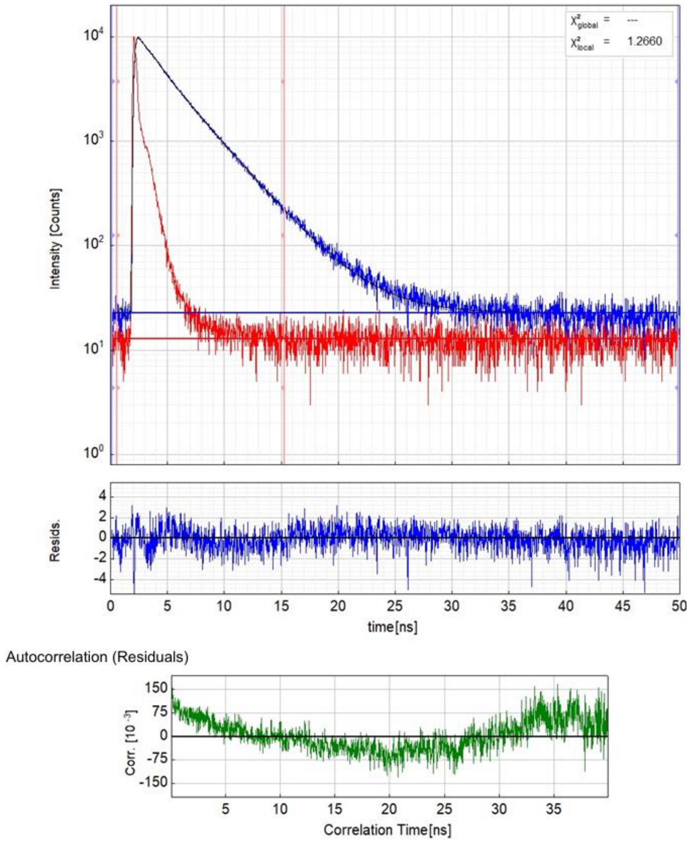

Parameter	Value	Δ	δ
A_1[kCnts/Chnl]	8.559	±0.035	0.4%
τ_1[ns]	3.385	±0.012	0.3%
I_1[kCnts]	1 158.9	±1.8	0.2%
A_2[kCnts/Chnl]	11.77	±0.19	1.6%
τ_2[ns]	0.595	±0.013	2.1%
I_2[kCnts]	279.7	±2.3	0.8%
$Bkgr_{Dec}$[kCnts]	0.0228	±0.0001	0.4%
$Bkgr_{IRF}$[Cnts/Chnl]	12.80	±0.30	2.3%
$Shift_{IRF}$[ps]	122.74	±0.96	0.8%
τ_{AvInt}[ns]	2.8424	±0.0055	0.2%
τ_{AvAmp}[ns]	1.770	±0.018	1.0%

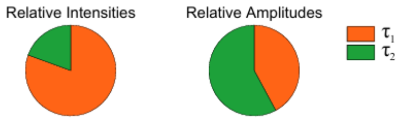

Figure S60. Lifetime measurement of the luminescence of **1** at r. t. in CH_2Cl_2 on excitation with a 375 nm laser and detection at the emission maximum of 428 nm (τ_1 = 3.39 ns, τ_2 = 0.60 ns).

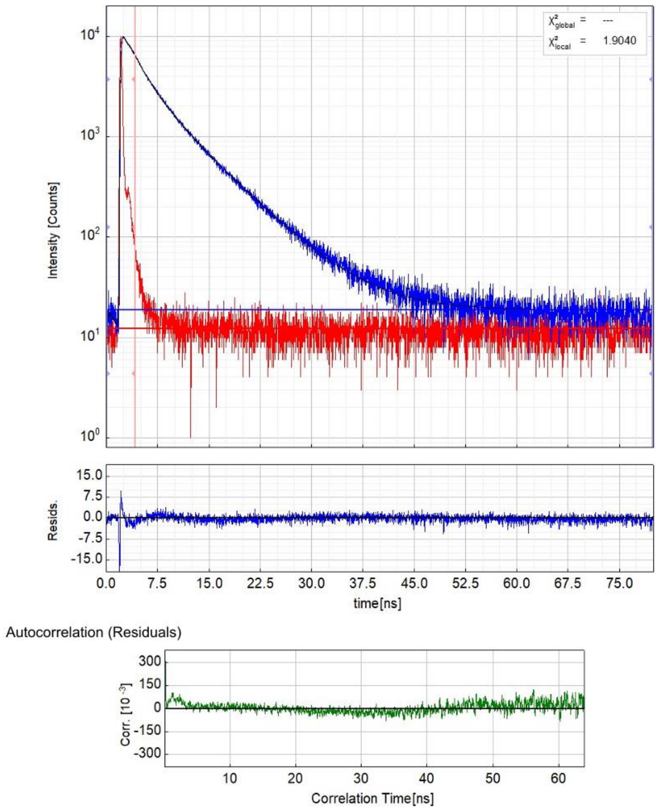

Parameter	Value	Δ	δ
A_1[kCnts/Chnl]	4.879	±0.065	1.3%
$τ_1$[ns]	6.356	±0.035	0.5%
I_1[kCnts]	1 241	±11	0.9%
A_2[kCnts/Chnl]	7.944	±0.042	0.5%
$τ_2$[ns]	1.989	±0.038	1.9%
I_2[kCnts]	632	±14	2.1%
$Bkgr_{Dec}$[kCnts]	0.0188	±0.0002	0.9%
$Bkgr_{IRF}$[Cnts/Chnl]	12.16	±0.20	1.6%
$Shift_{IRF}$[ps]	52.7	±6.2	12%
$τ_{AvInt}$[ns]	4.8817	±0.0081	0.2%
$τ_{AvAmp}$[ns]	3.651	±0.020	0.5%

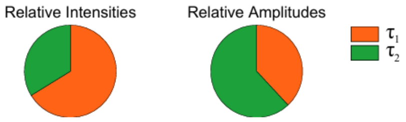

Figure S61. Lifetime measurement of the luminescence of **1** at r. t. in CH_2Cl_2 on excitation with a 375 nm laser and detection at the emission maximum of 574 nm ($τ_1$ = 1.99 ns, $τ_2$ = 6.36 ns).

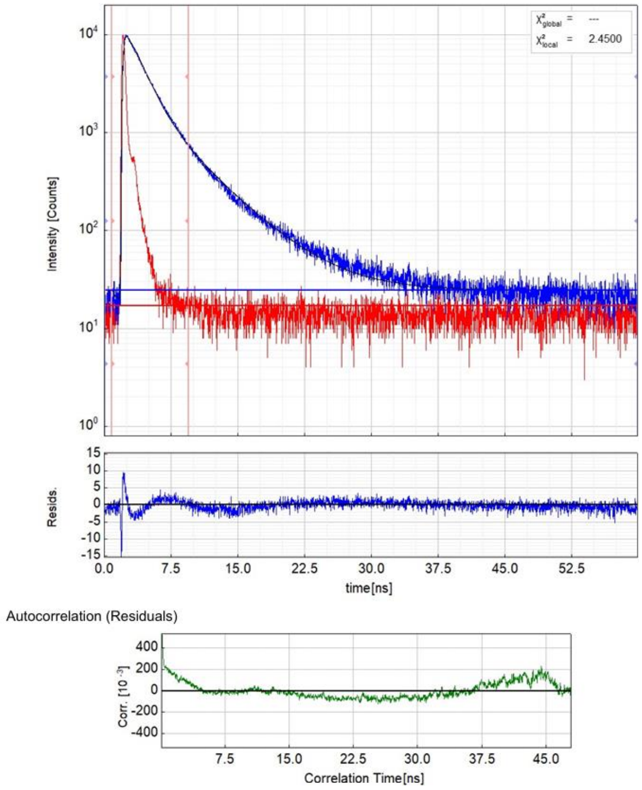

Parameter	Value	Δ	δ
A_1[kCnts/Chnl]	2.79	±0.17	6.0%
$τ_1$[ns]	4.799	±0.091	1.9%
I_1[kCnts]	535	±23	4.3%
A_2[kCnts/Chnl]	12.376	±0.078	0.6%
$τ_2$[ns]	1.430	±0.047	3.2%
I_2[kCnts]	708	±17	2.3%
$Bkgr_{Dec}$[kCnts]	0.0245	±0.0004	1.5%
$Bkgr_{IRF}$[Cnts/Chnl]	17.0	±1.3	7.1%
$Shift_{IRF}$[ps]	-0.9	±2.0	223%
$τ_{AvInt}$[ns]	2.880	±0.016	0.5%
$τ_{AvAmp}$[ns]	2.049	±0.026	1.2%

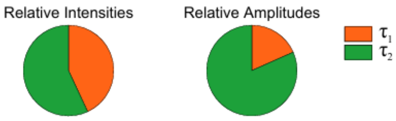

Figure S62. Lifetime measurement of the luminescence of **1** at r. t. in CH_2Cl_2 on excitation with a 420 nm laser and detection at the emission maximum of 608 nm ($τ_1$ = 4.80 ns, $τ_2$ = 1.43 ns).

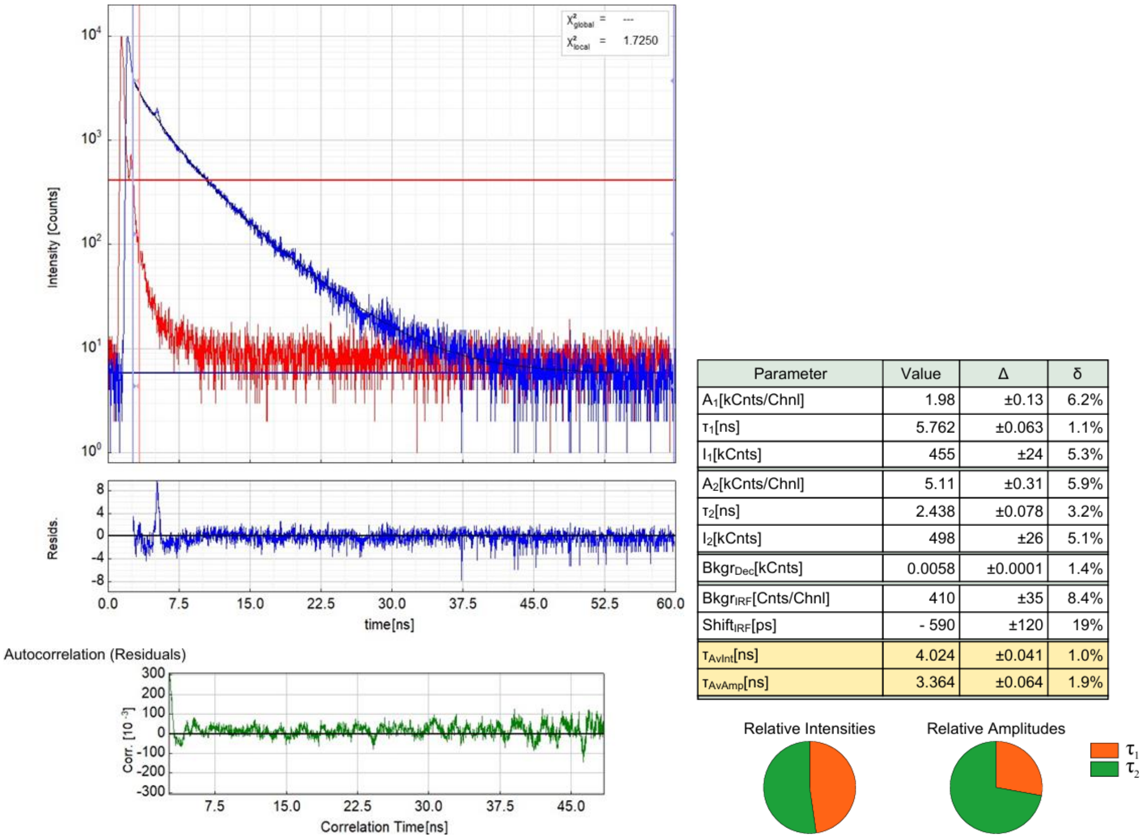

Parameter	Value	Δ	δ
A_1[kCnts/Chnl]	1.98	±0.13	6.2%
τ_1[ns]	5.762	±0.063	1.1%
I_1[kCnts]	455	±24	5.3%
A_2[kCnts/Chnl]	5.11	±0.31	5.9%
τ_2[ns]	2.438	±0.078	3.2%
I_2[kCnts]	498	±26	5.1%
$Bkgr_{Dec}$[kCnts]	0.0058	±0.0001	1.4%
$Bkgr_{IRF}$[Cnts/Chnl]	410	±35	8.4%
$Shift_{IRF}$[ps]	- 590	±120	19%
τ_{AvInt}[ns]	4.024	±0.041	1.0%
τ_{AvAmp}[ns]	3.364	±0.064	1.9%

Figure S63. Lifetime measurement of the fluorescence of **1** at 77 K in 2-methyl-THF on excitation with a 375 nm laser and detection at 390 nm (τ_1 = 5.76 ns, τ_2 = 2.44 ns).

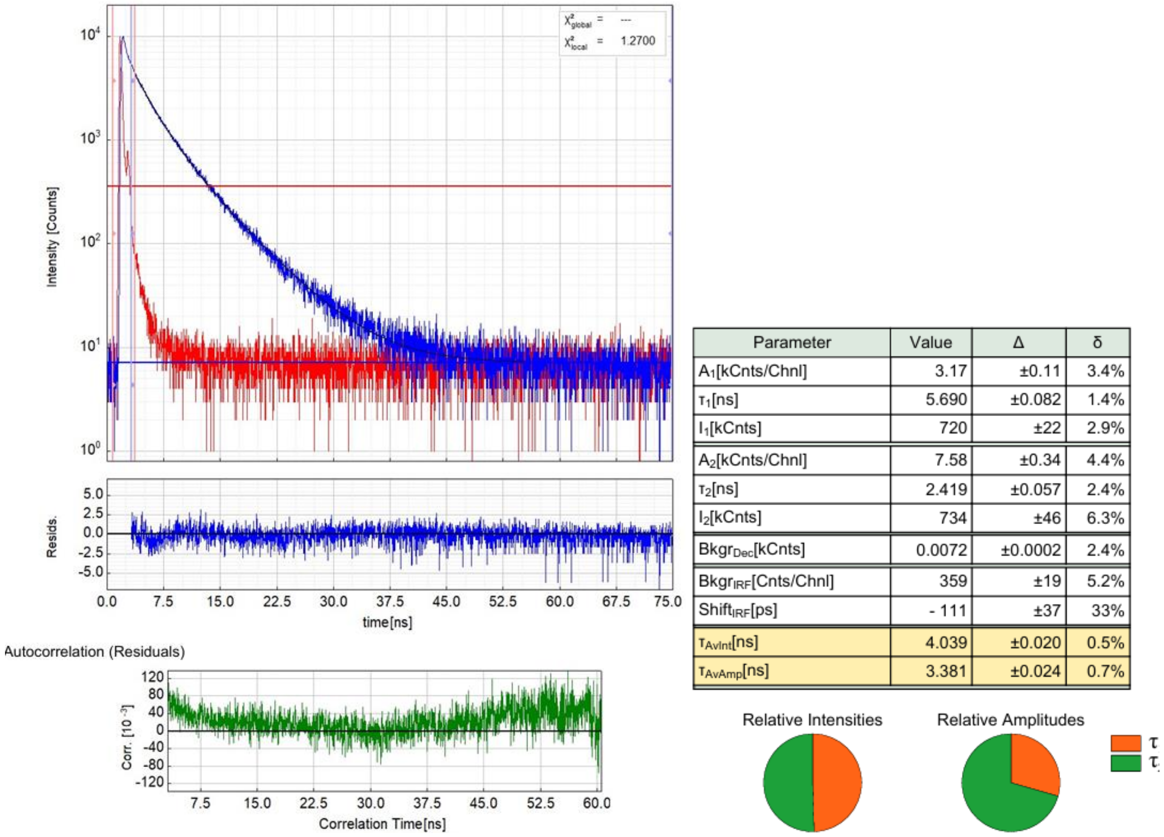

Parameter	Value	Δ	δ
A_1[kCnts/Chnl]	3.17	±0.11	3.4%
τ_1[ns]	5.690	±0.082	1.4%
I_1[kCnts]	720	±22	2.9%
A_2[kCnts/Chnl]	7.58	±0.34	4.4%
τ_2[ns]	2.419	±0.057	2.4%
I_2[kCnts]	734	±46	6.3%
$Bkgr_{Dec}$[kCnts]	0.0072	±0.0002	2.4%
$Bkgr_{IRF}$[Cnts/Chnl]	359	±19	5.2%
$Shift_{IRF}$[ps]	- 111	±37	33%
τ_{AvInt}[ns]	4.039	±0.020	0.5%
τ_{AvAmp}[ns]	3.381	±0.024	0.7%

Figure S64. Lifetime measurement of the fluorescence of **1** at 77 K in 2-methyl-THF on excitation with a 375 nm laser and detection at 410 nm (τ_1 = 5.69 ns, τ_2 = 2.41 ns).

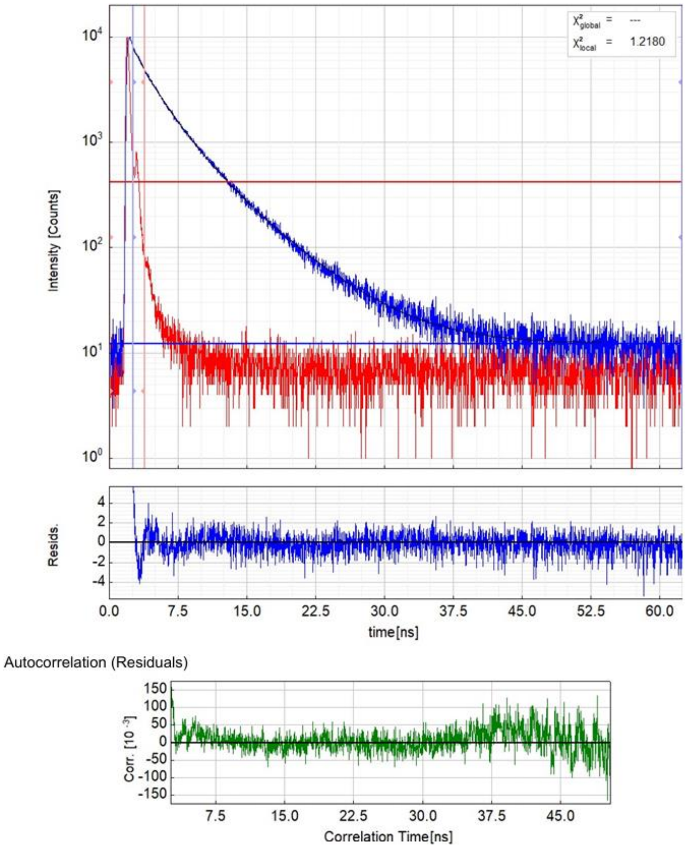

Parameter	Value	Δ	δ
A_1[kCnts/Chnl]	10.12	±0.26	2.5%
τ_1[ns]	2.487	±0.031	1.2%
I_1[kCnts]	1 007	±28	2.8%
A_2[kCnts/Chnl]	3.465	±0.066	1.9%
τ_2[ns]	5.748	±0.053	0.9%
I_2[kCnts]	797	±21	2.5%
$Bkgr_{Dec}$[kCnts]	0.0123	±0.0001	0.8%
$Bkgr_{IRF}$[Cnts/Chnl]	421	±19	4.4%
$Shift_{IRF}$[ps]	96	±29	30%
τ_{AvInt}[ns]	3.9274	±0.0100	0.3%
τ_{AvAmp}[ns]	3.319	±0.013	0.4%

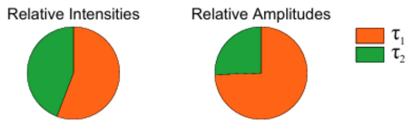

Figure S65. Lifetime measurement of the fluorescence of **1** at 77 K in 2-methyl-THF on excitation with a 375 nm laser and detection at 440 nm (τ_1 = 5.75 ns, τ_2 = 2.49 ns).

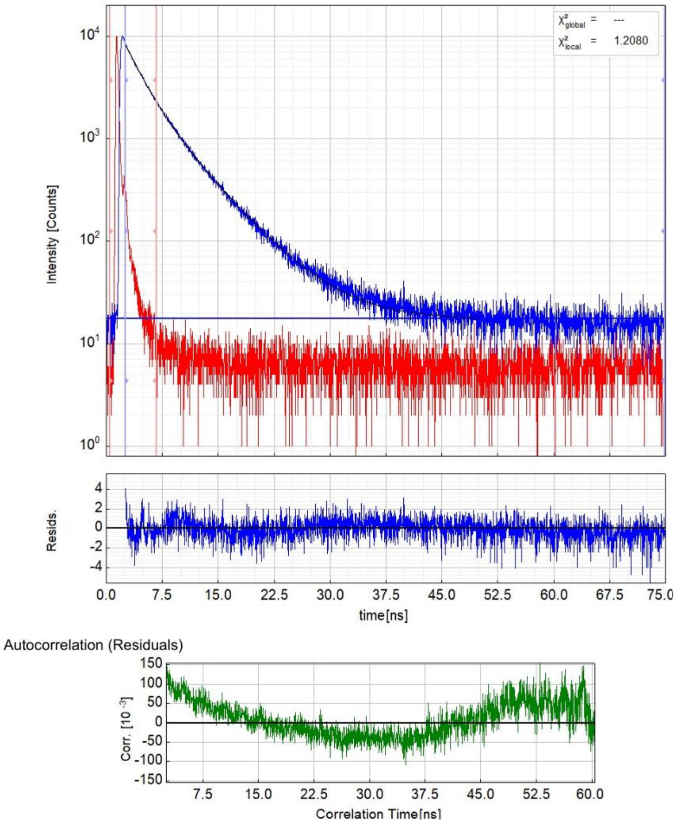

Parameter	Value	Δ	δ
A_1[kCnts/Chnl]	4.281	±0.065	1.5%
τ_1[ns]	5.369	±0.029	0.5%
I_1[kCnts]	920	±11	1.1%
A_2[kCnts/Chnl]	9.98	±0.15	1.5%
τ_2[ns]	1.974	±0.054	2.7%
I_2[kCnts]	787.2	±8.8	1.1%
$Bkgr_{Dec}$[kCnts]	0.0176	±0.0002	0.9%
$Bkgr_{IRF}$[Cnts/Chnl]	-6.6	±3.9	59%
$Shift_{IRF}$[ps]	- 425.2	±1.3	0.3%
τ_{AvInt}[ns]	3.803	±0.020	0.5%
τ_{AvAmp}[ns]	2.994	±0.045	1.5%

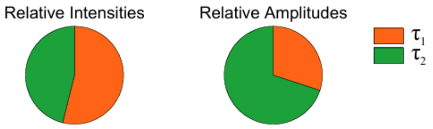

Figure S66. Lifetime measurement of the fluorescence of **1** at 77 K in 2-methyl-THF on excitation with a 375 nm laser and detection at 450 nm (τ_1 = 5.37 ns, τ_2 = 1.97 ns).

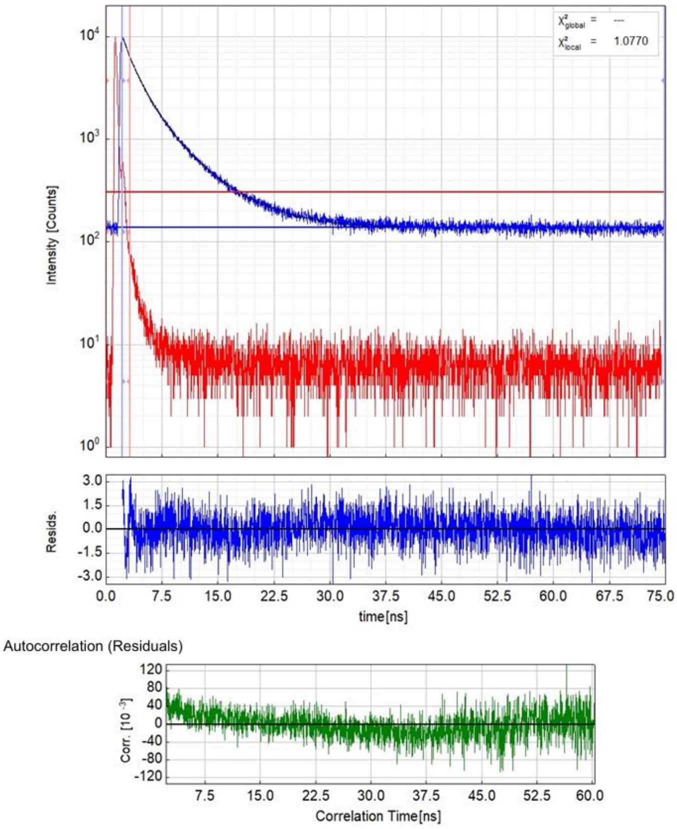

Parameter	Value	Δ	δ
A_1[kCnts/Chnl]	4.207	±0.022	0.5%
τ_1[ns]	5.5512	±0.0017	0.0%
I_1[kCnts]	934.1	±9.3	1.0%
A_2[kCnts/Chnl]	13.17	±0.17	1.3%
τ_2[ns]	1.9335	±0.0006	0.0%
I_2[kCnts]	1 019	±13	1.2%
$Bkgr_{Dec}$[kCnts]	0.1379	±0.0004	0.2%
$Bkgr_{IRF}$[Cnts/Chnl]	305.05	±0.28	0.1%
$Shift_{IRF}$[ps]	- 562.4948	±0.0053	0.0%
τ_{AvInt}[ns]	3.665	±0.016	0.4%
τ_{AvAmp}[ns]	2.810	±0.012	0.4%

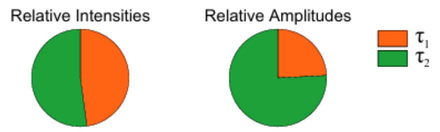

Figure S67. Lifetime measurement of the fluorescence of **1** at 77 K in 2-methyl-THF on excitation with a 375 nm laser and detection at 470 nm (τ_1 = 5.55 ns, τ_2 = 1.93 ns).

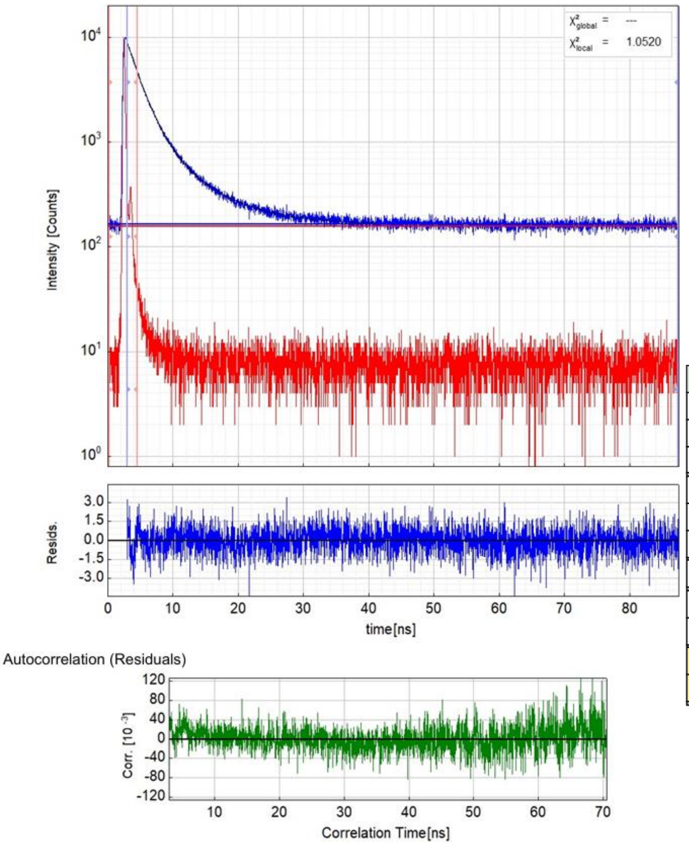

Parameter	Value	Δ	δ
A_1[kCnts/Chnl]	11.13	±0.12	1.0%
τ_1[ns]	2.087	±0.030	1.4%
I_1[kCnts]	929	±20	2.1%
A_2[kCnts/Chnl]	1.718	±0.077	4.4%
τ_2[ns]	6.77	±0.13	1.9%
I_2[kCnts]	465	±12	2.6%
$Bkgr_{Dec}$[kCnts]	0.1637	±0.0003	0.2%
$Bkgr_{IRF}$[Cnts/Chnl]	157.6	±8.6	5.4%
$Shift_{IRF}$[ps]	182	±20	11%
τ_{AvInt}[ns]	3.647	±0.030	0.8%
τ_{AvAmp}[ns]	2.712	±0.018	0.6%

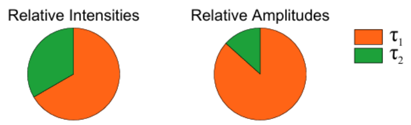

Figure S68. Lifetime measurement of the fluorescence of **1** at 77 K in 2-methyl-THF on excitation with a 485 nm laser and detection at 500 nm (τ_1 = 6.77 ns, τ_2 = 2.09 ns).

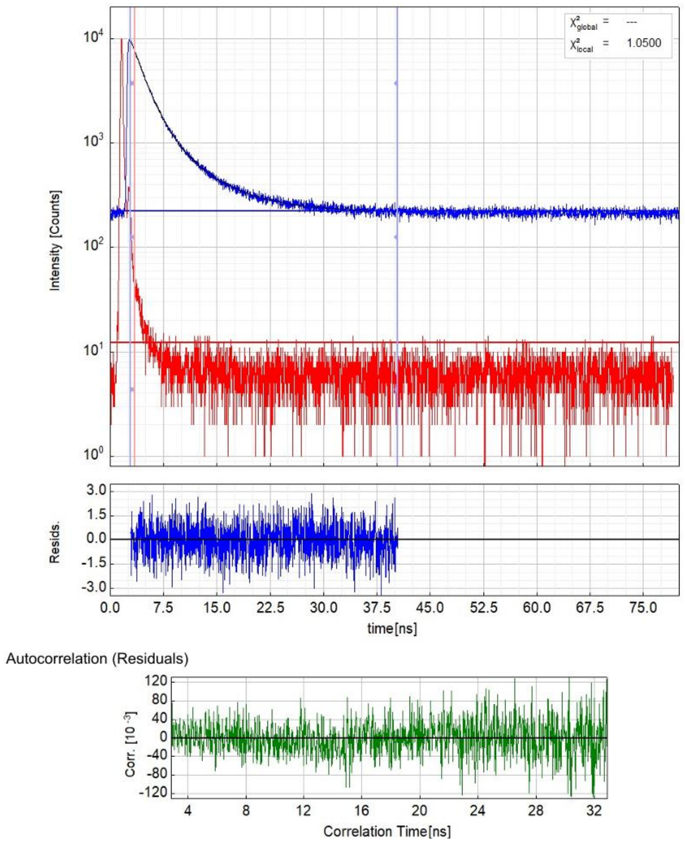

Parameter	Value	Δ	δ
A_1[kCnts/Chnl]	2.161	±0.084	3.9%
τ_1[ns]	5.86	±0.12	1.9%
I_1[kCnts]	507	±11	2.1%
A_2[kCnts/Chnl]	14.89	±0.27	1.8%
τ_2[ns]	1.879	±0.023	1.2%
I_2[kCnts]	1 119	±34	3.0%
$Bkgr_{Dec}$[kCnts]	0.2219	±0.0009	0.4%
$Bkgr_{IRF}$[Cnts/Chnl]	12.3	±8.5	69%
$Shift_{IRF}$[ps]	- 790	±26	3.2%
τ_{AvInt}[ns]	3.119	±0.021	0.6%
τ_{AvAmp}[ns]	2.384	±0.014	0.6%

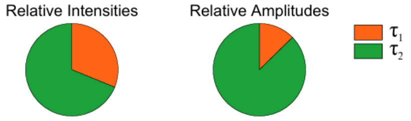

Figure S69. Lifetime measurement of the fluorescence of **1** at 77 K in 2-methyl-THF on excitation with a 485 nm laser and detection at 550 nm (τ_1 = 5.86 ns, τ_2 = 1.88 ns).

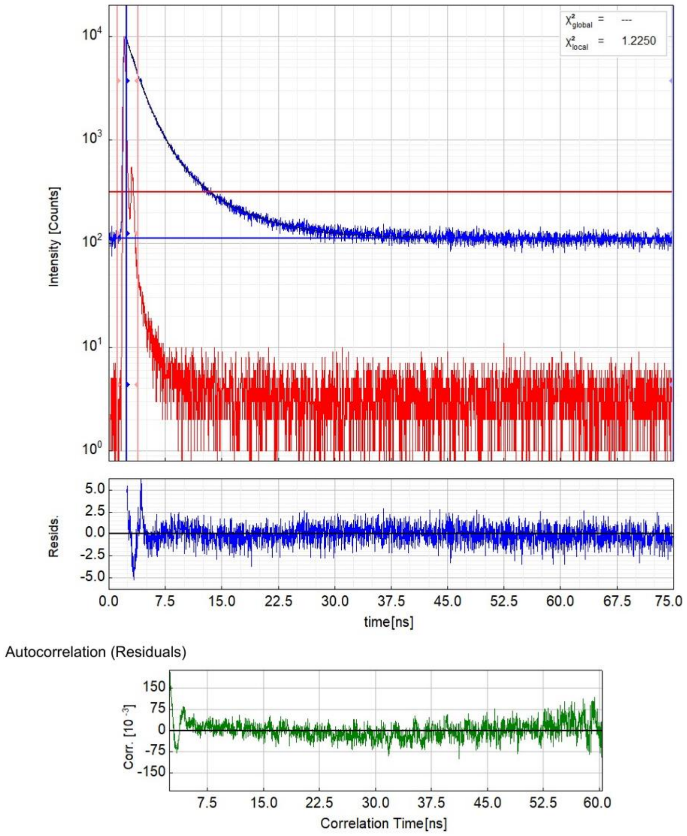

Parameter	Value	Δ	δ
A_1[kCnts/Chnl]	1.435	±0.029	2.0%
τ_1[ns]	6.440	±0.069	1.1%
I_1[kCnts]	369.5	±6.4	1.7%
A_2[kCnts/Chnl]	11.68	±0.21	1.8%
τ_2[ns]	1.895	±0.019	1.0%
I_2[kCnts]	885	±17	1.9%
$Bkgr_{Dec}$[kCnts]	0.1129	±0.0010	0.8%
$Bkgr_{IRF}$[Cnts/Chnl]	314	±12	3.8%
$Shift_{IRF}$[ps]	209	±16	7.7%
τ_{AvInt}[ns]	3.234	±0.026	0.8%
τ_{AvAmp}[ns]	2.392	±0.020	0.8%

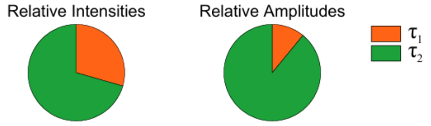

Figure S70. Lifetime measurement of the fluorescence of **1** at 77 K in 2-methyl-THF on excitation with a 485 nm laser and detection at 650 nm (τ_1 = 6.44 ns, τ_2 = 1.90 ns).

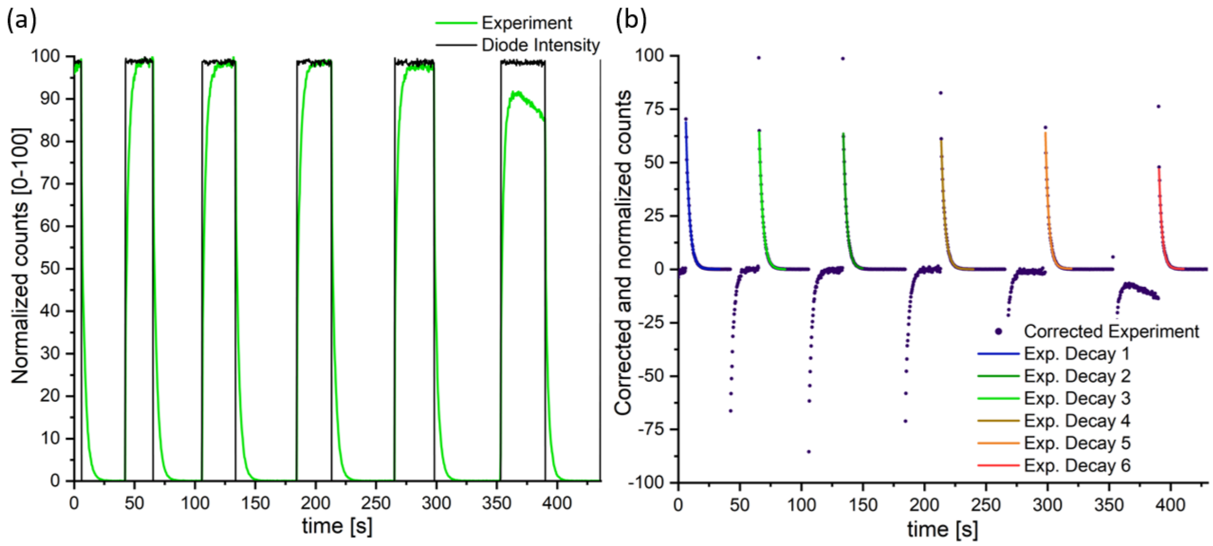

Model	Exponential decay $A + Intensity_0 \bullet exp(-\frac{time}{\tau})$						
**Experimental Decay**	**1**	**2**	**3**	**4**	**5**	**6**	**Ø**
τ	2.914 ± 0.024	2.851 ± 0.0299	3.008 ± 0.014	3.052 ± 0.016	2.737 ± 0.030	2.550 ± 0.022	**2.852 ± 0.369**
R^2	0.999	0.999	0.999	0.999	0.998	0.999	**0.999**

Figure S71. Steady-state lifetime measurements of compound **2** at 77 K in 2-methyl-THF by manually triggering several decays over a period of several minutes by opening and closing the He-diode shutter. The excitation wavelength was set to 375 nm and the emitted light was detected at 500 nm. (a) The experimental decays are shown in green and the intensity of the power diode is shown in black. (b) The intensity of the He-diode was subtracted from the experimental outcomes to yield the corrected spectra. The decays are fitted using exponential decay functions of the type: Corrected Intensity $I_c = I_0 + A \cdot exp(-t/\tau)$, with I_c = intensity corrected for the He-diode, I_0 initial intensity, τ = averaged lifetime of the green phosphorescence as given in the table. The error is the median of the differences of the decay functions from the calculated lifetime.

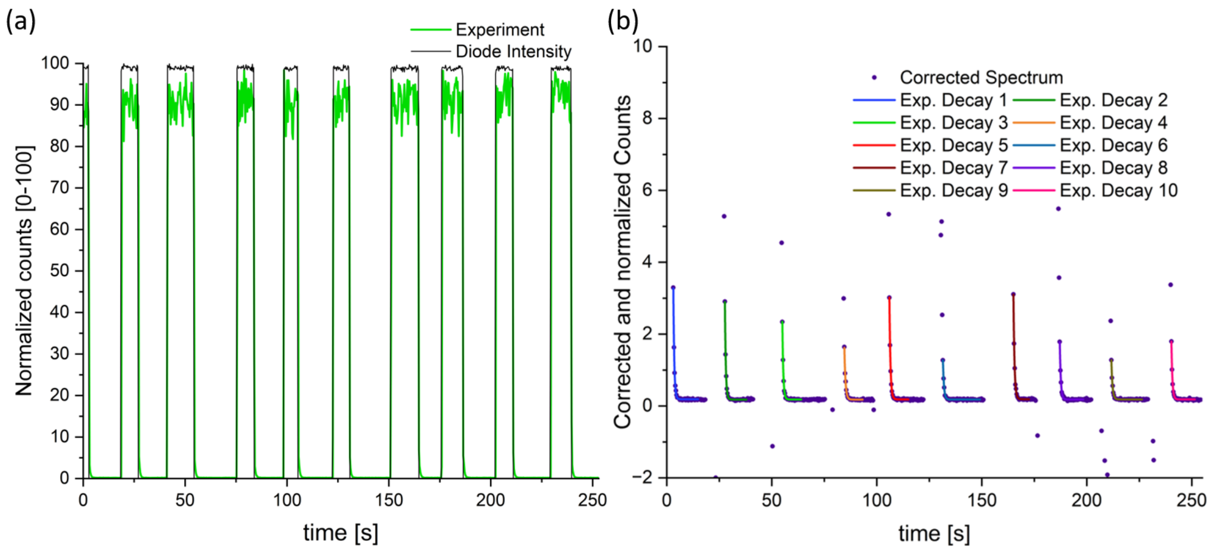

**Model**	**Exponential decay** $A + Intensity_0 \bullet exp(-\frac{time - time_0}{\tau})$									
**Exp. Decay**	**1**	**2**	**3**	**4**	**5**	**6**	**7**	**8**	**9**	**10**	**Ø**
τ	0.432 ± 0.007	0.423± 0.005	0.444± 0.008	0.538± 0.002	0.488± 0.005	0.496± 0.014	0.500± 0.006	0.489± 0.001	0.545± 0.011	0.471± 0.009	**0.483± 0.083**
R^2	0.998	0.998	0.998	0.995	0.999	0.990	0.996	0.999	0.995	0.997	**0.997**

Figure S72. Steady-state lifetime measurements of compound **3** at 77 K in 2-methyl-THF by manually triggering several decays over a period of several minutes by opening and closing the He-diode shutter. The excitation wavelength was set to 430 nm and the emitted light was detected at 532 nm. (a) The experimental decays are shown in green and the intensity of the power diode is shown in black. (b) The intensity of the He-diode was subtracted from the experimental outcomes to yield the corrected spectra. The decays are fitted using exponential decay functions of the type: Corrected Intensity $I_c = I_0 + A\cdot exp(-t/\tau)$, with I_c = intensity corrected for the He-diode, I_0 initial intensity, τ = averaged lifetime of the green phosphorescence as given in the table. The error is the median of the differences of the decay functions from the calculated lifetime.

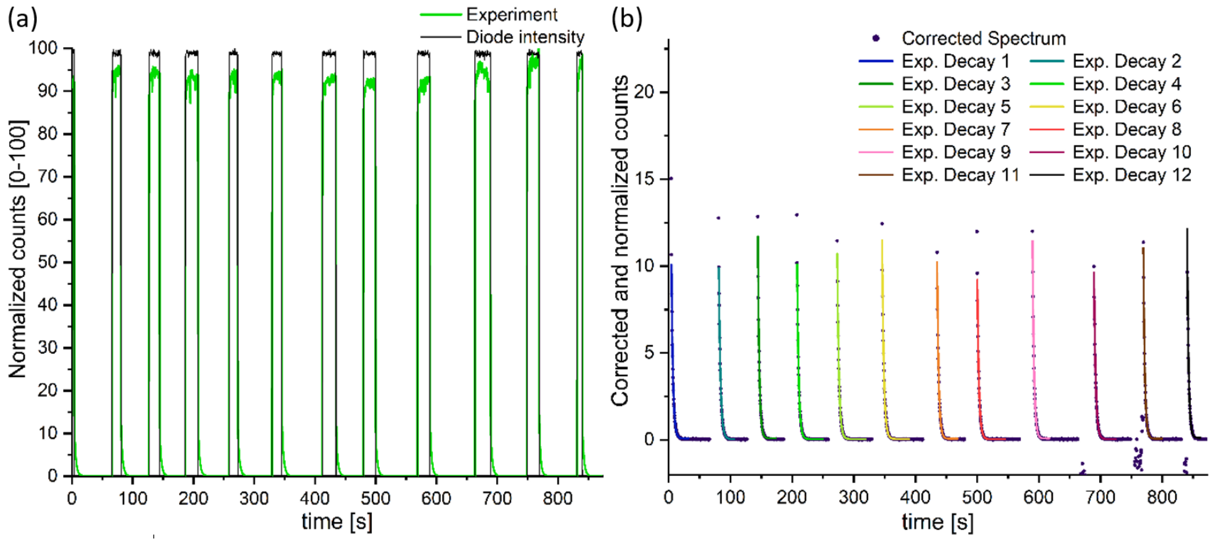

**Model**	**Exponential decay** $A + Intensity_0 \bullet exp(-\frac{time - time_0}{\tau})$												
**Exp. Decay**	**1**	**2**	**3**	**4**	**5**	**6**	**7**	**8**	**9**	**10**	**11**	**12**	**Ø**
τ	2.539 ± 0.013	2.430 ± 0.025	2.460 ± 0.027	2.685 ± 0.021	2.306 ± 0.044	2.432 ± 0.029	2.738 ± 0.015	2.613 ± 0.025	2.313 ± 0.015	2.579 ± 0.018	2.665 ± 0.032	2.239 ± 0.055	**2.500 ± 0.163**
R^2	0.999	0.998	0.998	0.999	0.993	0.997	1.000	0.997	0.999	0.998	0.996	0.992	**0.998**

Figure S73. Steady-state lifetime measurements of compound **1** at 77 K in 2-methyl-THF by manually triggering several decays over a period of several minutes by opening and closing the He-diode shutter. The excitation wavelength was set to 320 nm with detection at 500 nm. (a) The experimental decays are shown in green and the intensity of the power diode is shown in black. (b) The intensity of the He-diode was subtracted from the experimental spectra to yield the corrected spectra. The decays are fitted using exponential decay functions of the type: Corrected Intensity $I_c = I_0 + A \cdot exp(-t/\tau)$, with I_c = intensity corrected for the He-diode, I_0 initial intensity, τ = averaged lifetime of the green phosphorescence as given in the table. The error is the median of the differences of the decay functions from the calculated lifetime.

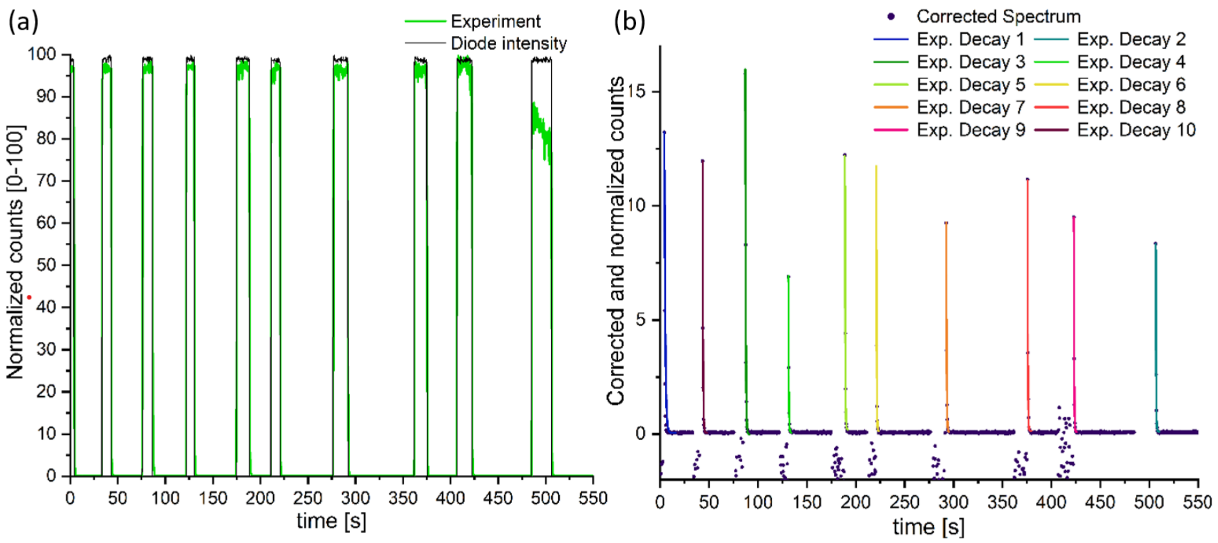

Model	Exponential decay $A + Intensity_0 \bullet exp(-\frac{time - time_0}{\tau})$									
**Exp. Decay**	**1**	**2**	**3**	**4**	**5**	**6**	**7**	**8**	**9**	**10**	**Ø**
τ	0.396 ± 0.002	0.463± 0.119	0.486± 0.000	0.384± 0.000	0.362± 0.000	0.310± 0.005	0.372± 0.010	0.325± 0.010	0.341± 0.004	0.313± 0.008	**0.375± 0.045**
R^2	1.000	0.986	0.995	0.994	0.998	0.999	0.999	0.999	1.000	0.999	**0.997**

Figure S74. Steady-state lifetime measurements of **1** at 77 K in 2-methyl-THF by manually triggering several decays over a period of several minutes by opening and closing the He-diode shutter. The excitation wavelength was set to 480 nm with detection at 550 nm. (a) The experimental decays are shown in green and the intensity of the power diode is shown in black. (b) The intensity of the He-diode was subtracted from the experimental spectra to yield the corrected spectra. The decays are fitted using exponential decay functions of the type: Corrected Intensity $I_c = I_0 + A \cdot exp(-t/\tau)$, with I_c = intensity corrected for the He-diode, I_0 initial intensity, τ = averaged lifetime of the green phosphorescence as given in the table. The error is the median of the differences of the decay functions from the calculated lifetime.

Afterglowof2.mp4

Video S1 Visual impression of the afterglow of **2** at 77 K in 2-methyl-THF.

Afterglowof3.mp4

Video S2 Visual impression of the afterglow of **3** at 77 K in 2-methyl-THF.

Afterglowof1_1.mp4 Afterglowof1_2.mp4

Video S3 Visual impression of the afterglow of **1** at 77 K in 2-methyl-THF.

Afterglowof1_solvents.mp4

Video S4 Visual impression of the afterglow of **1** at 77 K in 2-methyl-THF and in dichloromethane.

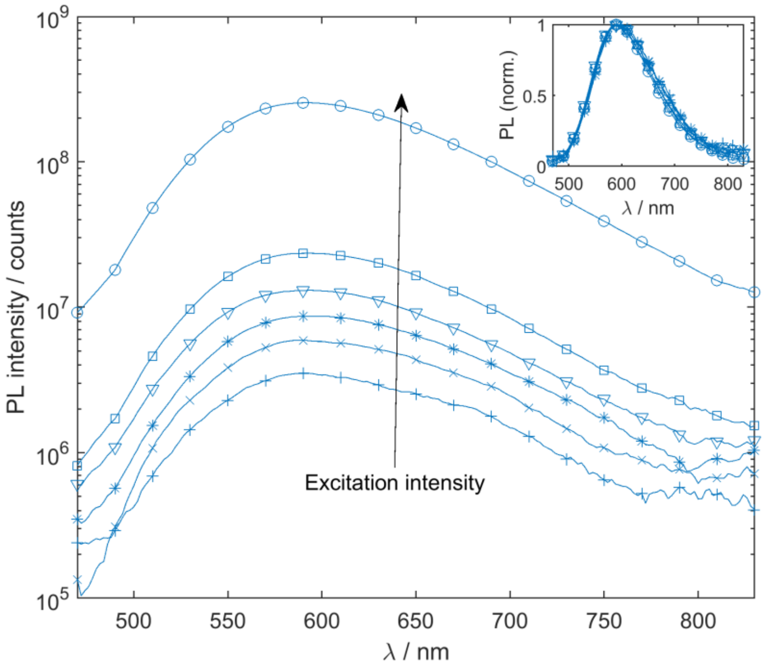

Figure S75. Excitation intensity-dependence of the PL measured on a film of **1** with a typical OLED configuration (ITO/PEDOT:PSS/EML(**1**)). Compound **1** was spin-coated onto a glass coated with ITO/PEDOT:PSS.

Scanning tunneling microscopy experiments

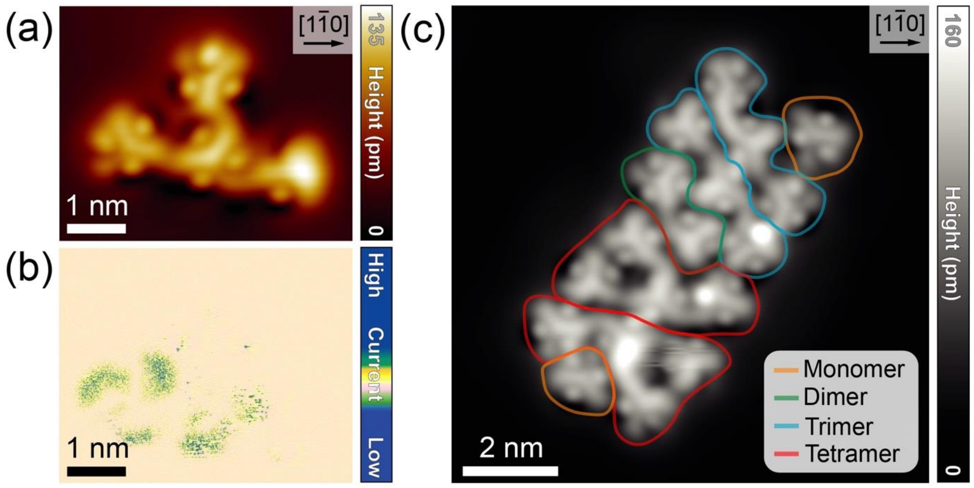

Figure S76. (a) STM topography of compound **1** on Ag(111). Scanning parameters: 5×4 nm^2, I = 50 pA, U = 10 mV, T = 3.8 K. ^{Et}**TAT** units constituting the molecular compound show an STM appearance similar to the free-standing ^{Et}**TATs**.[59,107] (b) Tunneling current image revealing fluctuations due to adsorption configuration switching.[59,107] Scanning parameters: 5×4 nm^2, I = 500 pA, U = 200 mV, T = 3.8 K. (c) STM topography of a molecular island consisting of two TAT monomers, a dimer, two trimers and two pristine tetramers **1**. Different types of molecules are highlighted by different colors. Scanning parameters: 10×10 nm^2, I = 50 pA, U = 10 mV, T = 4 K.

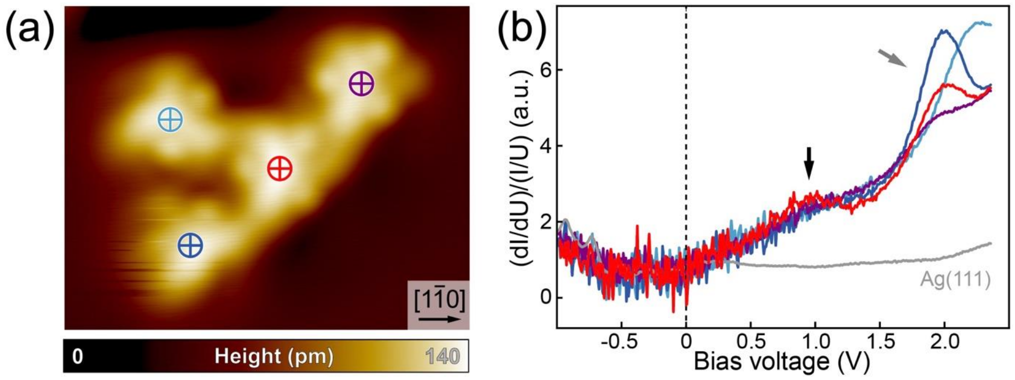

Figure S77. (a) STM topography of intact tetramer **1** on the Ag(111) surface. Scanning parameters: 5×4nm^2, I = 100 pA, U = 10 mV, T = 3.8 K. (b) Typical normalized scanning tunnelling (dI/dU)(I/U) spectra recorded on top of all four ^{Et}**TAT** units (color) as well as on the Ag(111) surface (grey). The tip positions of the corresponding measurements are marked in (a) by colored, encircled crosses. All ^{Et}**TAT** units show a pronounced spectral feature at about 2 eV (grey arrow). The central ^{Et}**TAT** unit shows an additional feature at about 1 eV (black arrow). Spectroscopy parameters: U_{set} = 2.5 V, I = 10 pA, T = 3.8 K.

OLED performances

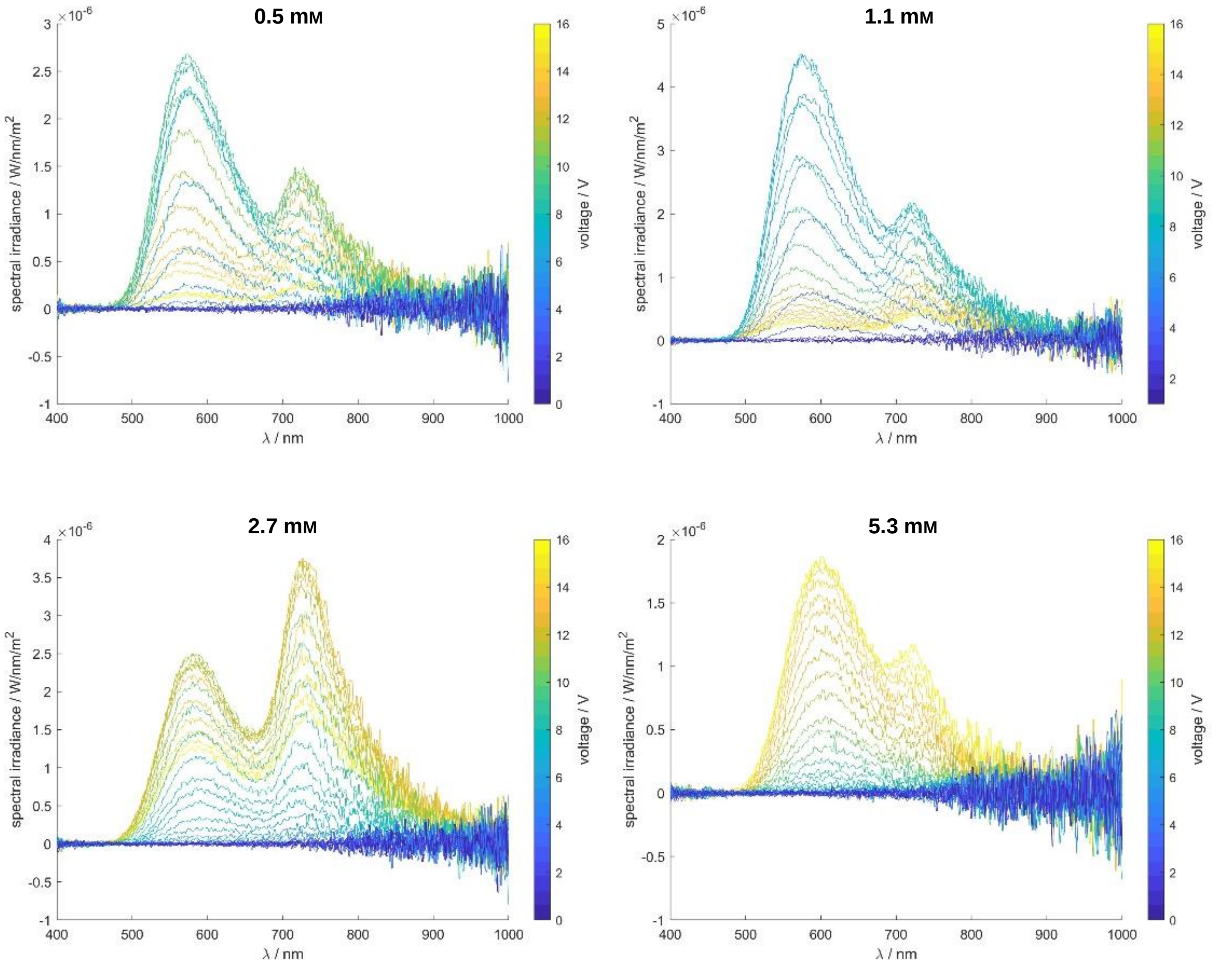

Figure S78. Bias-dependent emission spectra of **1** recorded during voltage sweeps of OLEDs (glass/ITO/PEDOT:PSS/**1**/PO-T2T/LiF/Al) at different driving voltages with EML films spin-coated from solutions in THF and with different concentrations of **1**.

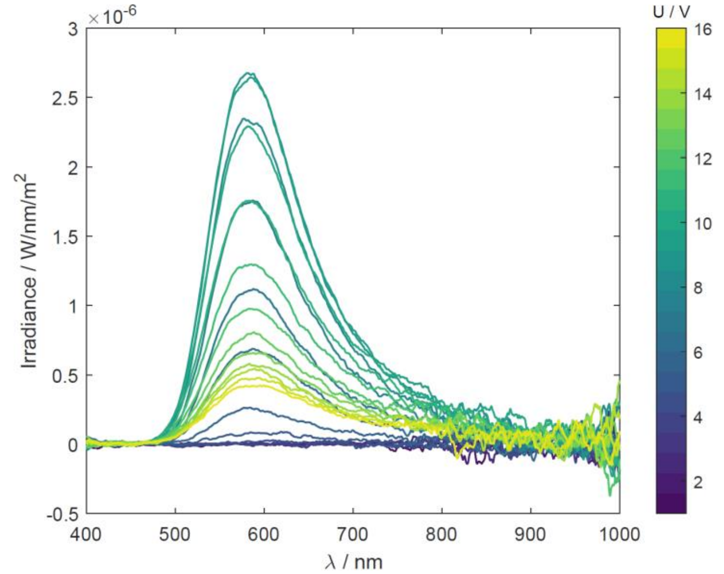

Figure S79. Bias-dependent emission spectra of compounds **2** and **3** recorded during voltage sweeps of an OLED (glass/ITO/PEDOT:PSS/**2**+**3**(3:1)/PO-T2T/LiF/Al), employing a 3:1 mixture of **2** and **3** as EML spin-coated from a solution in THF with a concentration of 2 mg/mL.

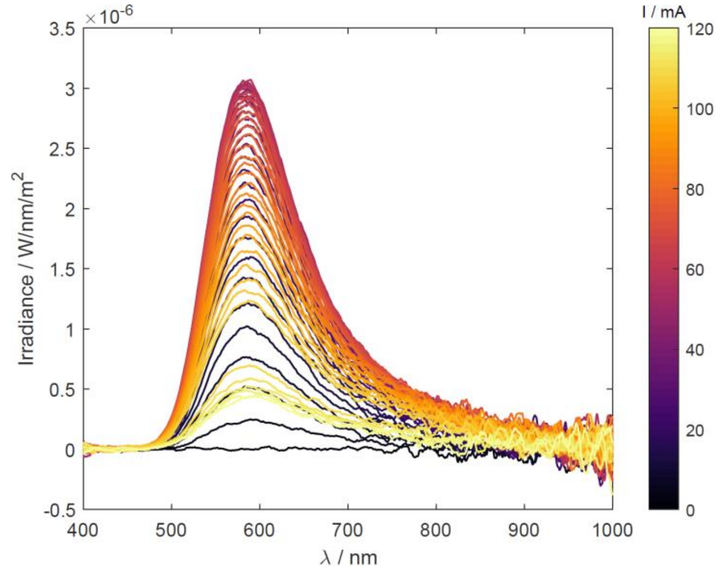

Figure S80. Emission spectra in dependence of the current flowing through an OLED (glass/ITO/PEDOT:PSS/**2**+**3**(3:1)/PO-T2T/LiF/Al), employing a 3:1 mixture of **2** and **3** as EML spin-coated from a solution in THF with a concentration of 2 mg/mL.

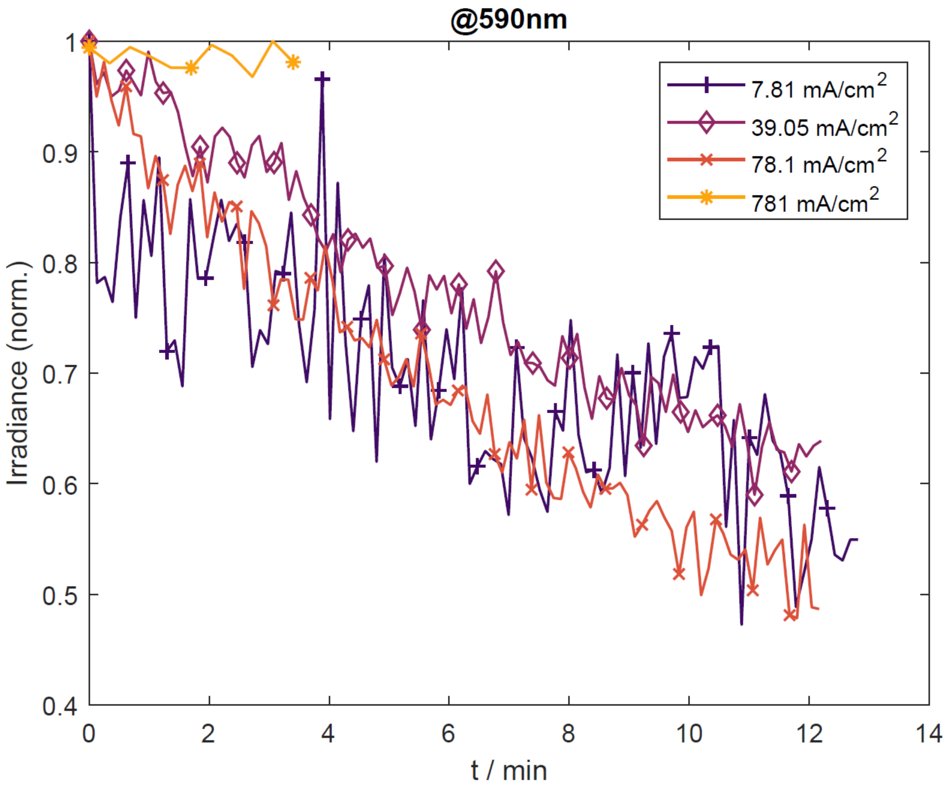

Figure S81. Temporal decay of the light output of an OLED (glass/ITO/PEDOT:PSS/**2**+**3**(3:1)/PO-T2T/LiF/Al) in air at different injection current densities employing a 3:1 mixture of **2** and **3** as EML spin-coated from a solution in THF with a concentration of 2 mg/mL.

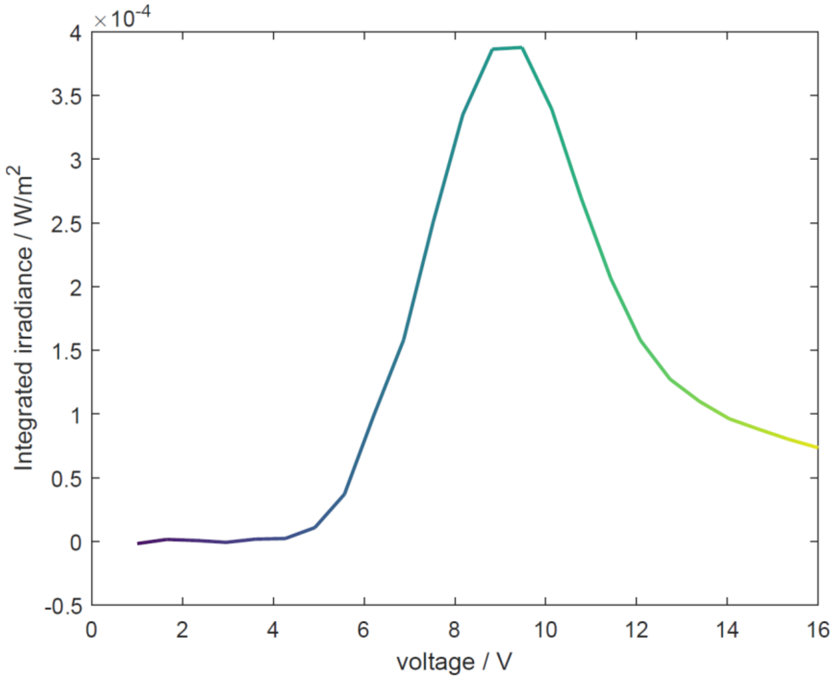

Figure S82. Integrated EL intensity obtained from a voltage sweep of an OLED (glass/ITO/PEDOT:PSS/**2**+**3**(3:1)/PO-T2T/LiF/Al) employing a 3:1 mixture of **2** and **3** as EML spin-coated from a solution in THF with a concentration of 2 mg/mL.

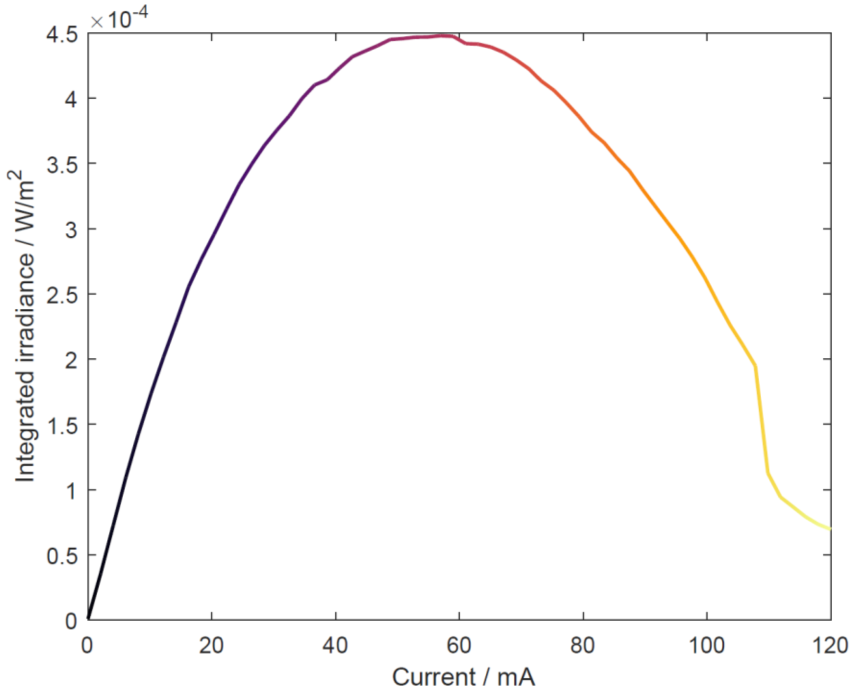

Figure S83. Integrated EL intensity obtained from a current sweep of an OLED (glass/ITO/PEDOT:PSS/2+3(3:1)/PO-T2T/LiF/Al) employing a 3:1 mixture of **2** and **3** as EML spin-coated from a solution in THF with a concentration of 2 mg/mL.

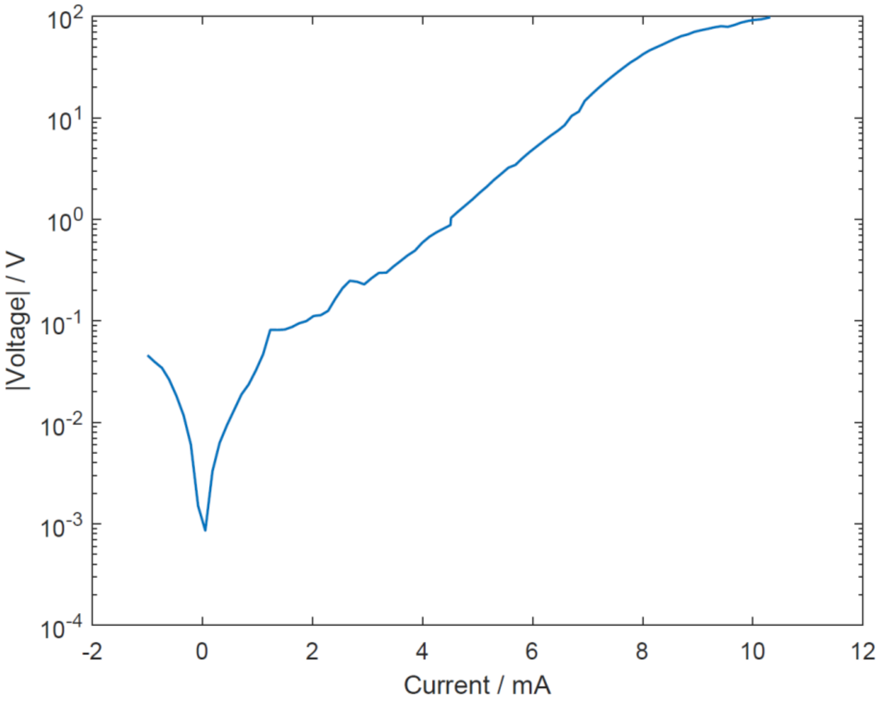

Figure S84. *J*-*V* curve of an OLED (glass/ITO/PEDOT:PSS/**2**+**3**(3:1)/PO-T2T/LiF/Al), employing a 3:1 mixture of **2** and **3** as EML spin coated from a solution in THF with a concentration of 2 mg/mL.

ChampionOLED_EyeImpression.mp4

Video S5. Visual impression of one of the best OLEDs constructed with compound **1** as the emitter.

Quantum chemistry

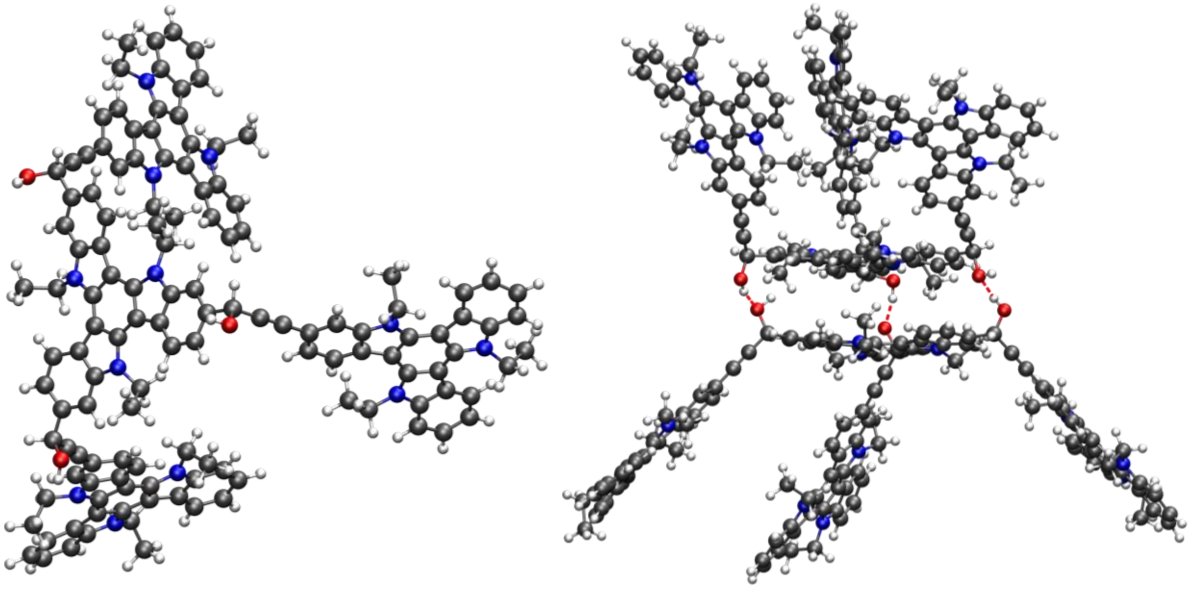

Figure S85. DFT calculated structures of a monomer (SSR enantiomer) (left) and dimer (right) of **1** with marked H-bonds.

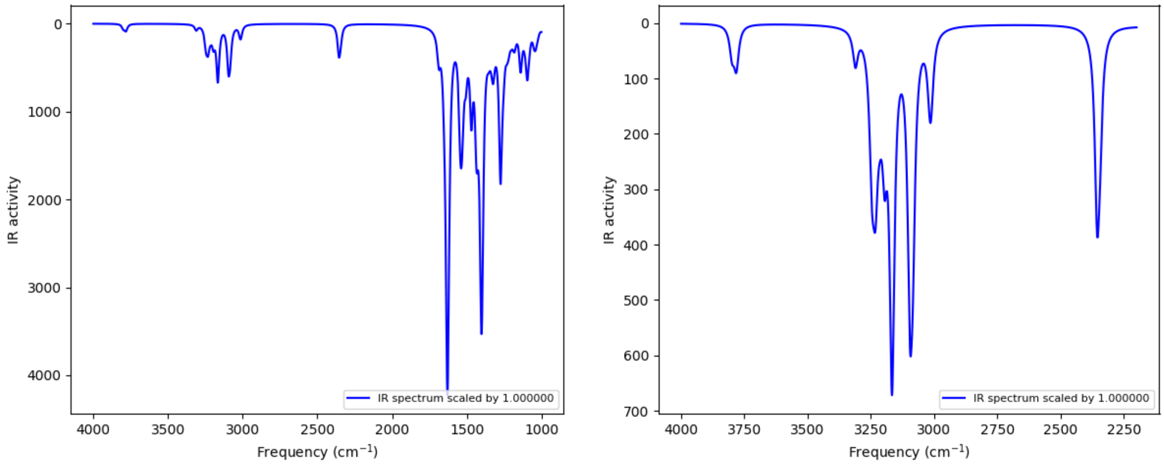

Figure S86. DFT calculated IR spectrum of an individual molecule of **1** with O-H vibrations and the C≡C stretching vibration enlarged on the right and a FWHM of 23 cm^{-1}.

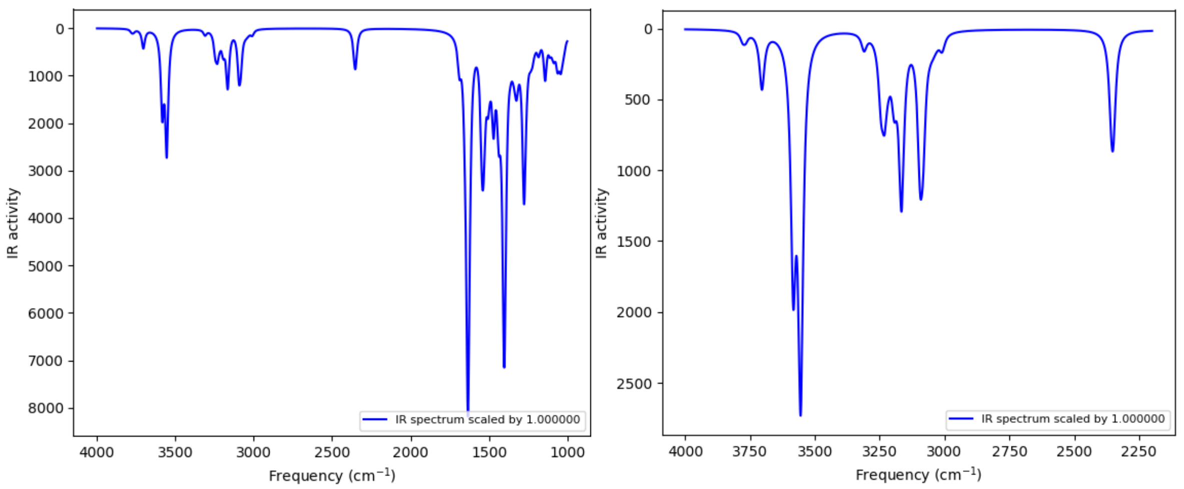

Figure S87. DFT calculated IR spectrum of a dimer of **1** with O-H vibrations and the C≡C stretching vibration enlarged on the right and a FWHM of 23 cm^{-1}.

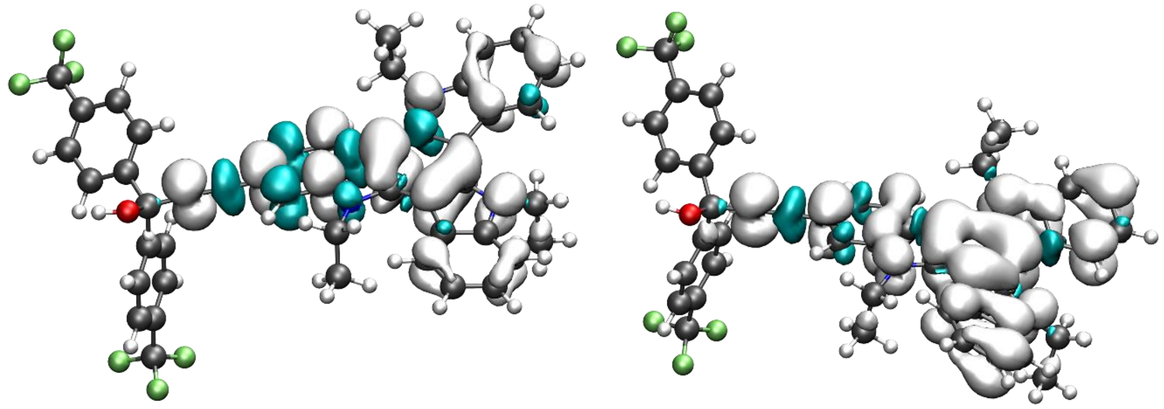

Figure S88. DFT-calculated spin densities of **2**$^{+}$ (left) and **2**$^{2+}$ (right, triplet state).

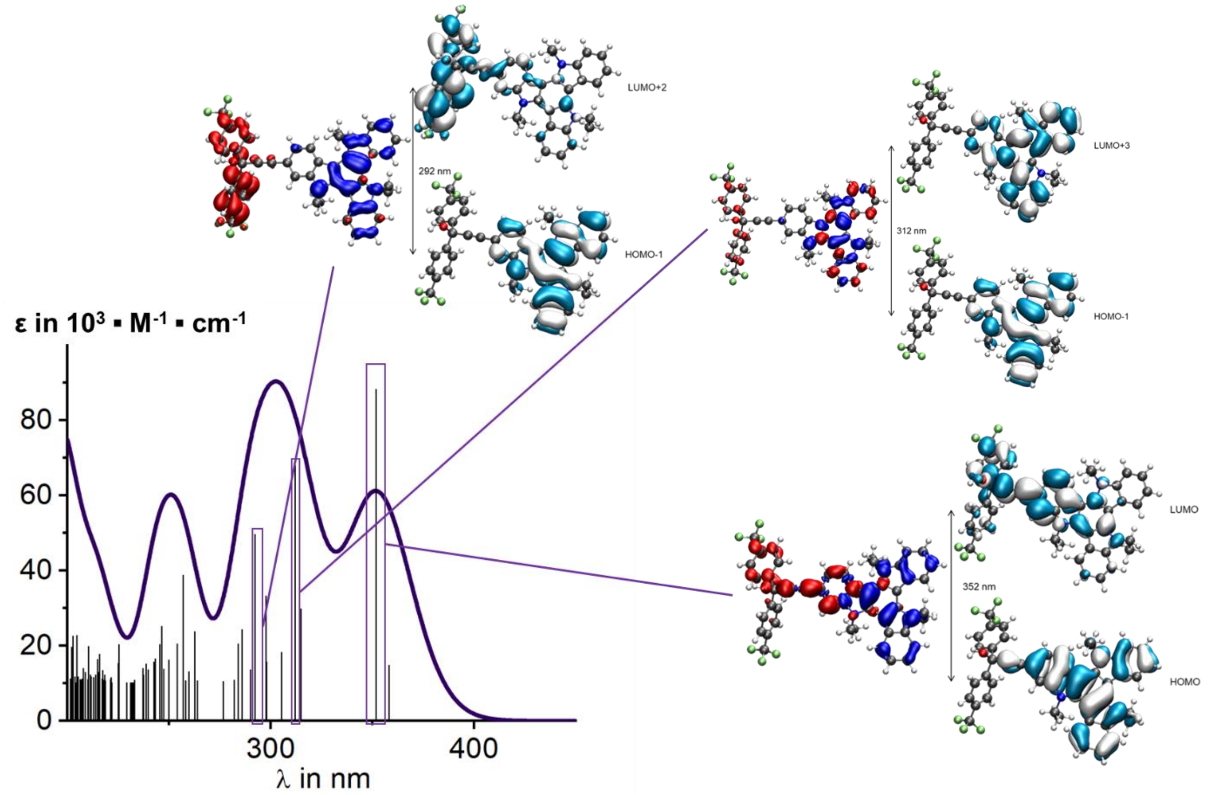

Figure S89. TD-DFT-calculated UV/vis/NIR spectrum of **2** with the electronic transitions and the respective donor and acceptor molecular orbitals including the corresponding EDDM plots. Molecular orbitals are depicted in blue and white for positive and negative signs. EDDMs are depicted in red for electron density gain and blue for electron density loss.

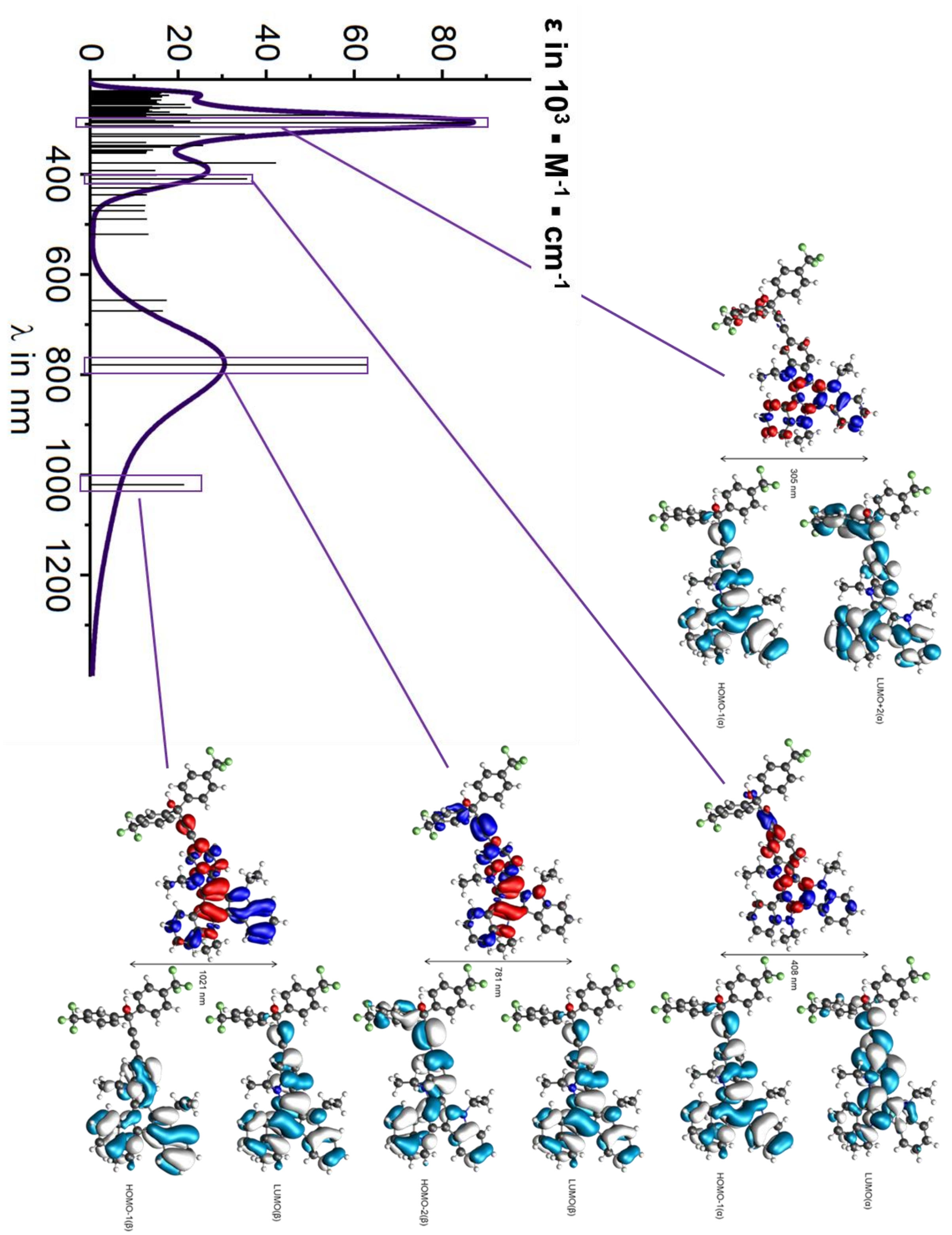

Figure S90. TD-DFT-calculated UV/vis/NIR spectrum of **2**$^{+}$ with the electronic transitions and the respective donor and acceptor molecular orbitals including the corresponding EDDM plots. Molecular orbitals are depicted in blue and white for positive and negative signs. EDDMs are depicted in red for electron density gain and blue for electron density loss.

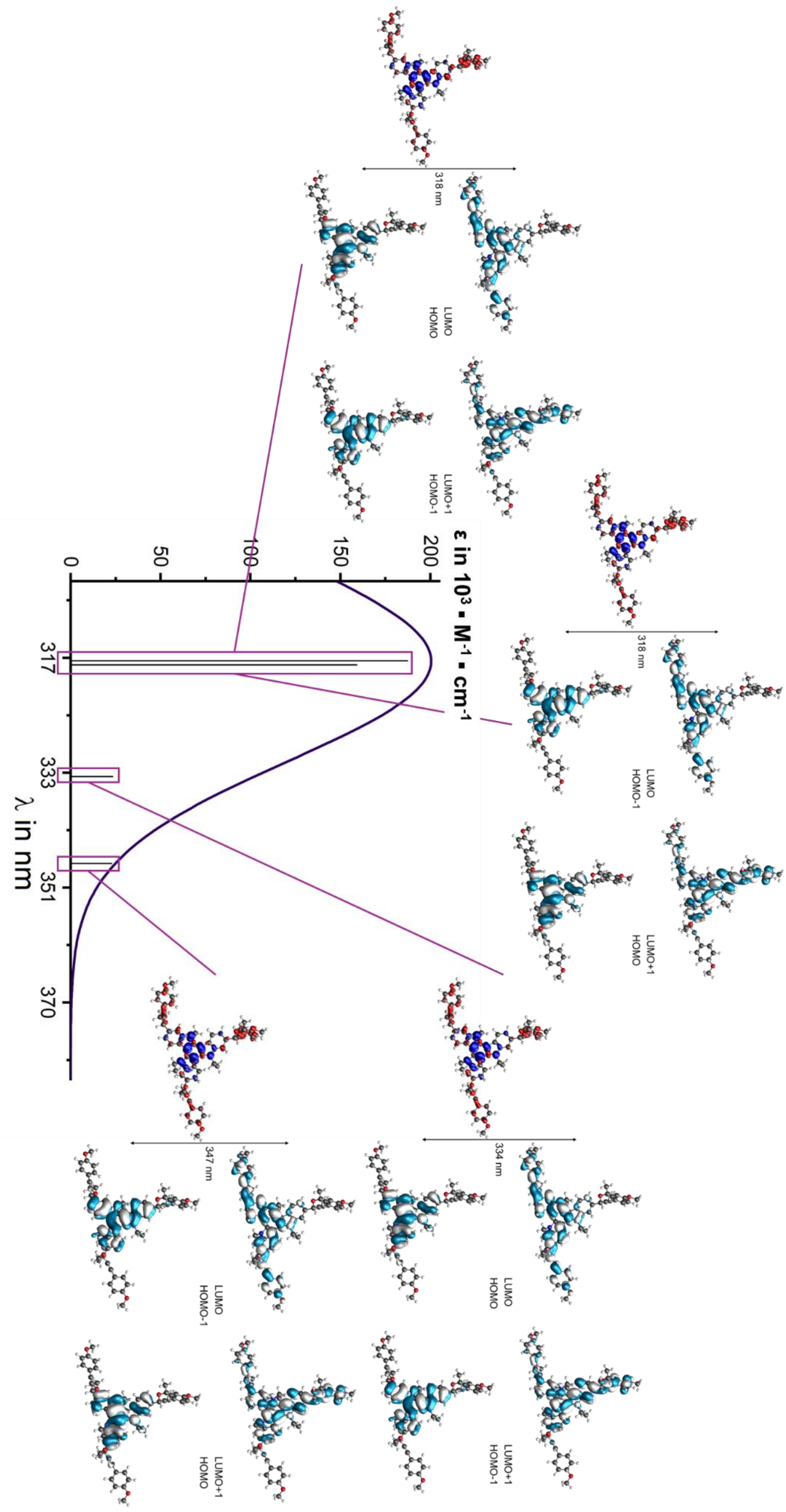

Figure S91. TD-DFT-calculated UV/vis/NIR spectrum of **3** with the electronic transitions and the respective donor and acceptor molecular orbitals including the corresponding EDDM plots. Molecular orbitals are depicted in blue and white for positive and negative signs. EDDMs are depicted in red for electron density gain and blue for electron density loss.

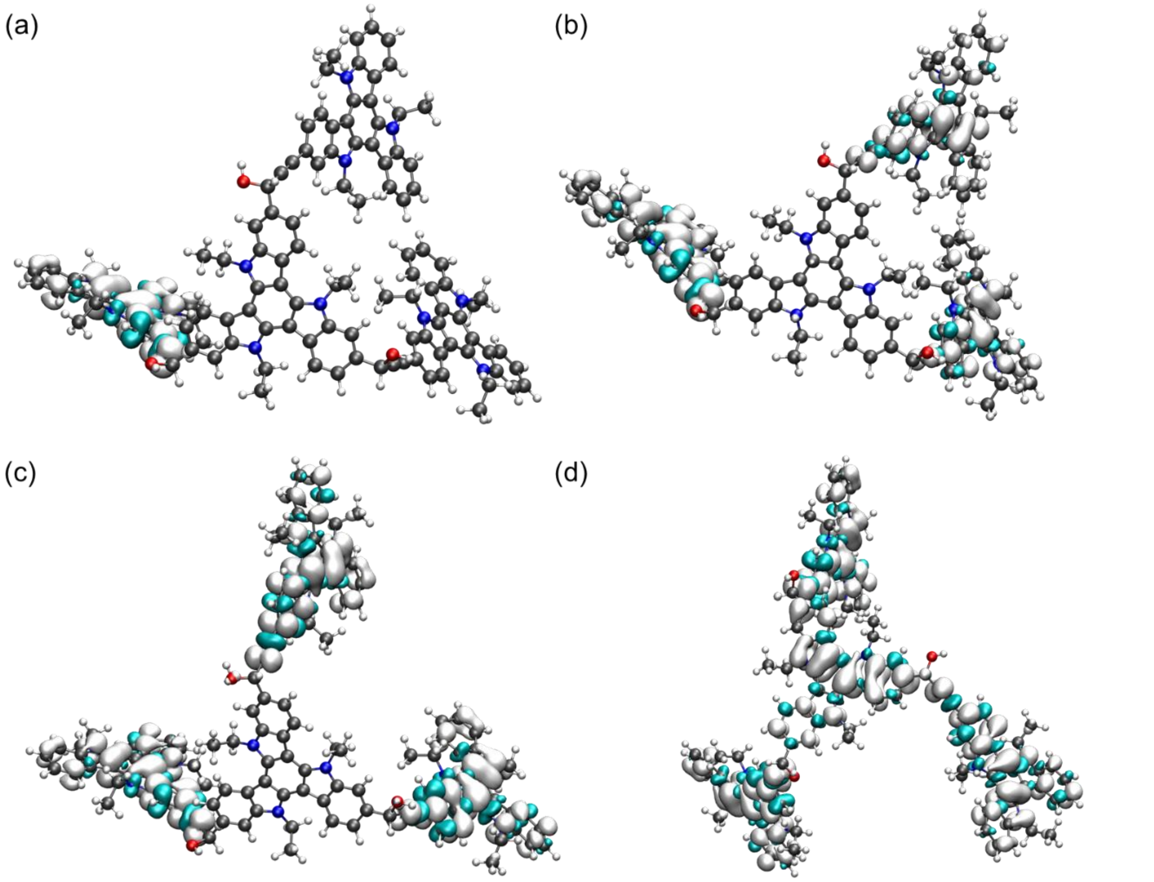

Figure S92. DFT-calculated spin densities of $\mathbf{1}^{+}$ (top left), $\mathbf{1}^{2+}$ (top right, triplet state), $\mathbf{1}^{3+}$ (bottom left, quartet state), and $\mathbf{1}^{4+}$ (bottom right, quintet state).

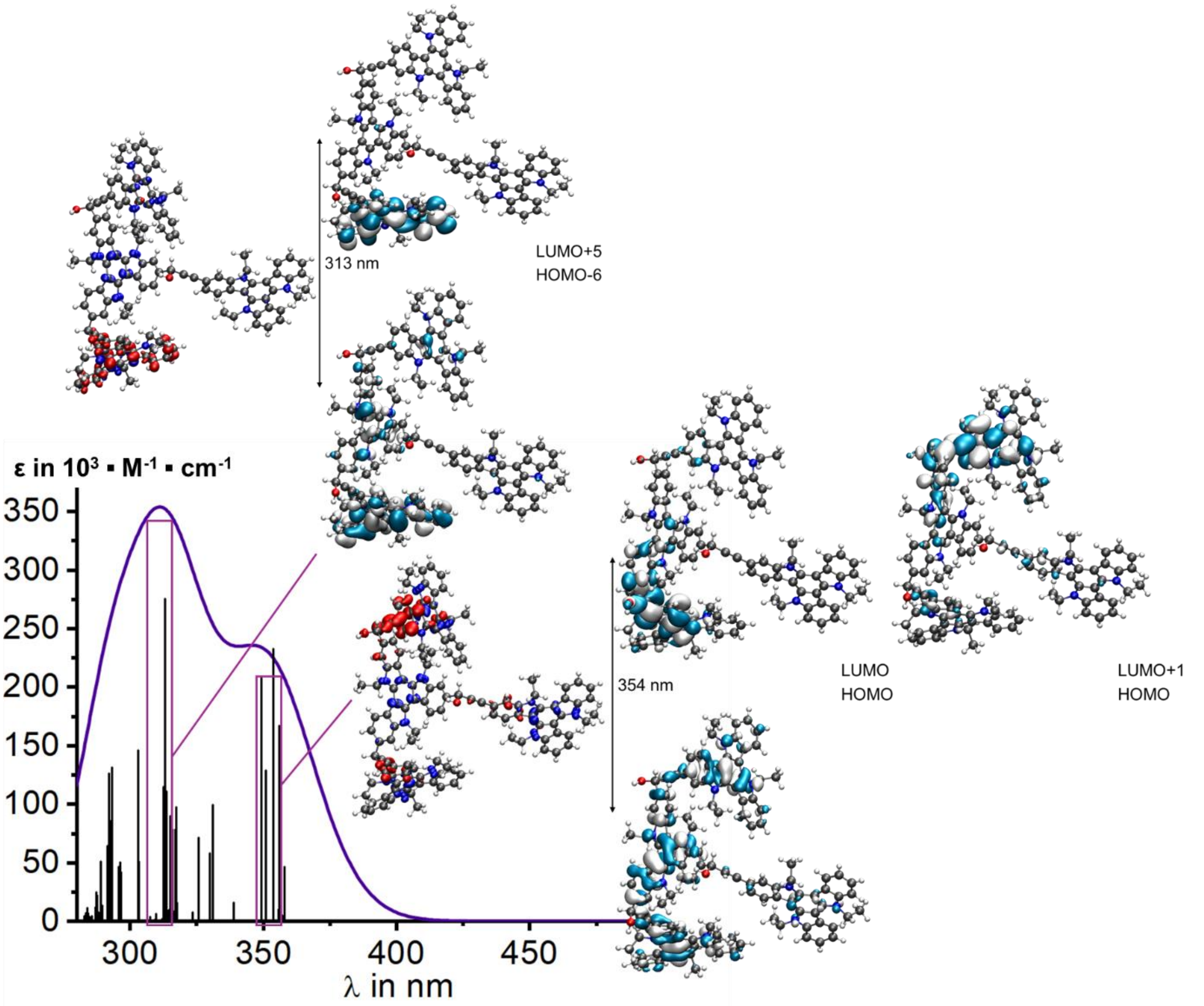

Figure S93. TD-DFT-calculated UV/vis/NIR spectrum of **1** with the electronic transitions and the respective donor and acceptor molecular orbitals including the corresponding EDDM plots. Molecular orbitals are depicted in blue and white for positive and negative signs. EDDMs are depicted in red for electron density gain and blue for electron density loss.

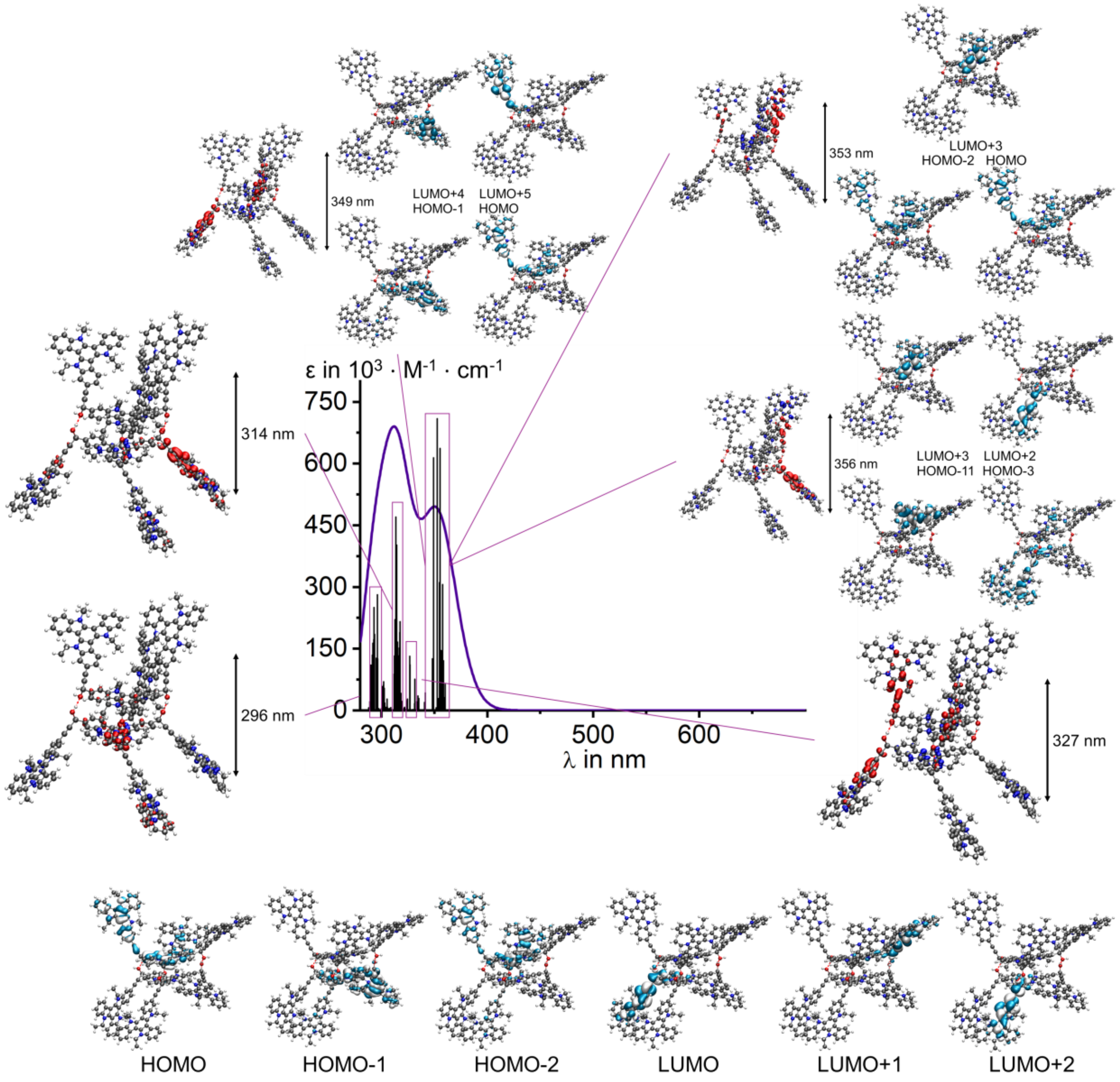

Figure S94. TD-DFT-calculated UV/vis/NIR spectrum of a dimer of **1** with the electronic transitions and the respective donor and acceptor molecular orbitals including the corresponding EDDM plots. Molecular orbitals are depicted in blue and white for positive and negative signs. EDDMs are depicted in red for electron density gain and blue for electron density loss.

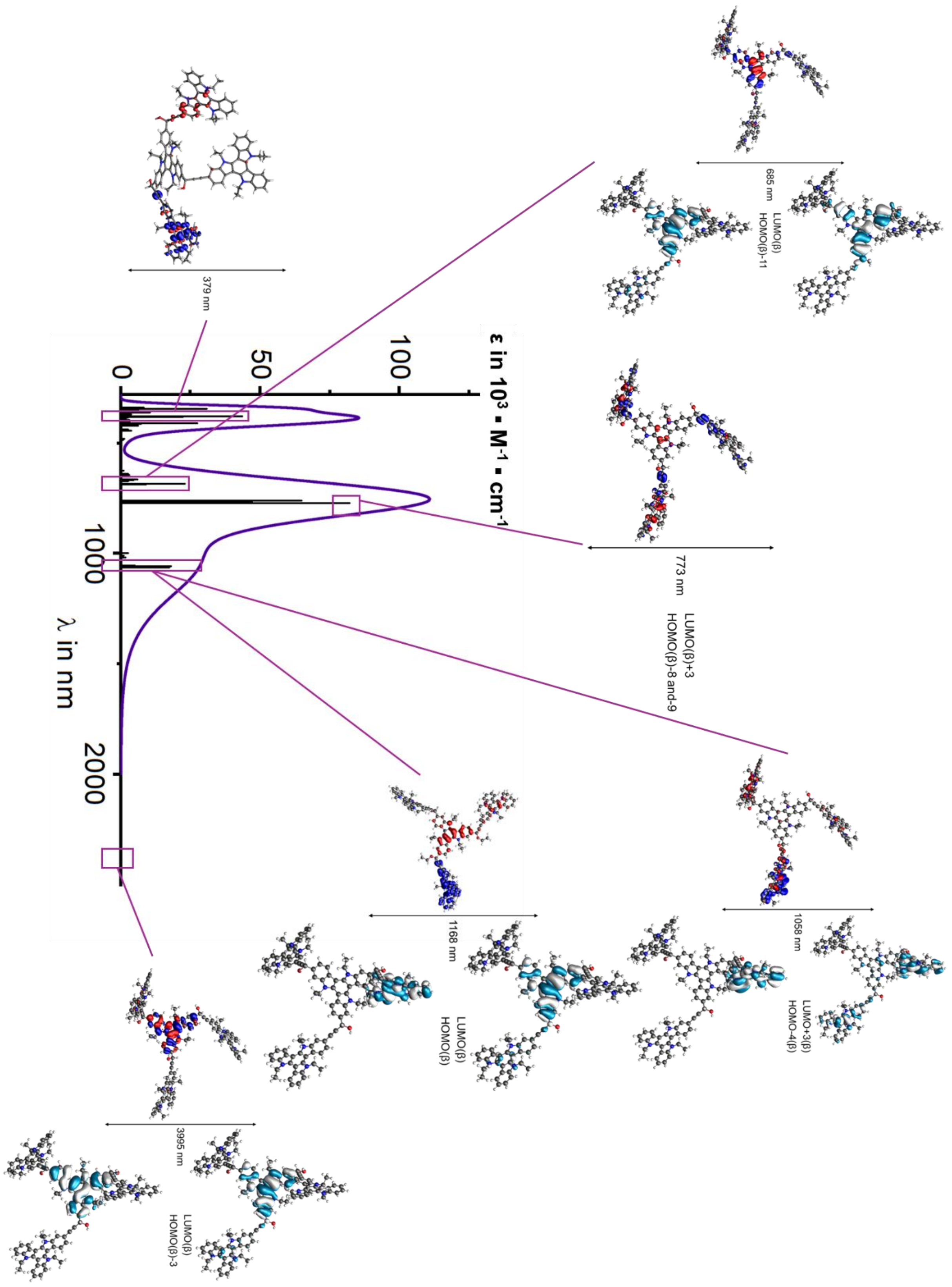

Figure S95. TD-DFT-calculated UV/vis/NIR spectrum of $\mathbf{1}^{4+}$ (quintet state) with the electronic transitions and the respective donor and acceptor molecular orbitals including the corresponding EDDM plots. Molecular orbitals are depicted in blue and white for positive and negative signs. EDDMs are depicted in red for electron density gain and blue for electron density loss.

References

[59] A. Bauer, M. Maier, W. M. Schosser, J. Diegel, F. Paschke, Y. Dedkov, F. Pauly, R. F. Winter, M. Fonin, *Adv. Mater.* **2020**, *32*, e1907390.

[64] Y.-F. Xie, S.-Y. Ding, J.-M. Liu, W. Wang, Q.-Y. Zheng, *J. Mater. Chem. C* **2015**, *3*, 10066.

[66] L. Vogelsang, T. Birk, F. Paschke, A. Bauer, V. Enenkel, L. M. Holz, M. Fonin, R. F. Winter, *Inorg. Chem.* **2023**, *62*, 16236.

[93] D. L. Reger, T. D. Wright, C. A. Little, J. J. Lamba, M. D. Smith, *Inorg. Chem.* **2001**, *40*, 3810.

[94] Y. Li, M. Josowicz, L. M. Tolbert, *J. Am. Chem. Soc.* **2010**, *132*, 10374.

[95] M. Krejčik, M. Daněk, F. Hartl, *J. Electroanal. Interfac. Electrochem.* **1991**, *317*, 179.

[96] M. J. Frisch, G. W. Trucks, H. B. Schlegel, G. E. Scuseria, M. A. Robb, J. R. Cheeseman, G. Scalmani, V. Barone, B. Mennucci, G. A. Petersson, H. Nakatsuji, M. Caricato, X. Li, H. P. Hratchian, A. F. Izmaylov, J. Bloino, G. Zheng, J. L. Sonnenberg, M. Hada, M. Ehara, K. Toyota, R. Fukuda, J. Hasegawa, M. Ishida, T. Nakajima, Y. Honda, O. Kitao, H. Nakai, T. Vreven, J. A. Montgomery Jr., J. E. Peralta, F. Ogliaro, M. J. Bearpark, J. Heyd, E. N. Brothers, K. N. Kudin, V. N. Staroverov, R. Kobayashi, J. Normand, K. Raghavachari, A. P. Rendell, J. C. Burant, S. S. Iyengar, J. Tomasi, M. Cossi, N. Rega, N. J. Millam, M. Klene, J. E. Knox, J. B. Cross, V. Bakken, C. Adamo, J. Jaramillo, R. Gomperts, R. E. Stratmann, O. Yazyev, A. J. Austin, R. Cammi, C. Pomelli, J. W. Ochterski, R. L. Martin, K. Morokuma, V. G. Zakrzewski, G. A. Voth, P. Salvador, J. J. Dannenberg, S. Dapprich, A. D. Daniels, Ö. Farkas, J. B. Foresman, J. V. Ortiz, J. Cioslowski, D. J. Fox, Gaussian, *Inc.: Wallingford,CT, USA,2009*.

[97] A. D. McLean, G. S. Chandler, *J. Chem. Phys.* **1980**, *72*, 5639.

[98] J. P. Perdew, K. Burke, M. Ernzerhof, *Phys. Rev. Lett.* **1996**, *77*, 3865.

[99] S. Grimme, S. Ehrlich, L. Goerigk, *J. Comput. Chem.* **2011**, *32*, 1456.

[100] M. Cossi, N. Rega, G. Scalmani, V. Barone, *J. Comput. Chem.* **2003**, *24*, 669.

[101] F. Paschke, P. Erler, L. Gragnaniello, J. Dreiser, M. Fonin, *Quantum Mat. Res.* **2020**.

[102] L. Ji, Q. Fang, M.-S. Yuan, Z.-Q. Liu, Y.-X. Shen, H.-F. Chen, *Org. Lett.* **2010**, *12*, 5192.

[103] R. Evans, Z. Deng, A. K. Rogerson, A. S. McLachlan, J. J. Richards, M. Nilsson, G. A. Morris, *Angew. Chem.* **2013**, *125*, 3281.

[104] R. Neufeld, D. Stalke, *Chem. Sci.* **2015**, *6*, 3354.

[105] J. R. Rumble (Ed.), *CRC Handbook of Chemistry and Physics*, CRC Press, Boca Raton, London, New York **2021**.

[106] M. Matsumoto, T. W. Swaddle, *Inorg. Chem.* **2004**, *43*, 2724.

[107] A. Bauer, T. Birk, F. Paschke, A. Fuhrberg, J. Diegel, A.-K. Becherer, L. Vogelsang, M. Maier, W. M. Schosser, F. Pauly, O. Zilberberg, R. F. Winter, M. Fonin, *Adv. Mater.* **2024**.
